# Methyltransferase-assisted single duplex sequencing for detecting circulating tumor DNA

**DOI:** 10.1038/s44321-026-00509-2

**Published:** 2026-08-19

**Authors:** Zhaoyu Jiang, Yuxuan Wu, Yuqing Guo, Renjie Luo, Wei Wang, Fuchou Tang, Wei Fu, Xin Zhou, Lu Wen

**Affiliations:** 1 Department of General Surgery, Biomedical Pioneering Innovation Center, Third Hospital, Peking University, Beijing, 100871 China; 2 Peking University Third Hospital Cancer Center, Beijing, 100191 China; 3 Beijing Key Laboratory of Collaborative Innovation in Gastrointestinal Oncology, Beijing, 100191 China; 4 Beijing Advanced Innovation Center for Genomics (ICG), Ministry of Education Key Laboratory of Cell Proliferation and Differentiation, School of Life Sciences, Peking University, Beijing, 100871 China

**Keywords:** Biomarkers, Cancer

## Abstract

Accurate circulating tumor DNA (ctDNA) detection is limited by high sequencing errors. To address this, we developed Methyltransferase-Assisted Single Duplex sequencing (MASD-seq), which physically links Watson and Crick strands to enable error correction using double-stranded information within a single read pair. MASD-seq employs enzymatic methyl-sequencing to disrupt strand pairing and uses CpG methyltransferase pre-methylation to enable detection of CpG>TpG and transversion mutations in DNA duplexes. In parallel, MspI digestion enriches tumor CpG>TpG mutations and reduces genome complexity. MASD-seq achieves error rates of 5.4 × 10^−7^ for CpG>TpG and 4.1 × 10^−8^ for transversions in cell lines, allowing detection of age-associated mutational burdens in blood. Tissue analyses show a 4-fold enrichment of CpG>TpG mutations and a 1.6-fold increase in total mutations in high microsatellite instability (MSI-H) colorectal cancer (CRC), and 3-fold and 1.2-fold enrichments in microsatellite stable (MSS) tumors, respectively, yielding a correctable mutation burden comparable to the whole genome. Clinical testing demonstrates an area under the curve (AUC) of 0.97 for ctDNA detection in CRC patients. MASD-seq is a highly accurate sequencing method, suggesting its potential for future clinical applications.

## Synopsis

This study presents MASD-seq, a duplex sequencing method for highly accurate circulating tumor DNA (ctDNA) detection. The approach is combined with cost-efficient tumor mutation profiling in tissue samples for minimal residual disease monitoring.

- Duplex error correction from a single read pair is enabled by MASD-seq through physical linkage of DNA strands, coupled with methylation conversion and M.SssI protection.
- Error rates of 5.4 × 10^−7^ for CpG>TpG and 4.1 × 10^−8^ for transversions are achieved by MASD-seq in cell lines, and age-associated mutational burdens are detected in blood.
- CpG>TpG mutations in colorectal cancer are enriched up to 4-fold by MspI-RRS, capturing a correctable mutation burden comparable to that of the whole-genome sequencing.
- Colorectal cancer patients are distinguished from healthy controls with high accuracy (AUC = 0.97) using this approach.

## Abstract (web summary)

This study presents MASD-seq, a duplex sequencing method for highly accurate circulating tumor DNA (ctDNA) detection. The approach is combined with cost-efficient tumor mutation profiling in tissue samples for minimal residual disease monitoring.

## Graphical abstract

**Figure:**
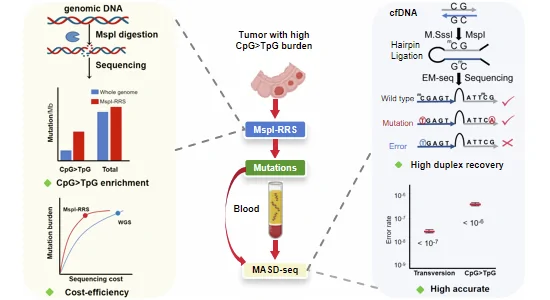

The paper explained **Problem**

Monitoring cancer patients for signs of relapse using a simple blood test—often called “liquid biopsy”—holds great promise. However, these tests must detect extremely rare tumor DNA hidden among vast amounts of normal DNA. This task is made especially difficult because the sequencing process itself frequently introduces errors that can be mistaken for real cancer mutations. The current most accurate method, known as duplex sequencing, overcomes this issue by comparing both strands of a DNA molecule. But this approach is inefficient as the original two strands tend to become separated during sample preparation, making the test less practical for routine use.

**Results**

To address this inefficiency, we developed a new duplex method, MASD-seq, which physically links the two strands of DNA together throughout the entire workflow. Sequencing this covalently stabilized structure posed an additional technical challenge, which we overcame by applying a methylation-conversion approach that uses the CpG methyltransferase M.SssI to protect CpG sites. In parallel, we implemented a targeted enrichment strategy, termed MspI-RRS, designed to enrich tumor-derived CpG>TpG mutations.

In technical validation experiments on cell lines, MASD-seq achieved low error rates of 5.4 × 10^−7^ for CpG>TpG and 4.1 × 10^−8^ for transversions. This level of precision allowed us to detect age-related mutational changes in blood samples from healthy individuals. When we applied MASD-seq to tumor tissues, the results showed a 4-fold enrichment of CpG>TpG mutations in high microsatellite instability (MSI-H) colorectal cancer and a 3-fold enrichment in microsatellite stable (MSS) tumors. Overall, the total number of correctable mutations reached a level comparable to what whole-genome sequencing would provide. In a preliminary clinical validation study using blood samples from colorectal cancer patients, our method distinguished patients from healthy controls with high accuracy (AUC = 0.97).

**Impact**

Our study presents a novel duplex sequencing method for highly accurate ctDNA detection, which can be coupled with cost-efficient mutation profiling in tumor tissue. This approach holds promise for future clinical applications, especially for monitoring treatment response in MSI-H patients undergoing immunotherapy, thereby supporting more informed clinical decision-making.

## Introduction

Monitoring of minimal residual disease (MRD) via circulating tumor DNA (ctDNA) has emerged as a promising approach to improve patient outcomes. For example, positive ctDNA detection after surgery in colorectal cancer (CRC) patients has been shown to be an independent prognostic factor for relapse, making it a criterion for chemotherapy recommendation (Kotani et al, 2023; Tie et al, 2022). However, the accuracy of ctDNA detection is limited by background sequencing errors, which originate from various sources including pre-analytical factors such as cell-free DNA (cfDNA) damage in blood samples, errors introduced during library preparation such as polymerase and target-capture errors, and sequencing artifacts (Arbeithuber et al, 2016; Lee-Six et al, 2019; Newman et al, 2016; Song et al, 2022). Duplex sequencing, which identifies somatic variants on both complementary strands of parental DNA duplexes, is currently the gold standard for accuracy, achieving error rates of 10^−7^ to 10^−9^ per base (Abascal et al, 2021; Hoang et al, 2016; Kennedy et al, 2014a; Schmitt et al, 2012). However, this approach is hampered by the low efficiency of DNA duplex recovery after the original strands separate during library preparation (Abascal et al, 2021; Kennedy et al, 2014b; Newman et al, 2016; Parsons et al, 2020; Wang et al, 2019).

To address this issue, a promising strategy is to link the parental DNA duplex, thereby enabling single-read error correction. Previous studies have reported circle sequencing (Lou et al, 2013), o2n-seq (Wang et al, 2017), and AccuScan (Li et al, 2024), which physically link fragments from original molecules to form consensus sequences. However, these methods use only a single strand of a double-stranded molecule. PhasED-seq utilizes multiple mutations within individual DNA fragments, though the number of usable mutations remains limited (Kurtz et al, 2021). HiDEF-seq utilizes a single-molecule, long-read sequencing method to repeatedly sequence both strands of the original DNA molecule but is expensive and has not been applied to short cfDNA fragments (Liu et al, 2024). A notable recent advance is the single-duplex method CODEC, which physically concatenates both strands into a single strand, achieving single-duplex sequencing on the next-generation sequencing (NGS) platform, although its molecular capture efficiency remains suboptimal (Bae et al, 2023). Another method, five-letter seq, utilizes a helicase-assisted enzymatic-methyl sequencing (EM-seq) approach to sequence hairpin structures (Fullgrabe et al, 2023). Nevertheless, this method relies on single-strand genetic information and is designed to simultaneously detect genetic bases and epigenetic modifications (Fullgrabe et al, 2023).

For profiling tumor tissue mutations, the primary approaches are targeted panels, whole exome sequencing (WES) and whole genome sequencing (WGS). Reduced representation sequencing (RRS), which utilizes restriction enzyme digestion to reduce genome complexity, has rarely been reported (Andrews et al, 2016). RRS offers a simple approach to save sequencing cost, and also has the potential to enrich for tumor mutations. Since many human cancers are characterized by high frequency of CpG>TpG mutations, it would be valuable to investigate whether CpG-targeted restriction enzyme digestion could enrich for such mutations (Gu et al, 2011).

Here, we present Methyltransferase-Assisted Single Duplex sequencing (MASD-seq) that enables single-read error correction by physically linking the parental DNA duplex. We also developed a CpG-targeted RRS method that reduces genome complexity and enriches for tumor mutations. We applied these methods to clinical samples from CRC patients, with a primary focus on MSI-H patients. Because enzymatic cytosine conversion inherently limits correction of certain transition classes, MASD-seq was designed to focus on mutation types that can be robustly corrected by duplex information, including CpG>TpG mutations protected by M.SssI methylation and transversions. We therefore evaluated both the analytical accuracy of these correctable mutation classes and their practical utility for ctDNA detection.

## Results

### Development of MASD-seq

**Figure 1:**
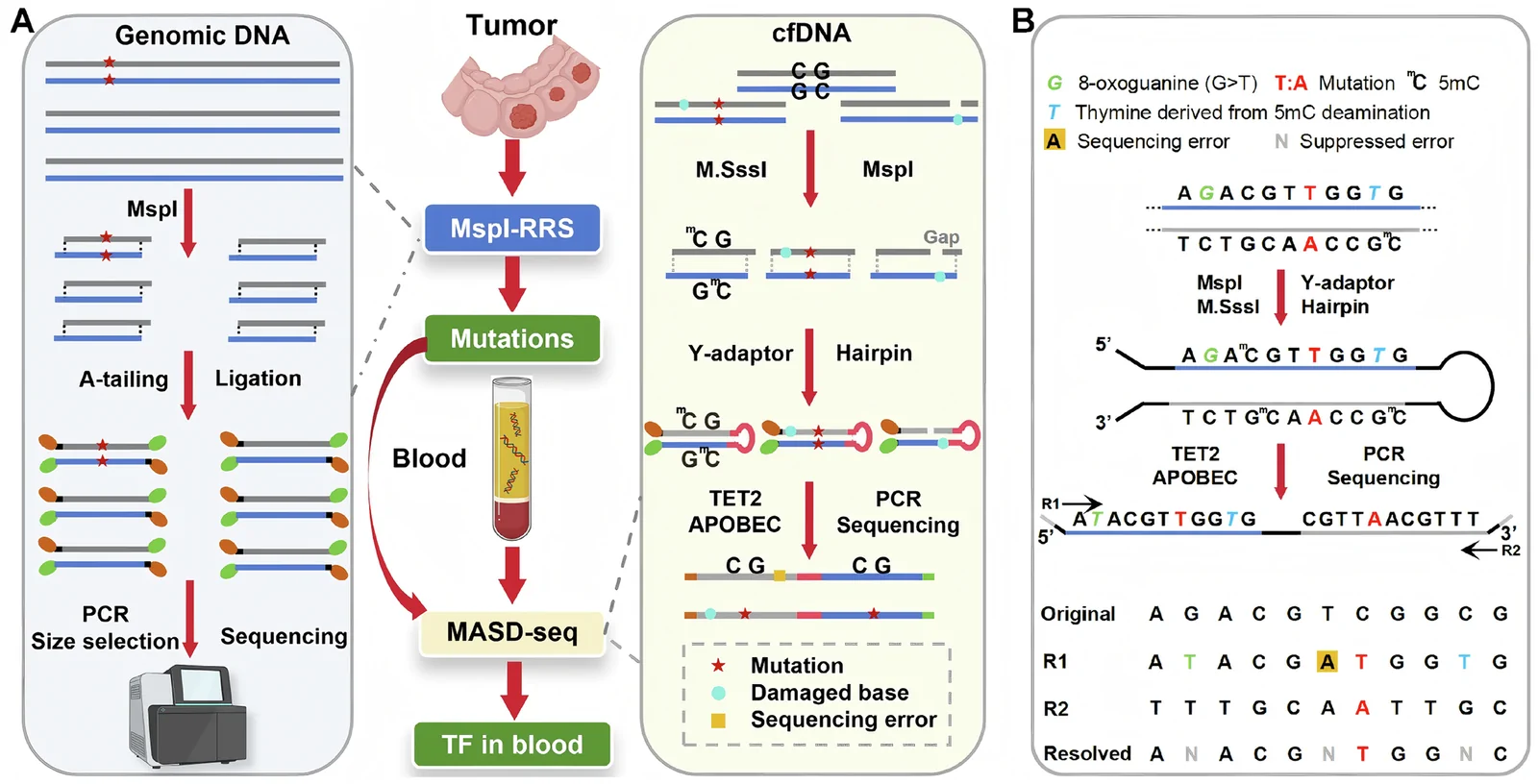
Workflow schematic for MASD-seq and MspI-RRS. (**A**) For tissue samples, a RRS assay using the restriction enzyme MspI (recognizing C|CGG, MspI-RRS) is performed to identify tumor-specific mutations (left panel). The MspI digestion enriches for both the CpG>TpG mutation burden and the total mutation burden while reducing genome complexity. For plasma cfDNA, MASD-seq, which enables efficient single-read error correction through physically linking the original DNA double strands, is employed to quantify the tumor fraction (TF, which is the ratio of tumor mutant alleles to the total number of alleles) in blood (right panel). (**B**) MASD-seq involves three main steps: (1) following MspI digestion, hairpin adapters are ligated to double-stranded DNA; (2) CpG methyltransferase (M.SssI) methylates all CpG sites; and (3) TET2 and APOBEC/UvrD helicase convert unmethylated cytosines to uracil to enable PCR amplification and sequencing. Physical linkage of Watson-Crick strands via hairpin adapters enables error correction in a single-duplex read. Since all cytosines in CpG dinucleotides remain methylated (and thus protected), the method enables correction of the CpG>TpG error, as well as all transversion errors (C>A, C>G, T>G, and T>A). The resolve rules dictated that non-permissible pairs, such as TpG-CpG, T-T, and A-A, were masked (marked as N) at the affected position.

To achieve highly accurate sequencing, we developed MASD-seq that enables single-read error correction by physical ligation of the original Watson and Crick strands of a DNA duplex (Fig. 1). MASD-seq is modified from five-letter seq, an EM-seq-based method. While five-letter seq utilizes only single-strand genetic information, MASD-seq preserves genetic information from both strands of the original DNA molecule, aiming to achieve “single-duplex” sequencing. As this method disrupts complementary strand pairing for sequencing, the correction of C>T and A>G mutations is compromised. To address this, we employ the CpG methyltransferase M.SssI to methylate CpG sites before EM-seq, blocking their conversion, and thereby enabling correction of CpG>TpG mutations—a mutation type prevalent in human cancers including CRC (Alexandrov et al, 2020; Alexandrov et al, 2013; Cancer Genome Atlas Research, 2014; Cancer Genome Atlas Research et al, 2013; Cortes-Ciriano et al, 2017; Lee-Six et al, 2019; Silveira et al, 2024). We also utilized MspI digestion (recognizing C|CGG) to enrich for tumor CpG>TpG mutations. As will be shown later, we found that the MspI-digested regions are enriched not only for the CpG>TpG mutations, but also for the overall mutation number per Mb in CRC. The operation of restriction enzyme digestion also avoids the need for end-repair step and helps ensure duplex accuracy (Abascal et al, 2021). For tissue samples, as MASD-seq targets the MspI-digested region, we developed a RRS assay using MspI digestion (MspI-RRS) to reduce genome complexity and save sequencing costs. Alternatively, WGS can be used to comprehensively profile tumor tissue mutations (Fig. 1A).

The MASD-seq library preparation process was as follows (Fig. 1). First, the DNA was digested with MspI, and within the same reaction, M.SssI was added to methylate all CpGs to 5mCpGs. Then, the MspI-digested fragments were ligated to a combination of a biotin-labeled hairpin adapter and a Y adapter, which differed from five-letter seq that used only the hairpin adapter, yet resembling the hairpin-bisulfite sequencing method (Fullgrabe et al, 2023; Zhao et al, 2014). Random nucleotide sequences were incorporated within the hairpin adapter to serve as unique molecule identifiers (UMIs). After ligation, biotin-labeled fragments were selectively pulled down. Subsequent processing followed the UvrD helicase-assisted EM-seq protocol of five-letter seq. Briefly, the 5mCpGs underwent enzymatic protection through a series of conversions: oxidation to 5hmCpGs, 5fCpGs, or 5caCpGs by Tet methylcytosine dioxygenase 2 (TET2), followed by enzymatic glycosylation of 5hmCpGs by beta-glycosyltransferase. Unprotected cytosines were then deaminated to uracil (subsequently read as thymine) by APOBEC3A (Fullgrabe et al, 2023; Vaisvila et al, 2021). Since APOBEC3A acted exclusively on single-stranded DNA, UvrD helicase was incorporated to generate single-stranded DNA substrates. Finally, this C>T transformation separated the complementary strands, allowing for PCR amplification and sequencing. The CpGs are not transformed as they have been initially methylated.

The theoretical probability of simultaneous errors occurring at the same position on both strands was the square of the single-strand error probability, leading to substantially improved detection accuracy via duplex consensus. The resolution rule dictated that non-permissible pairs, were masked (marked as N) at the affected position (Fig. 1B). Through this correction mechanism, MASD-seq effectively corrects the CpG>TpG error and all transversion errors (C>A, C>G, T>G, and T>A, in which the mutated base is represented by the pyrimidine of the base pair). The transition errors including CpH>TpH (where H represents A, T or C) and T>C remained uncorrectable, and we defined mutations excluding CpH>TpH and T>C types as “correctable” mutations.

### Validation of MASD-seq

**Figure 2:**
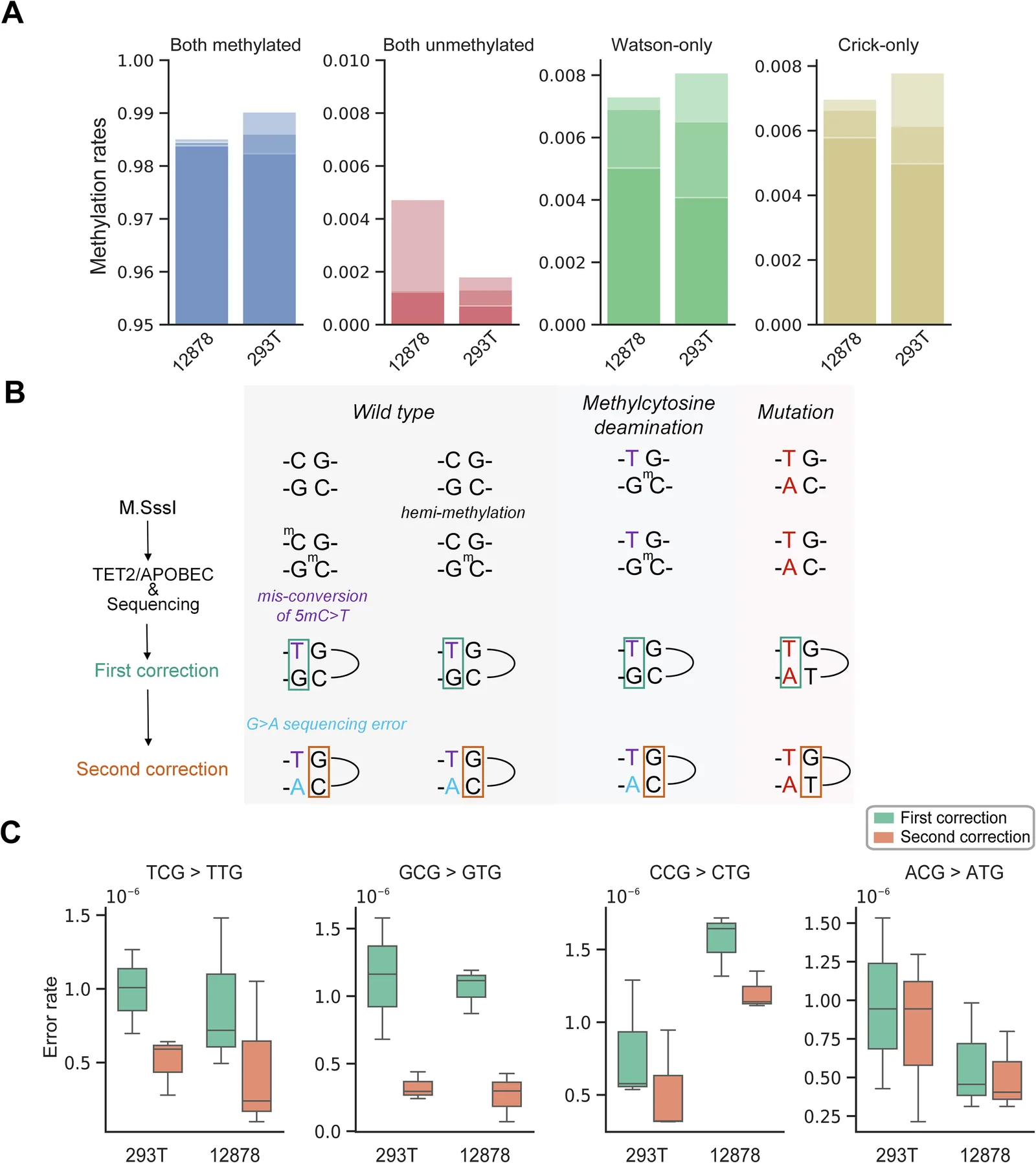
Double CpG correction improves accuracy. (**A**) DNA methylation status in GM12878 and HEK293T cell lines following methyltransferase treatment. Four patterns are shown: methylated on both strands, unmethylated on both strands, and methylated on a single strand. Different shades represent three independent replicates. (**B**) Schematic of the double-CpG correction algorithm. Three scenarios are illustrated: wild-type CpG sites, methylcytosine deamination, and genuine mutation. In wild-type molecules, false-positive CpG>TpG signals could arise from mis-conversion of 5mC to T by incomplete TET2 oxidation or hemi-methylation of M.SssI. Methylcytosine deamination also leads to a hemi-CpG state. In all these situations, one CpG dinucleotide is retained on the complementary strand. In contrast, a genuine mutation disrupts the CpG dinucleotide on the original molecule, thus preventing M.SssI-mediated methylation and resulting in loss of the CpG signal on both strands. Therefore, to confirm a CpG>TpG mutation, the double CpG correction algorithm requires two steps: a first correction to identify the presence of a T:A pair, and a second one to account for the C-to-T conversion on the opposite strand. (**C**) Detected CpG>TpG mutation frequencies in different contexts for two cell lines before (green) and after (orange) the second correction (*n* = 3 independent replicates per cell line). Centre lines indicate medians, box limits indicate the 25th and 75th percentiles, and whiskers extend to the smallest and largest values within 1.5 × the interquartile range (IQR), respectively. Source data are available online for this figure.

To evaluate the accuracy of MASD-seq, we first examined various M.SssI concentrations and reaction times. The 1 h/8 U condition achieved a CpG methylation rate of 99.7%, with no further increase observed at longer reaction times or higher enzyme concentrations. Given that the incomplete TET2 oxidation rate is approximately 1%, the effective M.SssI conversion rate is already near-complete (Appendix Fig. S1). Therefore, we selected this condition for subsequent experiments. Then, we performed MASD-seq on clonally derived GM12878 and HEK293T cell lines, which were utilized to minimize somatic mutations. The analysis showed an average CpG methylation level of 98.5%, with approximately 0.2% of CpG sites unmethylated on both strands and 1.4% hemi-methylated on either the Watson or Crick strand (Fig. 2A). The read-pair resolved rate reached 94.7%, and after quality filtering and alignment, an average of 56.5% of raw read pairs were retained, of which 65.3% contained UMIs (Dataset EV1). To enhance correction capability, we implemented a double CpG correction algorithm that leverages information from two complementary methylated CpG sites. A genuine CpG>TpG mutation will result in the loss of CpG on both strands. In contrast, mis-conversion of 5mC to T by incomplete TET2 oxidation, hemi-methylation of M.SssI, or 5mC deamination to T affects only one CpG site. To confirm a CpG>TpG mutation, the double CpG correction algorithm requires not only a T:A pair (first correction), but also the conversion of C to T on the opposite strand (second correction, Fig. 2B). This algorithm reduced the CpG>TpG error rate by approximately 50% in both cell lines (Fig. 2C).

**Figure 3:**
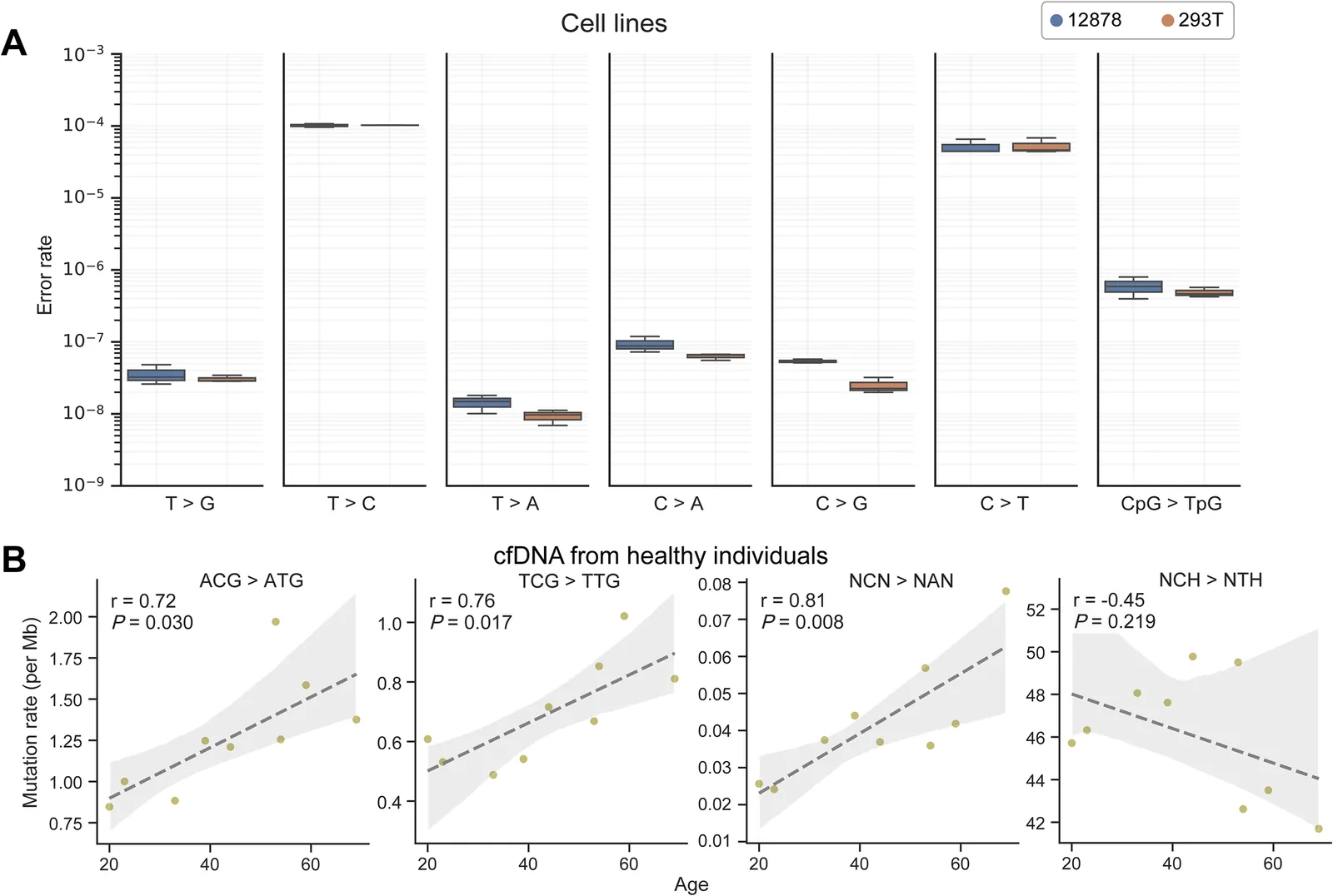
MASD-seq accuracy validation in cell lines and cfDNA. (**A**) Detected mutation rates for various mutation types in two cell lines. Centre lines indicate medians, box limits indicate the 25th and 75th percentiles, and whiskers extend to the smallest and largest values within 1.5 × the IQR, respectively. (**B**) Age-dependent mutation rate analysis in cfDNA samples from healthy volunteers (*n* = 9). Each dot represents a different individual, with dashed lines showing linear regression and gray shading indicating 95% confidence intervals. Associations were assessed using Pearson correlation tests. Source data are available online for this figure.

In two cell lines, MASD-seq achieved an overall CpG>TpG error rate of 5.4 × 10^−7^ (Fig. 3A). For the TCG, GCG, CCG, and ACG trinucleotide contexts, the error rates were 4.8 × 10^−7^, 3.0 × 10^−7^, 8.6 × 10^−7^, and 6.6 × 10^−7^, respectively (Appendix Fig. S2). For other mutation types, the error rates were 3.3 × 10^−8^ (T>G), 1.2 × 10^−8^ (T>A), 7.8 × 10^−8^ (C>A), and 3.9 × 10^−8^ (C>G), with an average of 4.1 × 10^−8^. As MASD-seq cannot correct CpH>TpH and T>C mutations, the error rates for both types remained at approximately 10^−4^ (Fig. 3A; Appendix S2). We also tested the GpC methyltransferase M.CviPI for correcting GpC>GpT errors, but its average methylation efficiency only reached 88%. Therefore, we used only M.SssI in subsequent experiments.

Next, we examined whether MASD-seq could detect physiological mutation burdens in human blood (Abascal et al, 2021; Osorio et al, 2018). We applied MASD-seq to plasma cfDNA from healthy volunteers (*n* = 9; Dataset EV1) across different ages (Fig. 3B; Appendix S3). The results revealed a clear positive correlation between age and mutation burden for correctable mutation types including ACG > ATG (r = 0.72, *P* = 0.03), TCG > TTG (r = 0.76, *P* = 0.017), NCN > NAN (r = 0.81, *P* = 0.008), NTN > NGN (r = 0.58, *P* = 0.098), and NAN > NTN (r = 0.51, *P* = 0.164). As expected, uncorrectable mutation types NCH > NTH and NTN > NCN showed no age-related trend (Fig. 3B; Appendix S3C). The CCG > CTG and GCG > GTG also did not exhibit an age-related trend, suggesting that their age-related mutation burden was below the detection limit of MASD-seq (Appendix Fig. S3C). In these healthy human cfDNA samples, the average error rates were 7.4 × 10^−8^ for T>G, 3.0 × 10^−8^ for T>A, 4.2 × 10^−8^ for C>A, 7.7 × 10^−8^ for C>G, and 9.4 × 10^−7^ for CpG>TpG. These results show that the high accuracy of MASD-seq enables the detection of age-associated increases in mutation burden in human blood.

We also compared bisulfite (BS) and EM-seq for MASD-seq library construction (see Methods). The results showed that the two methods exhibited comparable accuracy for detecting CpG>TpG and transversion mutations, as well as comparable methylation rates and CH conversion rates. However, BS resulted in higher DNA degradation and a higher duplication rate (Appendix Fig. S4, Dataset EV1).

### Mutation detection in CRC tissues

**Figure 4:**
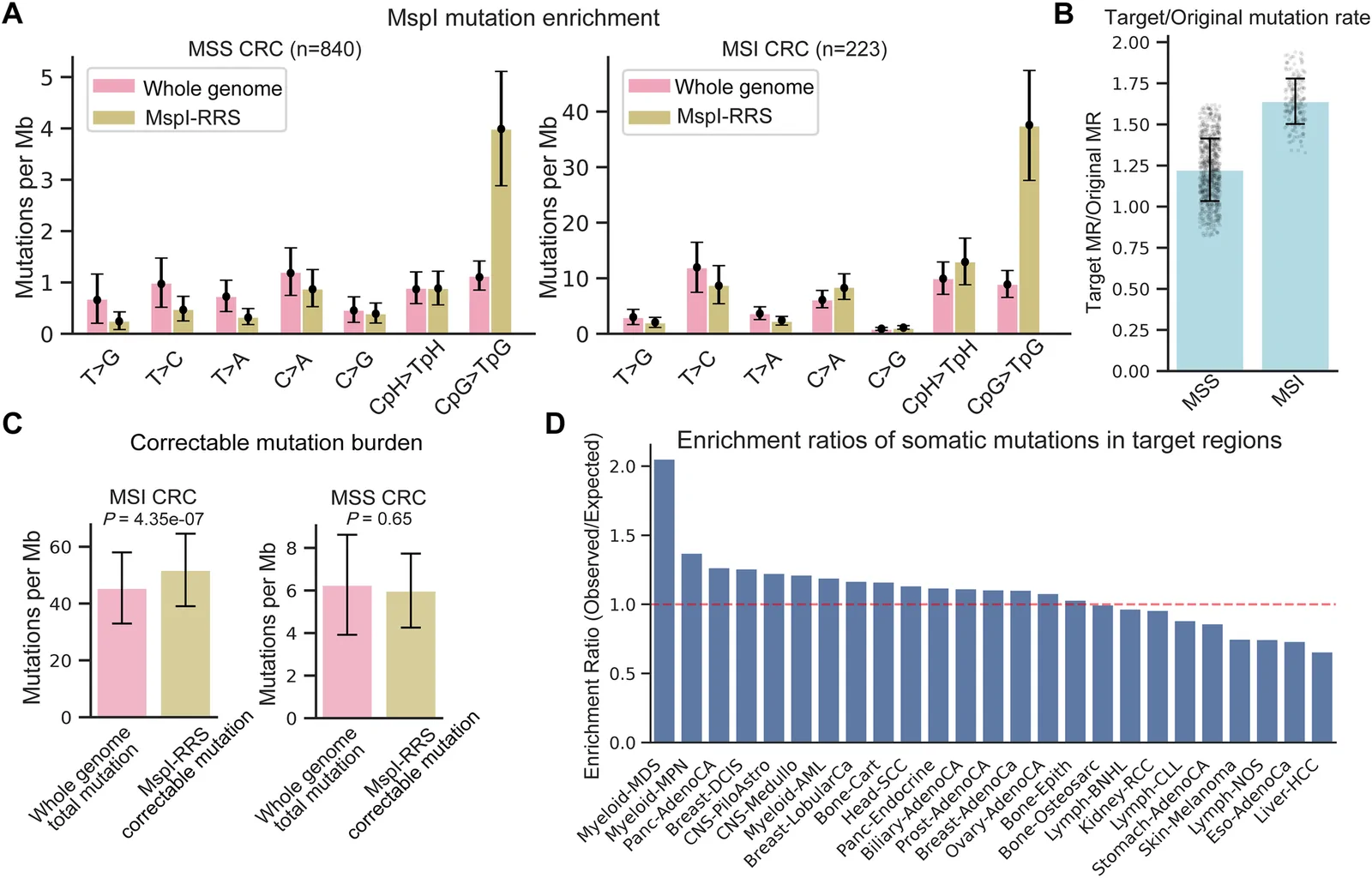
Enrichment of tumor mutation burden in the MspI-RRS region. (**A**) Comparison of mutation burdens between original genomic regions (pink) and MspI-RRS regions (yellow) in 50–200 bp fragments from MSS (*n* = 840) and MSI (*n* = 223) CRC samples, analyzed using the U-CAN cohort WGS dataset. To calculate the mutation burden in the MspI-RRS regions, the MspI recognition sequences were excluded from the analysis because their mutation prevents enzymatic cleavage. (**B**) Total mutation burden in the MspI-RRS regions relative to that in the whole genome in MSS (*n* = 840) and MSI (*n* = 223) CRC tumors; gray dots represent individual tumors. (**C**) Comparison of genomic mutations (pink) and correctable mutations identified via MspI-RRS (yellow) in MSI (*n* = 223) and MSS (*n* = 840) CRC samples. *P* values were calculated using Mann–Whitney tests. In (**A**–**C**), bars indicate means and error bars indicate SD. (**D**) Enrichment ratio of the MspI-RRS region for total mutation burdens across tumor types. The ratio was calculated as the normalized mutation burden in the MspI-RRS region divided by that in the whole genome. A ratio of 1 is indicated by a dashed line. Data were derived from WGS of the ICGC database. Source data are available online for this figure.

The CpG>TpG mutations are prevalent in human cancers, representing a hallmark of the mutational signature SBS1 (Alexandrov et al, 2020; Alexandrov et al, 2013; Cancer Genome Atlas Research, 2014; Cancer Genome Atlas Research et al, 2013; Cortes-Ciriano et al, 2017; Silveira et al, 2024). To examine the feasibility of using MspI-RRS to enrich for these mutations, we analyzed WGS data from the U-CAN cohort, which included 840 microsatellite stable (MSS) and 223 microsatellite instability (MSI) CRC tumor tissue samples (Nunes et al, 2024). We compared the tumor mutation burdens (TMB) of different mutation types between the whole genome and the MspI-RRS region. The MspI-RRS region, defined by fragments of 50–200 bp, collectively spanned approximately 58 Mb, representing about 2% of the human genome. Notably, the analysis demonstrated that the CpG>TpG mutation burden within this MspI-targeted region was 4.1-fold and 3.0-fold higher in MSI and MSS tumors, respectively, compared to the whole genome (Fig. 4A). In contrast, RRS regions generated with non-CpG-specific restriction enzymes (HindIII/BglII, HindIII/XbaI, and NcoI/StuI) and showed no mutation enrichment (Appendix Fig. S5A).

The total mutation burden in the MspI-targeted region was 1.6-fold higher in MSI tumors and 1.2-fold higher in MSS tumors than in the whole genome (Fig. 4B). Notably, after excluding uncorrectable mutations (CpH>TpH and T>C), the MspI-targeted region still retained significantly higher correctable mutation burden (CpG>TpG and transversions) than the total mutation burden (all transitions and transversions) in the whole genome in MSI samples (51.8 vs. 45.5 mutations/Mb, *P* < 0.001), while MSS samples showed similar mutation rates between them (6.0 vs. 6.3 mutations/Mb, *P* = 0.65; Fig. 4C).

We also examined data from the International Cancer Genome Consortium (ICGC) (Nunes et al, 2024). The results revealed a consistent enrichment of CpG>TpG mutations within the MspI-targeted region by 1.9- to 4.76-fold across multiple tumor types (Appendix Fig. S5B). The MspI-targeted region enriched the total mutation burden by 0.66- to 2.05-fold, with 16 out of 25 cancer types exhibiting a fold enrichment greater than 1 (Fig. 4D). When considering the correctable mutation burden, myelodysplastic syndromes (MDS), myeloproliferative neoplasms (MPN), pancreatic adenocarcinoma, and medulloblastoma exhibited an enrichment ratio higher than 0.9, with values of 1.32, 0.92, 0.97, and 0.92, respectively. Five cancer types including prostate adenocarcinoma, acute myeloid leukemia (AML), and breast invasive lobular carcinoma exhibited an enrichment ratio between 0.8 and 0.9 (Appendix Fig. S5B).

**Figure 5:**
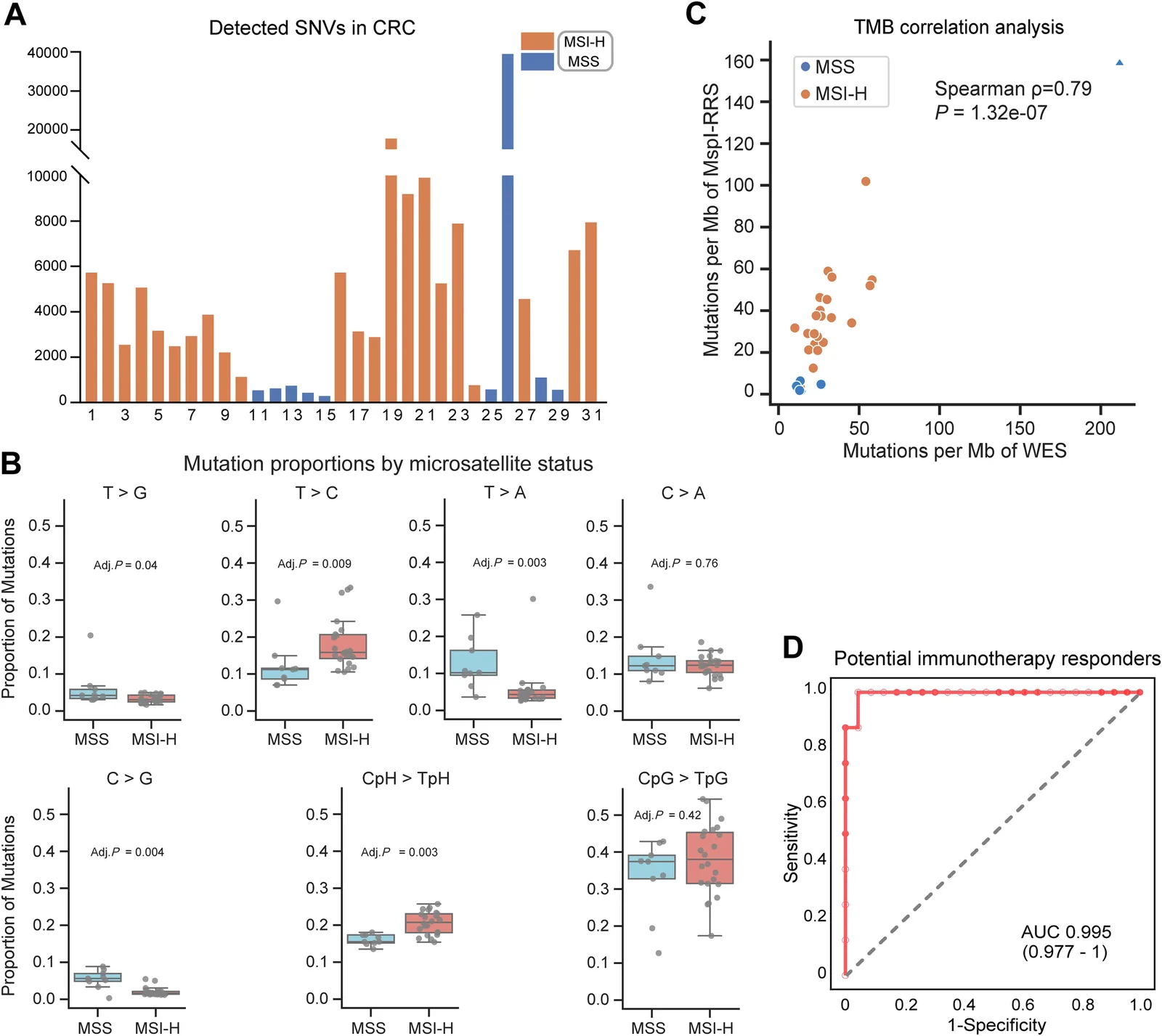
Mutation detection in CRC tissues. (**A**) Number of tumor-specific mutations across 31 CRC patients, stratified by stages and microsatellite status. (**B**) Proportion of mutation subtypes in 31 patients stratified by microsatellite status. FDR-adjusted *P* values are shown. *P* values were calculated using Mann–Whitney U tests and adjusted for multiple comparisons using the Benjamini–Hochberg procedure. Centre lines indicate medians, box limits indicate the 25th and 75th percentiles, and whiskers extend to the smallest and largest values within 1.5× the IQR, respectively. (**C**) Correlation analysis between TMB detected by MspI-RRS and WES in CRC patients. Patient 26 in the MSS group (triangle mark) exhibited an exceptionally high mutation count of 39,475, and further analysis identified this case as a clinically undiagnosed *POLE/POLD1* mutation patient with DNA replication fidelity defects (Haradhvala et al, 2018; Palles et al, 2013). (**D**) ROC curve analysis of MspI-RRS for identifying potential beneficiaries of immunotherapy, including patients with MSI-H and *POLE/POLD1* mutations. Source data are available online for this figure.

Next, we applied the MspI-RRS assay to analyze 31 CRC tissue samples, including 22 cases of high microsatellite instability/deficient mismatch repair (MSI-H/dMMR) and 9 cases of MSS/proficient mismatch repair (pMMR), with paired white blood cell (WBC) samples. The tumor tissue samples were sequenced for an average of 23 Gb raw data, and achieved an average sequencing depth of 51× for the MspI region. All cases were also subjected to WES for comparison. Detailed patient characteristics, sequencing information, depth and mutation counts are provided in Dataset EV2. The MspI-RRS assay identified a median of 4817 and 579 mutations for the MSI-H and MSS patients, respectively (Fig. 5A). Notably, CpG>TpG mutations accounted for a median of 39.5% and 37.6% of the total mutations in the MSI-H and MSS patients, respectively (Fig. 5B). The analysis showed that 98.1% (157,286 of 160,263) of the mutations were patient-specific, with 0.12% (187 of 160,263) shared among ≥3 patients.

Comparison of TMB detected by the MspI-RRS assay and WES demonstrated a strong correlation between the two methods (Spearman’s *ρ* = 0.79, *P* < 0.001; Fig. 5C). The TMB detected by the MspI-RRS assay showed an area under the curve (AUC) of 0.995 (95% CI: 0.977–1) for predicting immunotherapy candidates, demonstrating its value as a tool for identifying patients who may benefit from immunotherapy (Fig. 5D). Interestingly, TMB detected by MspI-RRS showed a stronger association with MSI-H status than that detected by WES (Appendix Fig. S5C). We further performed RRS assays using non-CpG-specific restriction enzymes (HindIII/BglII, HindIII/XbaI, and NcoI/StuI), and the results showed that the MspI-RRS assay markedly enriched tumor mutations compared to RRS assays using these enzymes (Appendix Fig. S5D).

To examine whether the MspI-RRS approach enriches for tumor mutations in other cancer types, we applied MspI-RRS to gastric cancer (GC; *n* = 7) and hepatocellular carcinoma (HCC; *n* = 5) samples, in comparison with HindIII/XbaI-RRS (Dataset EV3). The results showed that the MspI-targeted region consistently enriched CpG>TpG mutations compared with the HindIII/XbaI-targeted regions in both GC and HCC. The median CpG>TpG mutation burden increased from 0.056 to 0.716 mutations per Mb in GC and from 0.111 to 0.751 mutations per Mb in HCC (Appendix Fig. S6). In GC, although the total mutation burden was higher in the MspI-targeted region than in the HindIII/XbaI-targeted regions, the correctable mutation burden in the MspI-targeted region was lower than the total mutation burden in the HindIII/XbaI-targeted regions; however, neither difference was statistically significant. In HCC, the total mutation burden was comparable between the two regions, whereas the correctable mutation burden in the MspI-targeted region was markedly lower than the total mutation burden in the HindIII/XbaI-targeted regions.

Collectively, these results indicate that mutation-burden enrichment in the MspI-targeted region varies across human tumor types, with CRC being among the cancer types showing the highest enrichment ratios. Other cancer types, including pancreatic adenocarcinoma, prostate adenocarcinoma, and GC, may also be amenable to MspI-RRS-based enrichment for mutation detection.

### Performance of MASD-seq in blood

**Figure 6:**
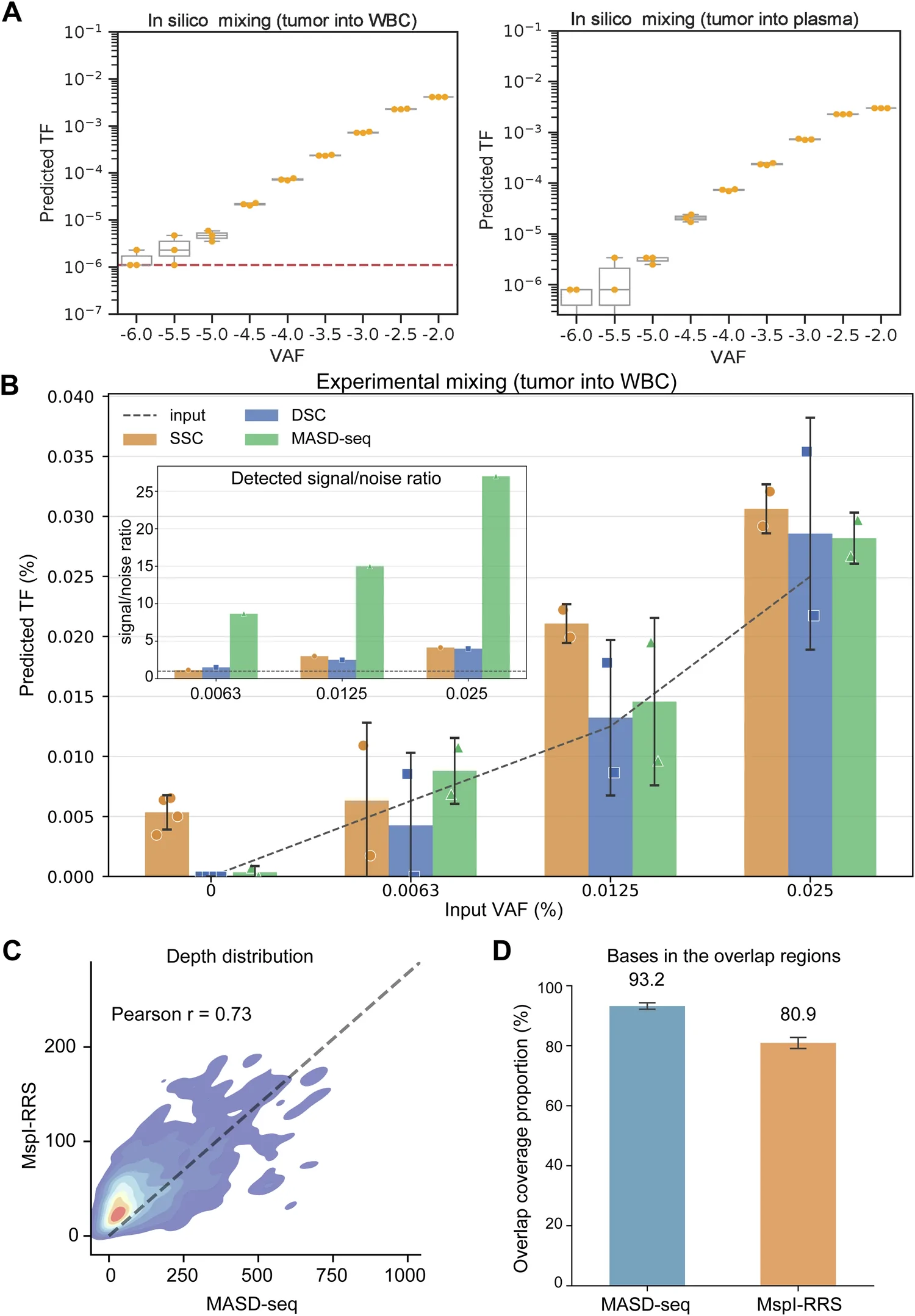
MASD-seq performance in blood sample analysis. (**A**) Assessment of detection limits using an in silico dilution series of tumor-derived reads into either WBC or control cfDNA backgrounds. The red dashed line indicates the baseline mutation fraction in WBC; this baseline is zero in control cfDNA and is therefore not shown. (**B**) Performance comparison of MASD-seq, single-strand consensus (SSC), and double-strand consensus (DSC) in detecting low-abundance mutant alleles (*n* = 2 replicate measurements per input VAF for each approach). Bars indicate means and error bars indicate SD. Tumor gDNA from Patient 1 was mixed into WBC gDNA from a healthy individual at expected VAFs of 0.025%, 0.0125%, 0.0063%, and 0%. MASD-seq was performed in comparison with a commercial hybrid-capture-based targeted sequencing assay that supports both SSC and DSC analysis. Main plot: Predicted TFs for the three approaches are shown, with the dashed line indicating the expected input VAF and bars showing mean values ± SD. Inset: Signal-to-noise ratios of detected mutation signals are shown, calculated using the 0% fraction as background. MASD-seq signals represent detected mutant sites, whereas SSC and DSC signals represent mutation-supporting reads (alt reads). (**C**) Correlation of sequencing depth between MASD-seq and MspI-RRS using 50–200 bp DNA fragments from paired cfDNA and tumor DNA, as exemplified by results from patient 3. (**D**) Comparison of target region coverage between MASD-seq and MspI-RRS using 50–200 bp DNA fragments from paired cfDNA and tumor DNA (*n* = 17 patients). Bars indicate means and error bars indicate SD. The left column shows the percentage of MASD-seq sequencing bases from plasma samples that overlap with the MspI-RRS-covered regions in matched tissue samples, while the right column presents the reciprocal analysis. Source data are available online for this figure.

To assess the capability of MASD-seq to detect ultra-low abundance mutations in blood, we applied MASD-seq to two MSI-H CRC tissues to evaluate its detection limit, using in silico mixing into background data from buffy coat and cfDNA of healthy volunteers to simulate a range of tumor fractions (TFs, which is the proportion of mutant alleles to the total number of alleles; see Methods). The results showed that MASD-seq reliably detected mutations with a linear relationship for spiked variant allele frequencies ranging from 10^−2^–10^−6^ in both backgrounds (Fig. 6A).

Next, we performed a spike-in experiment to compare MASD-seq with a commercial hybrid-capture-based targeted sequencing assay that supports both single-strand consensus (SSC) and double-strand consensus (DSC) analysis (Dataset EV4). The results showed that SSC exhibited a background of 0.53 × 10^−4^, which precluded clear detection of the 0.0063% fraction. In contrast, MASD-seq and DSC showed substantially lower backgrounds of 3.6 × 10^−6^ and 0, respectively, and both successfully detected the 0.0063% fraction (Fig. 6B). However, despite its high sensitivity, DSC had a relatively low recovery rate, detecting an average of only 3, 1.5, and 0.5 mutant alleles for the 0.025%, 0.0125% and 0.0063% fractions, respectively. By comparison, with matched raw data, MASD-seq detected an average of 39.5, 21.5, and 12 mutant alleles for the 0.025%, 0.0125%, and 0.0063% fractions, respectively (Appendix Fig. S7A). Consequently, MASD-seq achieved markedly higher signal-to-noise ratios than both DSC and SSC (Fig. 6B). A comparison between mutations derived from WGS and MspI-RRS showed that, despite the substantially lower raw sequencing data of MspI-RRS compared with WGS for the tumor tissue (19.2 Gb for MspI-RRS vs. 132 Gb for WGS), the two methods yielded similar signal-to-noise ratios and detected comparable tumor mutations at each dilution fraction (Appendix Fig. S7B).

We next applied MASD-seq to preoperative plasma cfDNA samples from 17 CRC patients, with matched tumor tissues analyzed by MspI-RRS (Datasets EV1 and 5). Comparison of the fragment distribution between the plasma cfDNA MASD-seq library and the tissue MspI-RRS library, as well as CpG methylation levels, is shown in Appendix Fig. S8. The analysis of paired tissue MspI-RRS and plasma MASD-seq sequencing results showed a strong positive correlation, with an average Pearson’s correlation coefficient (r) of 0.73 (range: 0.65–0.83; Fig. 6C). Across all samples, an average of 93.2% (range: 89.3–98.3%) of sequencing bases from MASD-seq fell within the region covered by MspI-RRS, demonstrating that MASD-seq achieved a high proportion of valid reads (Fig. 6D). Conversely, an average of 80.9% (range: 71.0–85.7%) of sequencing bases from MspI-RRS overlapped with the MASD-seq-covered region. The two methods also showed high concordance in tumor mutation detection (Appendix Fig. S9).

Across the plasma samples, the duplication level (read depth per unique read) decreased with increasing cfDNA inputs. At low input amounts (10–20 ng), we observed elevated levels (3–6×), whereas input above 30 ng achieved stable levels (1–2×) (Appendix Fig. S10). The average duplication level was 2.9 (range: 1.8–6) for CRC patients and 3 (range: 2.1–5.4) for healthy individuals (Dataset EV1).

### Perioperative ctDNA detection

**Figure 7:**
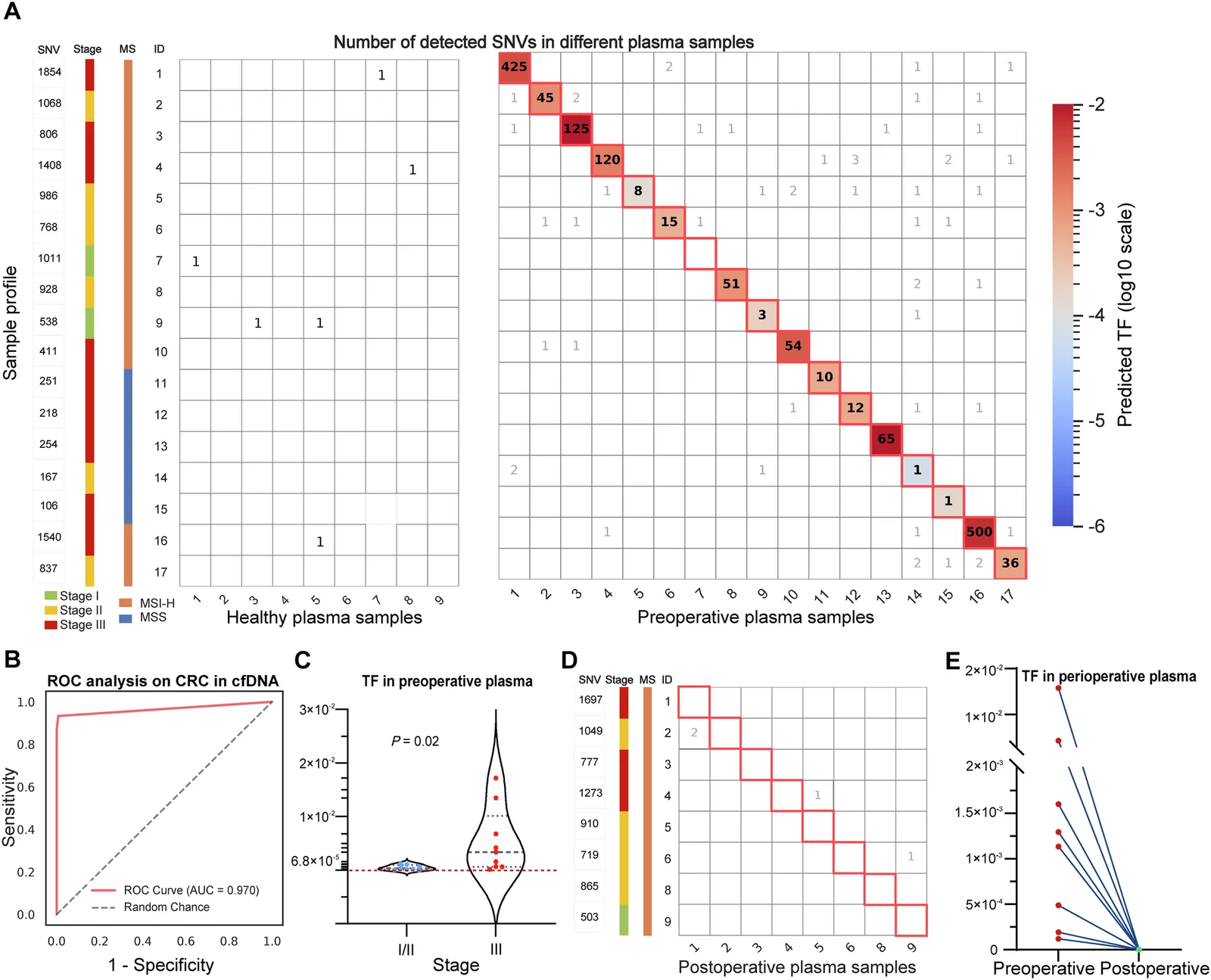
MASD-seq performance in perioperative detection. (**A**) Detection of SNVs in healthy plasma samples (left panel, *n* = 9) and preoperative plasma samples from CRC patients (right panel, *n* = 17). Sample identifiers are presented on the X-axis, with tissue sample identifiers on the Y-axis. Annotation of total number of SNVs, stage, and microsatellite status (MS) appears in the leftmost column. Tumor fraction (TF) in preoperative plasma samples is represented using different colors on a log10 scale. (**B**) ROC curve analysis for distinguishing cfDNA samples from preoperative CRC patients and healthy participants. (**C**) Estimated TF in stage I–II (*n* = 8) and stage III (*n* = 9) preoperative CRC patients. Each dot represents one patient. Violin outlines indicate kernel-density estimates, and internal lines indicate the 25th, 50th, and 75th percentiles. The *P* value was calculated using a two-sided Mann–Whitney U test. (**D**) Detection of SNVs in postoperative plasma samples from 8 MSI-H patients. (**E**) Comparison of TF between preoperative and postoperative plasma samples (mean post-surgery interval: 3 weeks). Source data are available online for this figure.

Next, we evaluated the performance of MASD-seq in detecting CRC. Among the 17 preoperative plasma samples analyzed, 12 were from MSI-H patients (two with stage I, five with stage II, and five with stage III) and five were from MSS patients (one with stage II and four with stage III). MASD-seq covered a median of 957 mutations (range: 411–1854) in the MSI-H group, compared to a median of 218 mutations (range: 106–254) in the MSS group. The median read depth was 138× (range: 85–199), with a median unique read depth of 51× (range: 48–109) (Datasets EV1 and 5). The assay successfully identified tumor-derived mutations in 16 of 17 cases (94.1% detection rate), with a median of 36 mutations per case (range: 0–500; Fig. 7A). One stage I patient (P7) showed a negative result. MSI-H cases revealed significantly higher median mutation counts (48; range: 3–500) than MSS cases (10; range: 1–65; *P* < 0.01), and stage III patients exhibited more mutations (median: 65; range: 1–500) than stage I–II patients (median: 12; range: 0–51; *P* = 0.047). In healthy controls, 96.1% (147/153) of cross-validation assessments were negative. The assay demonstrated an AUC of 0.970 for discriminating between CRC patients and healthy controls (Fig. 7B). Using a mutation count cutoff of ≥2, the assay achieved 100% (153/153) specificity in the healthy controls, with a sensitivity of 91.7% (11/12) in the MSI-H patients and 60% (3/5) in the MSS patients. Using a cutoff of ≥1, sensitivity was 91.7% (11/12) in the MSI-H patients and 100% (5/5) in the MSS patients, while specificity in the healthy controls was 96.1% (147/153).

Further analysis revealed that the estimated TF in patients’ preoperative plasma was significantly lower in stage I/II patients compared to stage III patients (median 4.9 × 10^−4^ vs. 5.4 × 10^−3^, *P* = 0.02; Fig. 7C). The cfDNA fragment length ranged from 40 to 138 bp. When stratified by fragment length, shorter fragments (< 100 bp) showed mutation enrichment, outperforming longer fragments (>135 bp) in both SNV (single nucleotide variant) detection (on average 34.1 vs. 26.0 for the short vs. long fragments, *P* = 0.021) and TF detection (on average 3.4 × 10^−3^ vs. 2.1 × 10^−3^ for the short vs. long fragments, *P* = 3.2 × 10^−3^; Appendix Fig. S11).

We next analyzed postoperative plasma samples from 8 MSI-H patients (Datasets EV1 and 5). Notably, ctDNA signals detected in all preoperative samples were no longer detectable postoperatively (Fig. 7D). Preoperative TF in blood ranged from 0.012 to 1.6%, and all declined to undetectable levels after surgery (Fig. 7E). These results also validated the high specificity of MASD-seq in ctDNA detection. Over a median follow-up period of 24 months, none of the patients with undetectable postoperative ctDNA experienced clinical recurrence.

Collectively, these results indicate that MASD-seq can detect ctDNA with high analytical specificity and sensitivity, particularly in MSI-H CRC patients.

## Discussion

In this study, we developed MASD-seq, an accurate sequencing method based on duplex consensus correction. MASD-seq enables error correction using single read pairs via physically linking the original Watson and Crick duplex strands. By employing CpG methyltransferase treatment and MspI digestion, MASD-seq effectively enriched tumor mutations and guarantees high duplex accuracy. We also developed an MspI-RRS assay for cost-efficient mutation profiling for tumor tissue. We showed that our method detected ctDNA in clinical plasma samples from CRC patients with high sensitivity and specificity, particularly for MSI-H tumors.

Previous studies have shown that the recovery efficiency of standard duplex sequencing ranges from 1 to 10% (Abascal et al, 2021; Bae et al, 2023; Kennedy et al, 2014b; Newman et al, 2016). This low efficiency arises primarily from the randomness of amplification and sequencing, thus, a large number of sequencing reads are required to obtain a duplex consensus sequence (DCS) family for reconstructing the original Watson and Crick strands. Based on mathematical modeling and empirical data, Abascal et al and Kennedy et al showed that the highest efficiency was achieved at a sequencing depth of 5, with a maximum recovery rate of 12%, meaning approximately 40 raw reads were needed to generate one DCS (Abascal et al, 2021; Kennedy et al, 2014b). Bae et al also observed that DCS formation did not start until 600 reads per target were obtained (Bae et al, 2023). In our experiments, the average DCS recovery rate was 2.5%. By physically linking the original Watson and Crick strands, MASD-seq enables the identification of DCS in a single-read pair, which allows DCS recovery from as few as one read and improves the efficiency by more than one order of magnitude.

Standard NGS is subject to 10^−2^ to 10^−4^ substitution errors per nucleotide (Gore et al, 2011; He et al, 2010; Ma et al, 2019; Salk et al, 2018). By applying single-stranded tagging approaches, the error rate can be reduced to the level of 10^−5^ (Bae et al, 2023; Deveson et al, 2021; Kinde et al, 2011; Newman et al, 2016; Rose Brannon et al, 2021; Salk et al, 2018). DNA damage, such as cytosine deamination and guanine oxidation, is the main error source for the single-stranded tagging approach (Chen et al, 2014; Schmitt et al, 2012). Experiments using uracil-DNA glycosylase (UDG) to excise uracil have shown that cytosine deamination (C>U) is a predominant artifact (Chen et al, 2014). The deamination rate of methylcytosine (5mC>T) is several-folds higher than that of cytosine, which cannot be eliminated by UDG and represents the highest error rates observed in NGS (Do et al, 2013; Frederico et al, 1990; Shen et al, 1994). Our results demonstrated that MASD-seq effectively corrected deamination of methylcytosine by using the complementary G base in the duplex consensus sequence, reaching an error rate as low as 5.4 × 10^−7^. Furthermore, MASD-seq enables correction of various transversion errors by duplex consensus, achieving error rates between 1.2 × 10^−8^ and 7.8 × 10^−8^.

We showed that this level of accuracy enables MASD-seq to detect age-related increases in mutation burden in blood. These results indicate that, for clinical ctDNA detection, the background is not dominated by sequencing errors, but rather by the age-related somatic mutation burden. Specifically, the background rates are 9.4 × 10^−7^ for CpG>TpG and 5.6 × 10^−8^ for transversions. Given that CpG>TpGs and transversion mutations account for approximately 60% and 40% of mutations, respectively, of all correctable mutations in the MspI digestion region in CRC, MASD-seq should yield an overall background error rate of approximately 6 × 10^−7^ for ctDNA detection. This error rate is more than two orders of magnitude lower than that of standard NGS and more than one order of magnitude lower than that of single-stranded tagging sequencing.

A disadvantage of the EM-seq- or bisulfite-based duplex sequencing approaches is their inability to correct all transition mutations, as the conversion of unmethylated C to T loses the original strand information (Mattox et al, 2017). In MASD-seq, the CpG methyltransferase M.SssI is utilized to methylate CpG sites to block their conversion, thereby enabling correction of CpG>TpG mutations through duplex consensus. We subsequently exploited the high frequency of CpG>TpG mutations in tumors to enrich these mutations. Specifically, we demonstrated that MspI digestion enriched CpG>TpG mutations by 4-fold and 3-fold in MSI-H and MSS CRC tumors, respectively. The total mutations were enriched by 1.6-fold and 1.2-fold in MSI-H and MSS CRC tumors, respectively. Consequently, the correctable mutation burden (CpG>TpG and transversions) in the MspI-digested region was comparable to the total mutation burden (all transitions and transversions) across the whole genome in MSI-H and MSS CRC tumors, although it does not reflect the full mutation landscape. In addition to mutation enrichment, MspI digestion also helps ensure the accuracy of MASD-seq as a duplex sequencing method. Previous studies have shown that the end-repair step can artifactually convert single-strand DNA lesions into uncorrectable double-strand errors (Abascal et al, 2021; Xiong et al, 2022). Studies have also shown that restriction enzyme digestion followed by direct ligation avoids the need for the end-repair step and maintains duplex accuracy (Abascal et al, 2021; Ueda et al, 2022). Therefore, by combining CpG methyltransferase treatment and MspI digestion, MASD-seq ensures a sufficient number of tumor mutations and high duplex accuracy.

We demonstrated that MspI-RRS for tissue analysis was compatible with MASD-seq for cfDNA analysis. It should be noted that MASD-seq using MspI digestion for cfDNA analysis is also compatible with WGS for comprehensive tissue mutation analysis. An inherent limitation of RRS is that it does not capture mutations in driver genes. However, in cases where driver gene information is not necessary, RRS can be applied to reduce genome complexity and save sequencing costs. Using MspI-RRS, approximately 40 Gb of sequencing data per patient case (20 Gb each for tumor and WBC) is used to obtain approximately 50× depth for mutation identification. In contrast, WGS requires 200 Gb per case (100 Gb each for tumor and WBC) to reach approximately 30× depth. Thus, MspI-RRS offers more than 5-fold improvement in cost efficiency compared to WGS. Clinically, the diagnosis of MSI-H relies on immunohistochemistry and analysis of microsatellites, while the efficacy of immunotherapy primarily depends on high tumor mutation burden. Therefore, MspI-RRS is a feasible and cost-efficient strategy for profiling mutations for MSI-H tumor tissue samples.

Our results demonstrated that MASD-seq achieved high sensitivity and specificity in detecting ctDNA of MSI-H/dMMR cancer patients. The method showed an AUC of 0.970 in discriminating between preoperative plasma from CRC patients and healthy controls. Also, all of the postoperative plasma samples from MSI-H patients converted to negative, validating the specificity of the method. Recent studies show great promise for immune-checkpoint inhibitors (ICIs) in treating MSI-H tumors (Williams et al, 2024). The NICHE-2 trial reported a 67.6% pathological complete response in MSI-H colon cancer patients (Chalabi et al, 2024). More recently, the NCT04165772 trial demonstrated a 100% clinical complete response rate to ICIs among patients with early-stage dMMR rectal cancer and a 65% rate among those with non-rectal solid tumors (Cercek et al, 2025). MASD-seq can be applied to MRD monitoring in these patients receiving ICI therapy, which has become increasingly clinically important.

Our results showed that the CpG>TpG mutation burden and tumor mutation enrichment by MspI-RRS vary across cancer types. CRC ranks among the highest in both metrics. For other cancer types, we propose that MASD-seq is expected to achieve relatively high efficiency in types with a correctable mutation ratio higher than 0.9, such as pancreatic adenocarcinoma, which exhibits values of 3.3, 1.28, and 0.97 for the CpG>TpG enrichment, the total mutation burden enrichment, and the correctable mutation ratio, respectively. For cancer types with slightly lower enrichment metrics, such as prostate adenocarcinoma (CpG>TpG enrichment: 3.0; total mutation burden enrichment: 1.11; correctable mutation ratio: 0.83), MASD-seq would still be acceptable. Our results also showed that GC is more amenable than HCC to mutation enrichment by MspI-RRS. Additional GC data are warranted to further evaluate this finding, given intertumor heterogeneity and potential population-specific differences in mutational signatures. Due to the limitation of uncorrectable mutations, the approach is less suitable for tumors with low CpG>TpG or high CH >TH/T>C burdens, such as liver cancer, kidney cancer, and melanoma. For lung cancer, although the CpG>TpG burden is low, an EM-seq-based hairpin strategy without MspI-digestion may still be applicable, as the mutational spectrum of lung cancer is dominated by C>A transversions, which are correctable by the strategy.

Several limitations of this study should be acknowledged. First, although we demonstrate analytical advantages of MASD-seq using a controlled spike-in model with a commercial duplex sequencing assay, these findings are derived from reference materials rather than from clinical patient-derived cfDNA samples. Second, the current experimental validation supports the clinical applicability of MASD-seq only in colorectal cancer, while its applicability to other tumor types remains to be validated. Third, the performance of MASD-seq could be further improved. In library construction, pre-amplifying the initial ligation product before EM-seq may enhance molecule recovery; moreover, applying DNA repair enzymes during cfDNA processing could increase the yield of duplex consensus DNA (Xiong et al, 2022). From a bioinformatics perspective, improving the mapping rate would further increase the number of detected molecules. Additionally, machine learning approaches may help further reduce the background error rate (Widman et al, 2024; Zviran et al, 2020).

MASD-seq can reveal biologically significant CpG>TpG and transversion mutations with high accuracy and high efficiency. By enriching CpG>TpG mutations, MASD-seq enables sensitive tumor mutation detection for ctDNA, and can be coupled with an MspI-RRS assay for cost-efficient mutation profiling in tumor tissue. These results indicate the potential of MASD-seq for future clinical applications, such as for MSI-H patients receiving ICI therapy.

## Methods

**Table:** Reagents and tools table

Reagent/Resource	Reference or Source	Identifier or Catalog Number
**Experimental models**		
GM12878	Tang Lab	NA
HEK293T	Tang Lab	NA
**Recombinant DNA**		
NA		
**Antibodies**		
NA		
**Oligonucleotides and other sequence-based reagents**		
Primers and adapters	This study	Methods and Appendix PDF
**Chemicals, Enzymes and other reagents**		
DNeasy Blood & Tissue Kit	QIAGEN	69506
VAHTS Free-Circulating DNA Maxi Kit	Vazyme	N903
Quick CIP	NEB	M0525
MspI	NEB	R0106M
CpG Methyltransferase M.SssI	NEB	M0226M
T4 DNA Ligase	NEB	M0202
NotI/NotI-HF	NEB	R3189/R3189S
Dynabeads MyOne Streptavidin C1	ThermoFisher	65002
NEBNext Enzymatic Methyl-seq Conversion Module	NEB	E7125
UvrD Helicase (20 µg/ml)	NEB	M1202S
Klenow Fragment (3’ → 5’ exo −)	NEB	M0212
Terminal Transferase	NEB	M0315
KAPA HiFi HotStart Uracil+ ReadyMix	Roche	KK2802
KAPA HiFi HotStart ReadyMix	Roche	KK2602
VAHTS Dual UMI Adapters	Vazyme	N351
AMPure XP Beads	Beckman Coulter	A63881
K2-EDTA blood collection tubes	BD	367525
ATP (100 mM)	ThermoFisher	R0441
**Software**		
NA		
**Other**		
Qubit 4 Fluorometer	ThermoFisher	Q33226
Illumina NovaSeq X Plus	Illumina	

### Participants and DNA samples

This study included clinically diagnosed CRC patients and healthy controls without malignant tumors. For the CRC patients, preoperative blood samples were collected after diagnosis, and postoperative samples were collected at their first follow-up appointment, with a median of 3 weeks after surgery. All participants were enrolled from the Department of General Surgery, and informed consent was obtained from each individual prior to their inclusion in the study. This study was approved by the Ethics Committee of Peking University Third Hospital (M2023676), and all experiments conformed to the principles set out in the WMA Declaration of Helsinki and the Department of Health and Human Services Belmont Report.

### Genomic DNA extraction

Cell lines, tumor tissues, and buffy coats were collected and stored at −80 °C until DNA extraction. Genomic DNA was extracted using the DNeasy Blood & Tissue Kit (QIAGEN, 69506).

### MspI-RRS library preparation

MspI-RRS libraries for both buffy coat and tumor DNA were prepared using a modified cfMethyl-Seq protocol with 50 ng of DNA input in a 25 µL total reaction volume. For 5’-end dephosphorylation, the mixture was incubated with 2 µL of Quick CIP (NEB, M0525) in 10× rCutSmart buffer at 37 °C for 30 min, followed by heat inactivation at 80 °C for 5 min. For 3’-end blocking, terminal transferase (NEB, M0315) and 1 mM ddGTP (Roche, 3732738001) were added, and the reaction was conducted at 37 °C for 2 h, followed by inactivation at 75 °C for 20 min. The mixture was then purified using 2× AMPure XP beads.

Restriction digestion was performed using 100 units of MspI (NEB, R0106), and the product was purified by dual size selection with 0.7× and 0.2× AMPure XP beads, followed by elution in 23 µL. End repair and A-overhang addition were conducted in a 30 µL reaction with Klenow exo- (NEB, M0212) and a dACGTP mixture, incubating at 30 °C for 20 min, 37 °C for 1 h, and 75 °C for 20 min. Subsequently, VAHTS Dual UMI Adapters (Vazyme, N351) were ligated using 2000 units of T4 Ligase (NEB, M0202) at 20 °C for 15 min, followed by purification with 1× AMPure XP beads. The final libraries were amplified by PCR using KAPA HiFi HotStart ReadyMix (Roche, KK2602) and UDI adapter primers. The PCR program was: 98 °C for 45 s; 13 cycles of [98 °C for 15 s, 60 °C for 30 s, 72 °C for 30 s]; and a final extension at 72 °C for 1 min. The PCR products were purified using 2× AMPure XP beads. Finally, libraries were sequenced on an Illumina NovaSeq X Plus platform using 150 bp paired-end reads, generating approximately 20 Gb of data per tumor tissue or WBC sample.

### Plasma cfDNA processing

Blood samples were collected using K_2_-EDTA tubes (BD, 367525) and plasma was separated within 6 h by two-step centrifugation at 1200 × *g* and 12,000 × *g*. cfDNA was extracted using the VAHTS Free-Circulating DNA Maxi Kit (Vazyme, N903). The concentration of the samples was quantified using a Qubit 4 Fluorometer (Thermo Fisher).

### MASD-seq library construction

cfDNA libraries were constructed using MASD-seq. In a 50 μL reaction, cfDNA was simultaneously treated with 20 units of CpG Methyltransferase (M.SssI, NEB, M0226M) and 100 units of MspI (NEB, R0106M) at 37 °C for 1 h to methylate CpG sites and digest MspI sites. The reaction products were purified using 3× AMPure XP cleanup. The purified cfDNA was then ligated with custom-designed biotin-labeled hairpin adapters and cytosine methylation-modified Y-adapters (Table EV1) using 2000 units of T4 Ligase (NEB, M0202). For a 20 ng cfDNA input, 8 ng of Hairpin and 4 ng of Y-adapters were used. The ligation proceeded at 20 °C for 1 h, followed by heat inactivation at 65 °C. As the adapters contain NotI recognition sequences, the reaction was subsequently incubated with 20 units of NotI (NEB, R3189) at 37 °C for 15 min to remove adapter-dimer products, followed by 3× AMPure XP cleanup.

To select for correctly ligated products, DNA was purified using Dynabeads MyOne Streptavidin C1 (Thermo Fisher, 65002) according to the manufacturer’s instructions. After purification, beads were eluted in 10 μL of 10 mM EDTA (pH 8.2) and 95% formamide at 65 °C for 5 min. The supernatant was transferred and purified with 3× AMPure XP. The cfDNA then underwent methylation conversion using an optimized protocol with the NEBNext Enzymatic Methyl-seq Conversion Module (NEB, E7125), where the initial denaturation step was omitted. During the cytosine deamination step, denaturation was achieved with UvrD Helicase. Specifically, a 70 μL reaction containing 1× APOBEC Reaction Buffer, 2.5 mM ATP, 5 μM MgCl_2_, APOBEC, BSA, and 50 ng of UvrD Helicase was incubated at 37 °C for 1.5 h, followed by bead purification. The final library was amplified with the KAPA HiFi HotStart Uracil+ ReadyMix Kit (Roche, KK2802) and UDI adapter primers. PCR conditions were: 98 °C for 1 min; 14 cycles (for 20 ng input) of [98 °C for 20 s, 65 °C for 30 s, 72 °C for 30 s]; and a final extension at 72 °C for 1 min. Final products were purified using 1.2× AMPure XP cleanup to complete the library. Each cfDNA sample was sequenced on an Illumina NovaSeq X Plus platform using 150 bp paired-end reads, generating approximately 100 Gb of data.

A detailed step-by-step protocol is provided in the Appendix PDF.

### Data processing of MASD-seq

Raw paired-end reads were quality-controlled with fastp (default parameters). To resolve hairpin structures, we performed a global alignment of read pairs using a customized couplet script with a gap penalty of −100. The resulting hairpin reads were processed with a custom tool to trim linkers and filter reads containing >3 internal mismatches. Reads were then trimmed by 8 bp (5’ end) and 4 bp (3’ end) and aligned to the GRCh38 reference genome using BWA-MEM. Only primary alignments with a mapping quality >30 were retained. We further filtered reads containing indels, soft-clipping, or >2 mismatches (NM tag). Base quality scores at hemi-methylated CpG sites were recalibrated using our hairpin.base_context_recalibrate function. Finally, reads in low-complexity regions or at positions in the gnomAD database were removed.

### Error-rate estimation

MASD-seq error rates were estimated from germline-filtered, high-confidence pileup records after sequential read-level filtering, base-level masking, genomic-region filtering, and trinucleotide-context aggregation. Raw paired-end reads were adapter- and low-quality trimmed and resolved into single hairpin consensus reads using the paired-end Hairpin overlap rule; read pairs were retained only when the mismatch rate during this resolution step did not exceed 5%. Linker-derived sequence was removed in tolerant mode (reads truncated before the linker when detected; terminal linker-derived segments removed when only the linker prefix was present; reads without detectable linker sequence retained). Reads were discarded if linker/UMI parsing was inconsistent, if the post-trimming read length was ≤20 bp, or if the resolved read contained >3N bases. For retained reads, the first 8 bases and last 4 bases were removed prior to alignment; a 4-base UMI tag was recorded when available.

Filtered resolved reads were aligned to the reference genome, retaining only primary alignments with mapping quality ≥30 and subsequently removing reads containing indels (CIGAR), soft clipping, or >2 mismatches (from the mismatch tag); thus pileup input comprised primary high-mapping-quality alignments without indels, without soft clipping, and with no more than two aligned mismatches. Pileups were generated with base-quality threshold ≥30 and mapping-quality threshold ≥60 on autosomes and sex chromosomes. Pileup sites were further restricted to predefined high-confidence callable regions excluding tandem-repeat and homopolymer-prone intervals, and sites overlapping known population variants from public resources were removed. For SNV error-rate estimation, sites whose pileup string contained insertion/deletion annotations were excluded. Potential germline variants were filtered using matched germline data: germline pileups were generated under the same quality thresholds, and each pileup site was considered germline-covered when its effective germline depth was ≥20; sites with any alternate base observed in the matched germline pileup were classified as potential germline variants and excluded. Consequently, both the numerator and denominator of the final error-rate estimates were calculated only from sites that were callable in the matched germline sample and had no germline alternate-base evidence.

For each retained site, pileup bases were parsed to define effective depth and alternate-base counts: non-alphabetic pileup symbols representing reference matches (e.g.,./,) remained in the depth denominator but did not contribute to alternate counts; alphabetic N and other non-A/C/G/T characters were removed from effective depth; alphabetic A/C/G/T characters were treated as alternate-base observations. Sites with zero effective depth after parsing were excluded. Context-specific error rates were then computed by conditioning on the reference trinucleotide centered on each retained pileup position: sites with trinucleotide contexts containing N or any non-A/C/G/T character were excluded, and the central reference base was required to match the pileup reference base. For each of the 64 possible trinucleotide contexts, the denominator was defined as the sum of effective pileup depths across all retained sites with that exact context, and alternate-base counts were accumulated separately for all alternate bases other than the central reference base (e.g., for context ACA with central reference C, channels ACA_A, ACA_G, ACA_T were aggregated, while ACA_C was excluded as the reference). The trinucleotide-specific error rate was calculated as: ${error\_rate}({context\_ALT})=\frac{\sum {{{\rm{alternate}}}}-{{{\rm{base}}}}\; {{{\rm{counts}}}}\; {{{\rm{for}}}}\; {{{\rm{context}}}}\_{{{\rm{ALT}}}}}{\sum {{{\rm{effective}}}}\; {{{\rm{pileup}}}}\; {{{\rm{depth}}}}\; {{{\rm{for}}}}\; {{{\rm{context}}}}}$

In the adjusted analysis, CpG- and GpC-associated conversion events were masked before pileup generation using base-modification flags: aligned C-to-T changes in CpG contexts (and reciprocal G-to-A changes in GpC contexts) were replaced by N and their base quality was set to zero when consistent with conversion rather than an ordinary sequencing mismatch; this masking was applied symmetrically to forward and reverse-complement representations. These masked bases were therefore excluded from both the numerator and denominator of the adjusted error-rate estimates.

### Calling somatic mutations

For data with Unique Molecular Identifiers (UMIs), barcodes were extracted (UMI-tools), reads were trimmed by 4 bp (cutadapt), and quality-filtered (fastp). After alignment (BWA-MEM), molecular consensus reads were generated and filtered using the fgbio toolkit (min base quality 30, max error rate 0.1).

For somatic variant calling, a dual-caller strategy (GATK Mutect2 and Strelka) was employed on aligned reads. Base quality scores were recalibrated using GATK BaseRecalibrator with standard resources (1000 Genomes, Mills indels, dbSNP138). Mutect2 was run with matched normals, a panel of normals, and a gnomAD resource; Strelka was run in somatic mode. The intersection of variants called by both tools was retained after filtering for artifacts in low-complexity regions and common population variants. Final variants were annotated with snpEff (GRCh38) and SnpSift (COSMIC). For all somatic mutation analyses of the MspI-digested region, we excluded the terminal CCGG recognition sites because mutations at these sites would prevent digestion and because they are routinely trimmed in MASD-seq data processing.

### Comparison of EM-seq and bisulfite conversion

We subjected 60 ng of GM12878 gDNA to MASD-seq library construction using either EM-seq or bisulfite conversion (BS), with replicates for each group. Bisulfite conversion was performed using the EpiArt Ultrafast DNA Methylation Bisulfite Kit (Vazyme, EM112-01). Briefly, 20 μL input DNA was mixed with 130 μl CT Conversion Reagent, incubated at 95 °C for 10 min, and then held at 20 °C. Converted DNA was purified on EpiArt DNA Columns, desulphonated at room temperature for 15 min, washed, dried by centrifugation, and eluted in nuclease-free water. Libraries from both treatment arms were sequenced and analyzed using the same MASD-seq pipeline, with each library sequenced to approximately 20 Gb. Library complexity, PCR duplication, CpG protection, strand-specific hemi-methylation, and background error profiles were compared between EM-seq and bisulfite-converted libraries.

### Detecting ctDNA fraction in MASD-seq cfDNA

We generated pileup data from filtered alignments using samtools mpileup (base quality ≥30, mapping quality ≥60). The pileup was then restricted to main chromosomes, excluding low-complexity regions and common variants from the gnomAD database.

Our custom hairpin SNV calling algorithm calculated position-specific error rates in a context-aware manner. For each position, we extracted its three-letter sequence context from the reference, excluding sites with ambiguous bases (N). CpG transitions (C>T, G>A) were processed separately, while A>G and T>C mutations were excluded from analysis due to high error rates. Each potential variant was annotated with its total depth (DP), alternative allele count (AC), and sequence context (CT).

We then used the union of all detected variant sites to estimate TF (tumor fraction) using MLE. Let f be the fraction of tumor sample DNA in the sample, ${a}_{i}$ denote the VAF of the i-th mutation in the original tumor sample. After accounting for sequencing errors, the expected probability of observing an alternative allele at site i in the test sample is:

$${{{{\rm{p}}}}}_{{{{\rm{i}}}}}={{{{\rm{fa}}}}}_{{{{\rm{i}}}}}+{{{\rm{\mu }}}}$$

The number of alternative reads at site i in the test sample was modeled as a Poisson-distributed random variable:

$${{{{\rm{Z}}}}}_{{{{\rm{i}}}}}\sim {{{\rm{Poisson}}}}({{{{\rm{D}}}}}_{{{{\rm{i}}}}}({{{{\rm{fa}}}}}_{{{{\rm{i}}}}}+{{{\rm{\mu }}}}))$$

Thus, the probability of observing ${z}_{i}$ alternative reads at site i is:

$${{{\rm{P}}}}({{{{\rm{Z}}}}}_{{{{\rm{i}}}}}={{{{\rm{z}}}}}_{{{{\rm{i}}}}}{{{\rm{|f}}}})=\frac{{\left[{{{{\rm{D}}}}}_{{{{\rm{i}}}}}\left({{{{\rm{fa}}}}}_{{{{\rm{i}}}}}+{{{\rm{\mu }}}}\right)\right]}^{{{{{\rm{z}}}}}_{{{{\rm{i}}}}}}\exp [-{{{{\rm{D}}}}}_{{{{\rm{i}}}}}({{{{\rm{fa}}}}}_{{{{\rm{i}}}}}+{{{\rm{\mu }}}})]}{{{{{\rm{z}}}}}_{{{{\rm{i}}}}}!}$$

Assuming conditional independence across sites, the joint likelihood is:

$${{{\rm{L}}}}({{{\rm{f}}}})={\prod }_{{{{\rm{i}}}}=1}^{{{{\rm{N}}}}}\frac{{\left[{{{{\rm{D}}}}}_{{{{\rm{i}}}}}\left({{{{\rm{fa}}}}}_{{{{\rm{i}}}}}+{{{\rm{\mu }}}}\right)\right]}^{{{{{\rm{z}}}}}_{{{{\rm{i}}}}}}\exp [-{{{{\rm{D}}}}}_{{{{\rm{i}}}}}({{{{\rm{fa}}}}}_{{{{\rm{i}}}}}+{{{\rm{\mu }}}})]}{{{{{\rm{Z}}}}}_{{{{\rm{i}}}}}!}$$

Taking the logarithm and removing terms that do not depend on f, we obtain the objective function used for estimation:

$${{{\rm{l}}}}({{{\rm{f}}}})={\sum }_{{{{\rm{i}}}}=1}^{{{{\rm{N}}}}}[{{{{\rm{Z}}}}}_{{{{\rm{i}}}}}\log ({{{{\rm{fa}}}}}_{{{{\rm{i}}}}}+{{{\rm{\mu }}}})-{{{{\rm{D}}}}}_{{{{\rm{i}}}}}({{{{\rm{fa}}}}}_{{{{\rm{i}}}}}+{{{\rm{\mu }}}})]+{{{\rm{C}}}}$$

Taking the derivative with respect to f and setting it to zero gives the MLE equation:

$${\sum }_{{{{\rm{i}}}}=1}^{{{{\rm{N}}}}}\frac{{{{{\rm{Z}}}}}_{{{{\rm{i}}}}}{{{{\rm{a}}}}}_{{{{\rm{i}}}}}}{{{{{\rm{fa}}}}}_{{{{\rm{i}}}}}+{{{\rm{\mu }}}}}={\sum }_{{{{\rm{i}}}}=1}^{{{{\rm{N}}}}}{{{{\rm{D}}}}}_{{{{\rm{i}}}}}{{{{\rm{a}}}}}_{{{{\rm{i}}}}}$$

Assume all tumor VAFs are identical, i.e., ${a}_{i}=a$, we can define the tumor-derived allele fraction as:

$${\sum}_{{{{\rm{i}}}}}\frac{{{{{\rm{Z}}}}}_{{{{\rm{i}}}}}{{{\rm{a}}}}}{{{{\rm{fa}}}}+{{{\rm{\mu }}}}}={\sum}_{{{{\rm{i}}}}}{{{{\rm{D}}}}}_{{{{\rm{i}}}}}{{{\rm{a}}}}$$

Thus, the estimated tumor-derived allele fraction is the observed aggregate alternative allele fraction corrected for the background sequencing error rate:

$${{{\rm{fa}}}}=\frac{{\sum }_{{{{\rm{i}}}}}{{{{\rm{Z}}}}}_{{{{\rm{i}}}}}}{{\sum }_{{{{\rm{i}}}}}{{{{\rm{D}}}}}_{{{{\rm{i}}}}}}-{{{\rm{\mu }}}}$$

### Germline bam processing

Raw paired-end reads were quality-controlled using fastp and aligned to the GRCh38 reference genome with BWA-MEM (-M flag). Alignments were filtered to retain only primary reads with a mapping quality ≥30. We then generated pileup data using samtools mpileup with stringent filters (base quality ≥30, mapping quality ≥60). Finally, potential germline variants were identified from the pileup using our hairpin tool, requiring a minimum depth of 15 (--include_depth) and a minimum alternative allele count of 1 (--minimal_alt_count).

### Detecting residual SNVs in MASD-seq cfDNA

The variant calling methods is the same as described above. To minimize contamination from germline variants, variant calling was performed on a germline-excluded pileup region where positions are filtered based on presence in regions with sufficient germline coverage and absence of germline variants at these positions.

### In silico mixing

We generated in silico mixed samples by computationally combining reads from tumor tissue with reads from one of two backgrounds: the patient’s white blood cells (WBCs) or cfDNA from healthy donors. This was done to simulate various tumor fractions (tumor_ratio) while avoiding artifacts associated with physical mixing at low concentrations.

At known tumor mutation sites, the number of tumor reads (S) to spike into a background of normal reads (normal_count) was calculated based on the tumor’s variant allele frequency (VAF) using the formula:

$${{{\rm{S}}}}={{{\rm{normal}}}}\_{{{\rm{count}}}}\times ({{{\rm{tumor}}}}\_{{{\rm{ratio}}}}/{{{\rm{VAF}}}})/(1-{{{\rm{tumor}}}}\_{{{\rm{ratio}}}}/{{{\rm{VAF}}}})$$

To ensure a robust background model, we used a pool of five WBC samples (including the patient’s own to account for germline SNPs) and a separate pool of cfDNA from seven healthy individuals.

### Spike-in experiment in comparison with SSC and DSC analysis

To evaluate the performance of MASD-seq relative to a hybrid-capture-based targeted sequencing assay, we performed a spike-in experiment using genomic DNA from Patient 1’s tumor tissue. This tumor gDNA was mixed into 60 ng of WBC gDNA from a normal individual at mutant allele fractions of 0.025%, 0.0125%, 0.0063%, and 0% (negative control), with replicates for each fraction. The hybrid-capture-based targeted sequencing assay used the iGenetech TargetSeq® Solid Tumor Mid Panel that covered 438.3 kb of solid-tumor-related genomic regions. For MASD-seq, each sample was sequenced to 100 Gb of raw data; for the hybrid-capture assay, each sample was sequenced to 30 Gb of raw data to achieve >20,000× depth for saturation. To enable a fair comparison, the MASD-seq data were downsampled to match the raw data input of the hybrid-capture assay. For both methods, tumor mutations were identified based on WGS data from the paired tumor and WBC samples of Patient 1, with 100 Gb of raw data generated per sample.

The iGenetech targeting assay incorporates 64 synthetic sequences as UMIs at the adapters, and thus enables both SSC and DSC analysis for ultrasensitive duplex sequencing. DSCs are generated following SSC construction by pairing reverse-complement UMI1/UMI2 combinations from opposite strands. For signal-to-noise analysis, the plotted value was the pseudocount-adjusted SNR, calculated as (signal mean+1)/(noise mean+1), where signal mean was the mean number of detected SNVs at each non-zero spike-in level and noise mean was estimated from the 0% fraction.

## Supplementary information

Appendix

Table EV1

Peer Review File

Dataset EV1

Dataset EV2

Dataset EV3

Dataset EV4

Dataset EV5

Source data Fig. 2

Source data Fig. 3

Source data Fig. 4

Source data Fig. 5

Source data Fig. 6

Source data Fig. 7

## Data Availability

A universal toolkit for analyzing MASD-seq data has been deposited on GitHub and is publicly available at https://github.com/yxwucq/Hairpin.git. The raw sequencing data have been deposited in the Genome Sequence Archive (GSA) for humans under accession number PRJCA044562 (CRC and control participants; https://ngdc.cncb.ac.cn/gsa-human/browse/HRA012753) and in the NCBI Sequence Read Archive (SRA) under accession number PRJNA1305108 (cell lines; https://dataview.ncbi.nlm.nih.gov/object/PRJNA1305108?reviewer=e29loq97l5rstsbd68mb8vcsna). Any additional data are available upon reasonable request. The source data of this paper are collected in the following database record: biostudies:S-SCDT-10_1038-S44321-026-00509-2.
