# Supplementary material for: Methyltransferase-assisted single duplex sequencing for detecting circulating tumor DNA
Source: EMBO Mol Med. 2026 Aug 19;18(10):3961–78. doi: 10.1038/s44321-026-00509-2 (PMC13658776; doi:10.1038/s44321-026-00509-2)
Supplement: Supplementary file 1 [file 44321_2026_509_MOESM1_ESM.pdf]

Table of Contents

Appendix Figure S1. Effect of different M.SssI conditions on MASD-seq performance. 1

Appendix Figure S2. Detected mutation rates in four contexts in two cell lines. 2

Appendix Figure S3. Detected mutation rates in cfDNA from healthy individuals. 3

Appendix Figure S4. Comparison of EM-seq and BS-seq in MASD-seq library construction. 4

Appendix Figure S5. Mutation detection efficiency of RRS. 7

Appendix Figure S6. Tumor mutation burden in the MspI region versus HindIII/XbaI region in GC and HCC. 8

Appendix Figure S7. Comparison of mutation detection between methods. 9

Appendix Figure S8. Fragment analysis and CpG methylation levels. 10

Appendix Figure S9. Comparative mutation detection across different sample types. 11

Appendix Figure S10. Relationship between relative read depth and cfDNA input amount. 12

Appendix Figure S11. Analysis of predicted tumor fraction and SNVs in short and long cfDNA fragments. 13

Appendix Supplementary Protocol: Detailed MASD-seq and MspI-RRS Library Construction 14

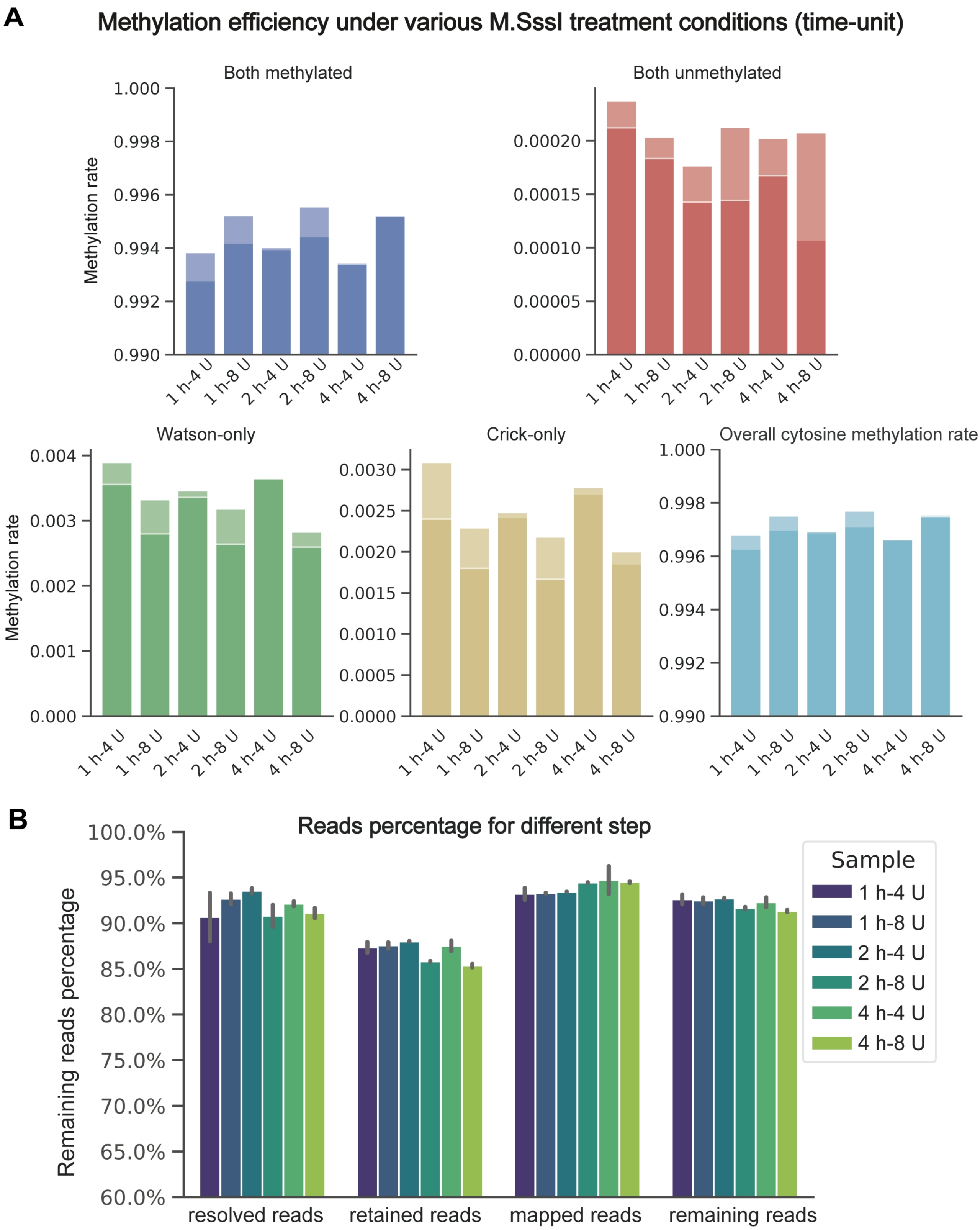

Appendix Figure S1. Effect of different M.SssI conditions on MASD-seq performance. A. Effect on methylation efficiency in PCR products. **B.** Percentage of reads retained across different bioinformatics filtering steps. 1 h-4 U: 1-hour incubation with 4 units of M.SssI.

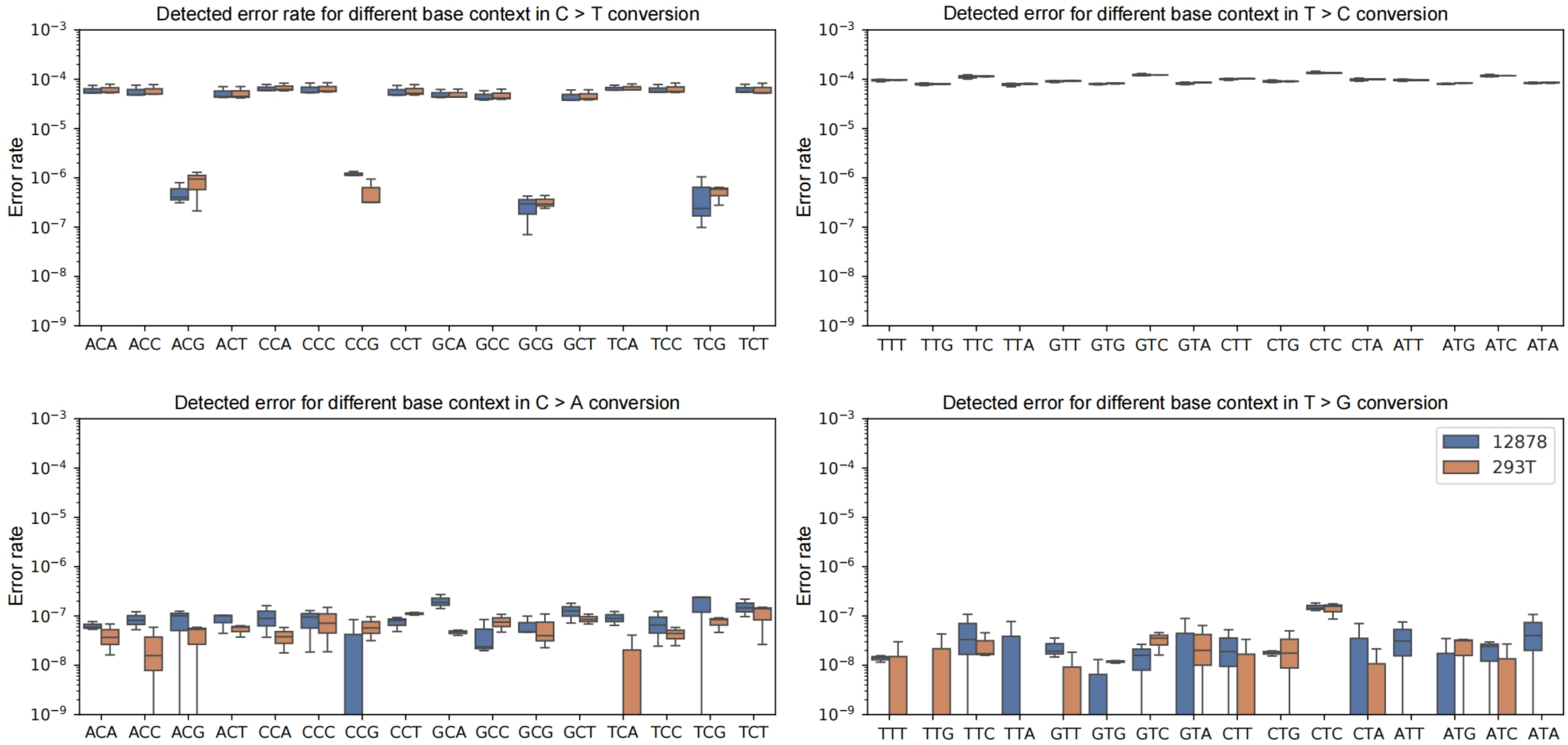

Appendix Figure S2. Detected mutation rates in four contexts in two cell lines. Error bars indicate standard deviation from three independent replicates.

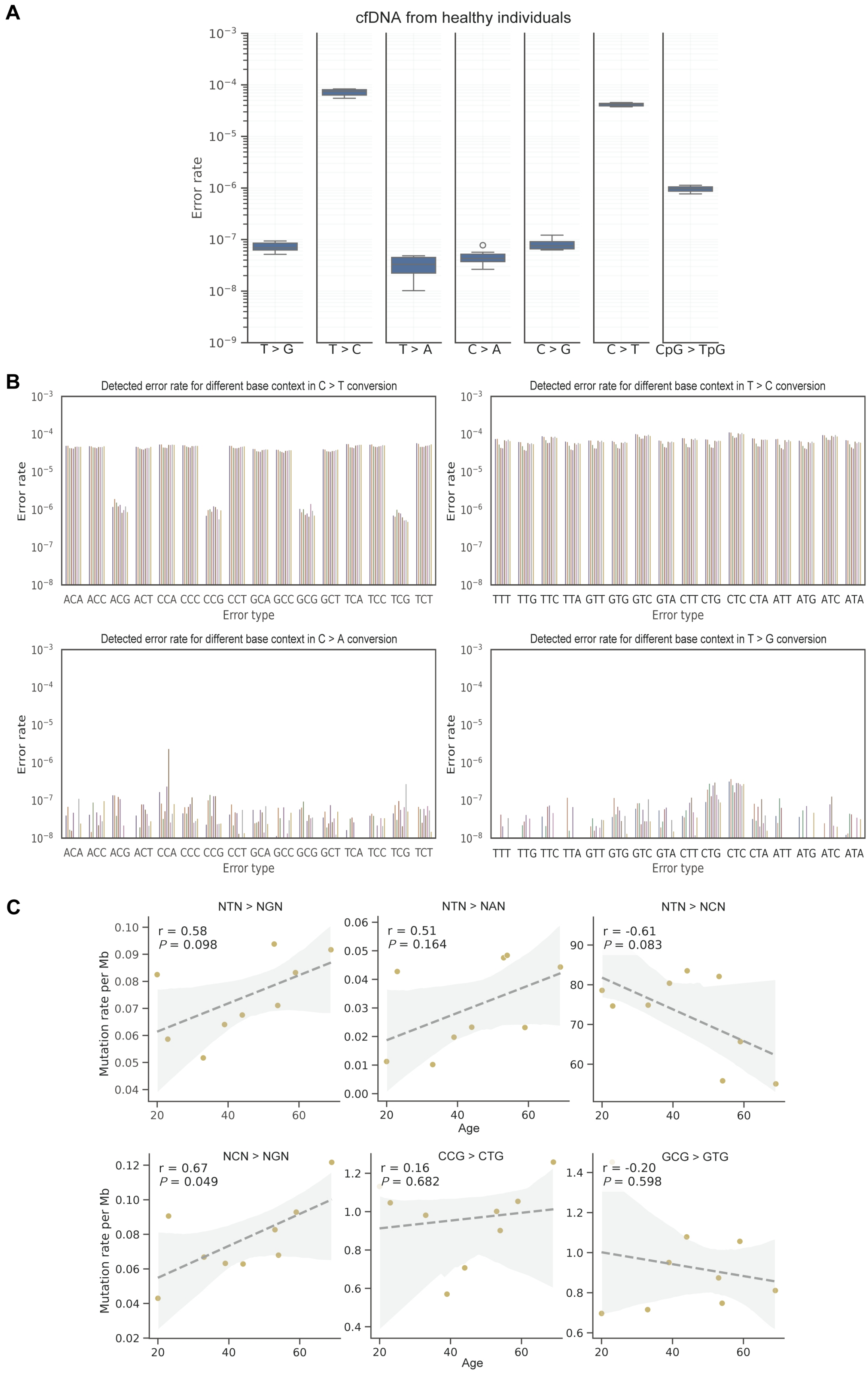

Appendix Figure S3. Detected mutation rates in cfDNA from healthy individuals. A. Error rates for various mutation types. **B.** Error rates in different base contexts. **C.** Age-dependent mutation rate analysis in cfDNA samples from healthy volunteers (n = 9). Each dot represents a different individual, with dashed lines showing linear regression and gray shading indicating 95% confidence intervals.

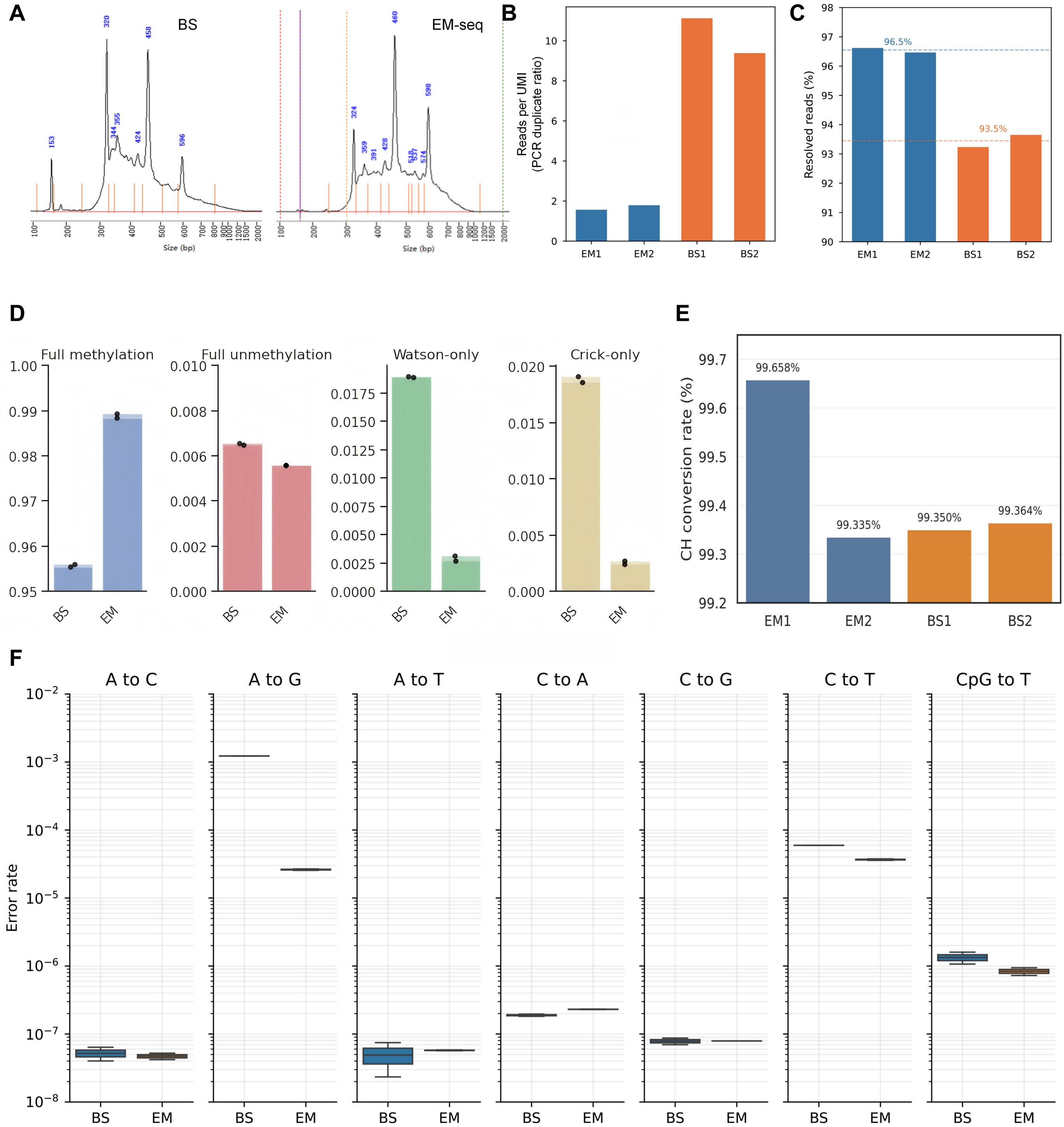

Appendix Figure S4. Comparison of EM-seq and BS-seq in MASD-seq library construction. MASD-seq libraries were prepared using either EM-seq or bisulfite (BS) conversion-based sequencing. **A.** Capillary electrophoresis fragment profiles, showing that PCR products of BS-based MASD-seq exhibited a shift toward shorter fragments. **B.** PCR duplication rate, shown as reads per unique molecule identifier (UMI). With approximately 20 Gb of raw sequencing data per library from 30 ng of starting gDNA, EM-seq exhibited an approximately 6-fold lower PCR duplication rate than BS (average of 1.7 reads per UMI for EM-seq versus 10.3 for BS). **C.** Hairpin-resolved molecule rate. EM-seq gave a higher resolve rate than BS (96.5% for EM-seq vs. 93.5% for BS), indicating its higher data quality. **D.** Methylation rates across different strand contexts. Both methods yielded high methylation levels, with EM-seq showing higher values than BS (98.9% for EM-seq vs. 95.6% for BS). The Watson-only unmethylation rate was 1.89% for BS vs. 0.29% for EM-seq, and the Crick-only unmethylation rate was 1.89% for BS vs. 0.26% for EM-seq; the double-strand unmethylation rate was 0.56% for EM-seq and 0.65% for BS. **E.** CH conversion rate, averaging 99.5% for EM-seq and 99.4% for BS. **F.** Error rates

across mutation types. The two methods showed comparable error rates for correctable error types, including A>C/T>G (6.8×10^{-8} for BS vs 6.2×10^{-8} for EM-seq), A>T/T>A (7.3×10^{-8} for BS vs 7.7×10^{-8} for EM-seq), C>A/G>T (2.6×10^{-7} for BS vs 3.3×10^{-7} for EM-seq), C>G/G>C (1.1×10^{-7} for BS vs 1.1×10^{-7} for EM-seq), as well as CpG>TpG (1.3×10^{-6} for BS vs 9.4×10^{-7} for EM-seq). Interestingly, BS treatment exhibited markedly higher A>G error rate compared with EM-seq (1.7×10^{-3} for BS vs 3.5×10^{-5} for EM-seq). Bars indicate replicate measurements where applicable.

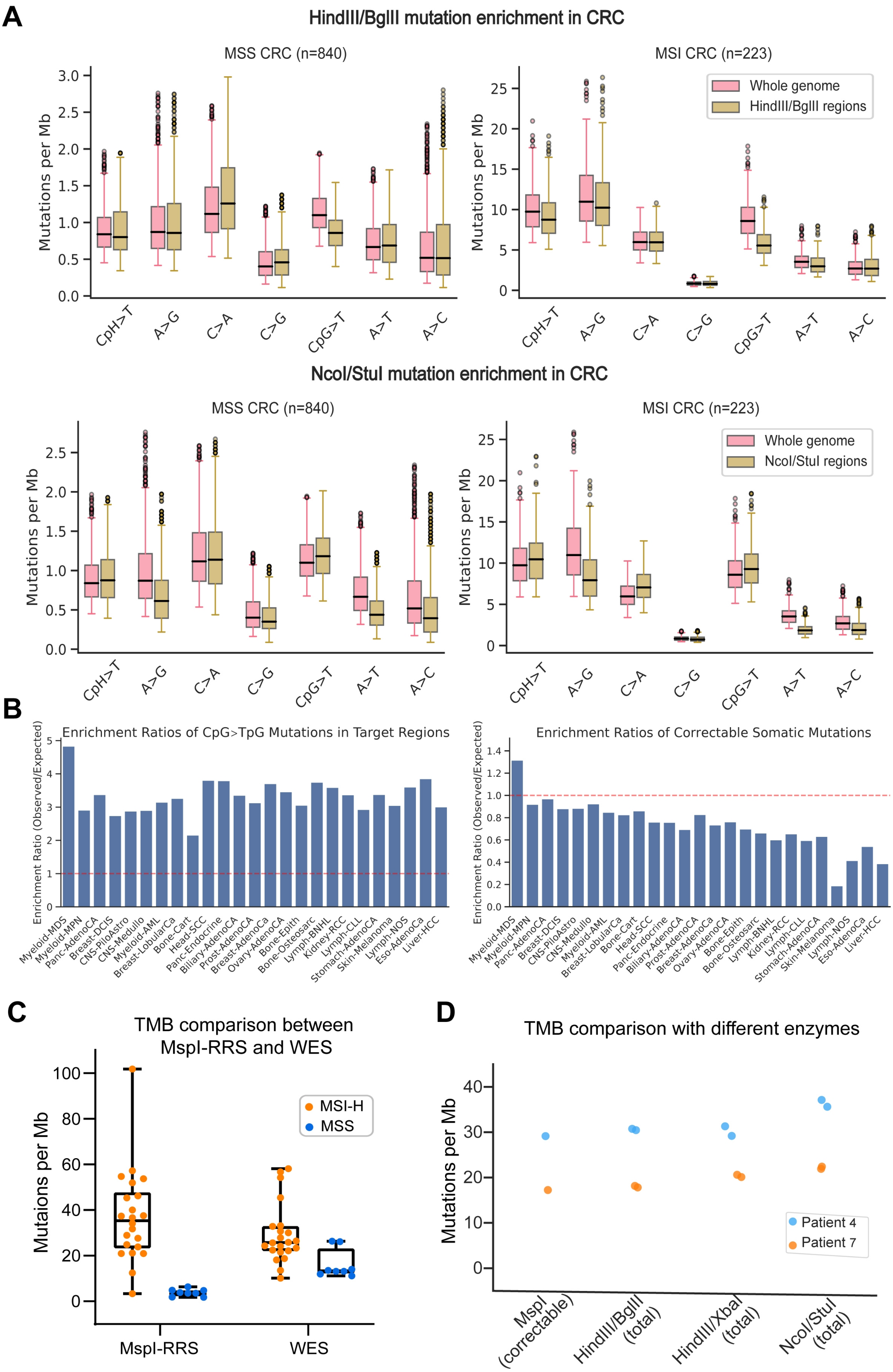
A
HindIII/BglII mutation enrichment in CRC
MSS CRC (n=840)
MSI CRC (n=223)
Mutations per Mb
Whole genome
HindIII/BglII regions
CpH>T
A>G
C>A
C>G
CpG>T
A>T
A>C
NcoI/StuI mutation enrichment in CRC
MSS CRC (n=840)
MSI CRC (n=223)
Whole genome
NcoI/StuI regions
B
Enrichment Ratios of CpG>TpG Mutations in Target Regions
Enrichment Ratios of Correctable Somatic Mutations
Enrichment Ratio (Observed/Expected)
Myeloid-MDS
Myeloid-MPN
Panc-AdenoCA
Breast-DCIS
CNS-PiloAstro
CNS-Medullo
Myeloid-AML
Breast-LobularCa
Bone-Cart
Head-SCC
Panc-Endocrine
Biliary-AdenoCA
Prost-AdenoCA
Breast-AdenoCa
Ovary-AdenoCA
Bone-Epith
Bone-Osteosarc
Lymph-BNHL
Kidney-RCC
Lymph-CLL
Stomach-AdenoCA
Skin-Melanoma
Lymph-NOS
Eso-AdenoCa
Liver-HCC
C
TMB comparison between MspI-RRS and WES
Mutaions per Mb
MSI-H
MSS
MspI-RRS
WES
D
TMB comparison with different enzymes
Mutations per Mb
Patient 4
Patient 7
MspI (correctable)
HindIII/BglII (total)
HindIII/XbaI (total)
NcoI/StuI (total)

Appendix Figure S5. Mutation detection efficiency of RRS. A. Mutation analysis using control restriction enzymes in CRC samples. Mutation burdens are shown for seven contexts in 50-200 bp fragments from the U-CAN cohort. Upper and lower panels represent HindIII/BglII and NcoI/StuI enzyme pairs, respectively. Each panel compares MSS (left) and MSI (right) samples, showing mutation burdens in original genomic DNA regions (pink) and enzyme-enriched regions (yellow). **B.** Enrichment ratios of different mutations in MspI-RRS target regions. Enrichment ratios were calculated as the normalized mutation burden in MspI-RRS regions divided by that in the whole genome. Left: Enrichment ratios of CpG>TpG mutations across tumor types. Right: Enrichment ratios of correctable somatic mutations across tumor types. A ratio of 1 is indicated by the dashed line. Data were derived from WGS analysis of the ICGC database. **C.** Comparison of TMB between MspI-RRS and WES in MSI-H (n=22) and MSS (n=8; P26 with *POLE/POLD1* mutation excluded) CRC samples. **D.** Experimental validation of mutation burdens across restriction enzymes using RRS in two CRC patients.

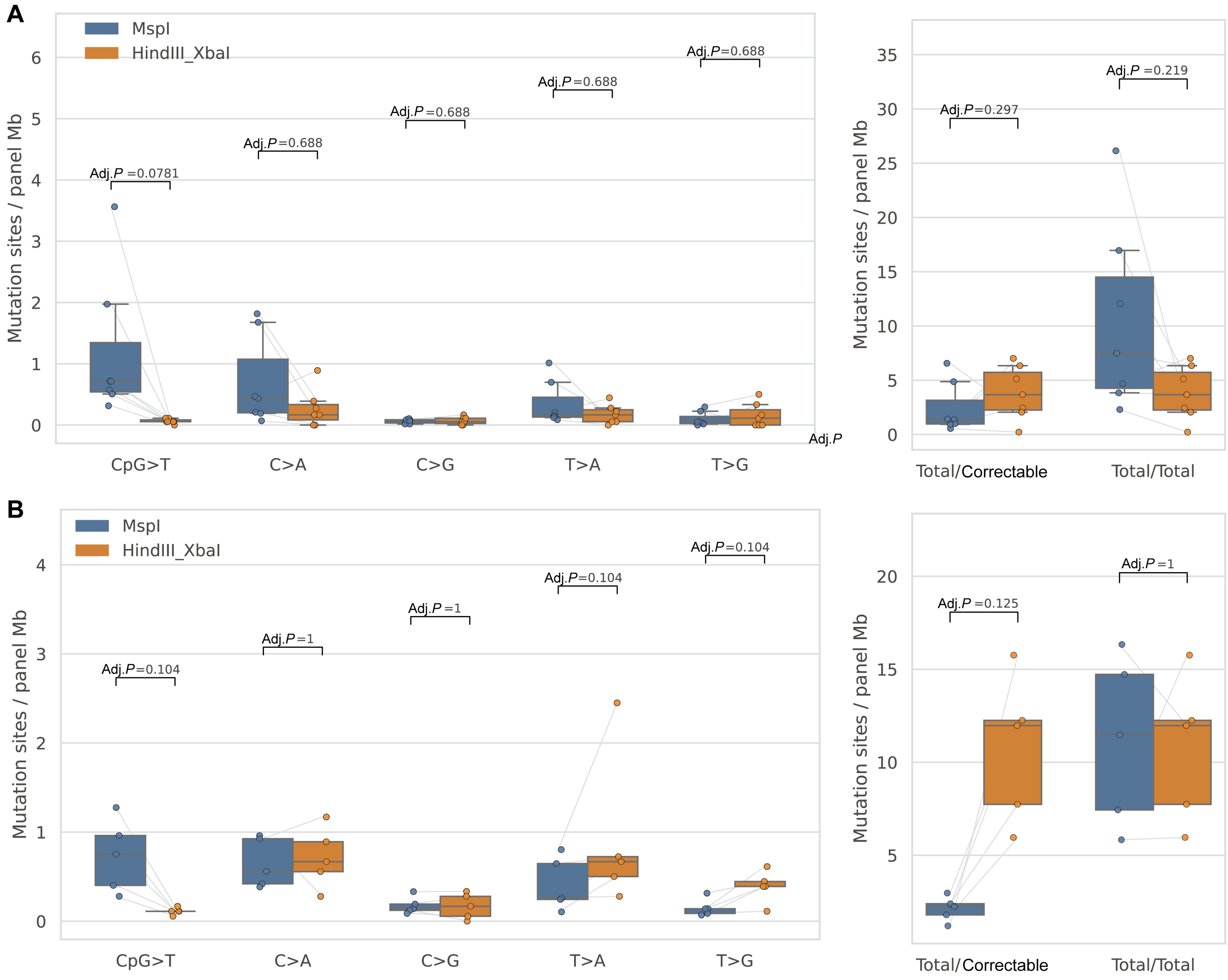

Appendix Figure S6. Tumor mutation burden in the MspI region versus HindIII/XbaI region in GC and HCC. A. Mutation burden comparison in GC. Left panel: distribution of different mutation classes. Right panel: comparison of corrected burden (MspI) versus total burden (HindIII/XbaI), and total mutation burdens between the two regions. **B.** Mutation burden comparison in HCC. Left panel: distribution of different mutation classes. Right panel: comparison of corrected burden (MspI) versus total burden (HindIII/XbaI), and total mutation burdens between the two regions. Bars indicate mean values, error bars indicate standard deviation, and dots represent individual samples.

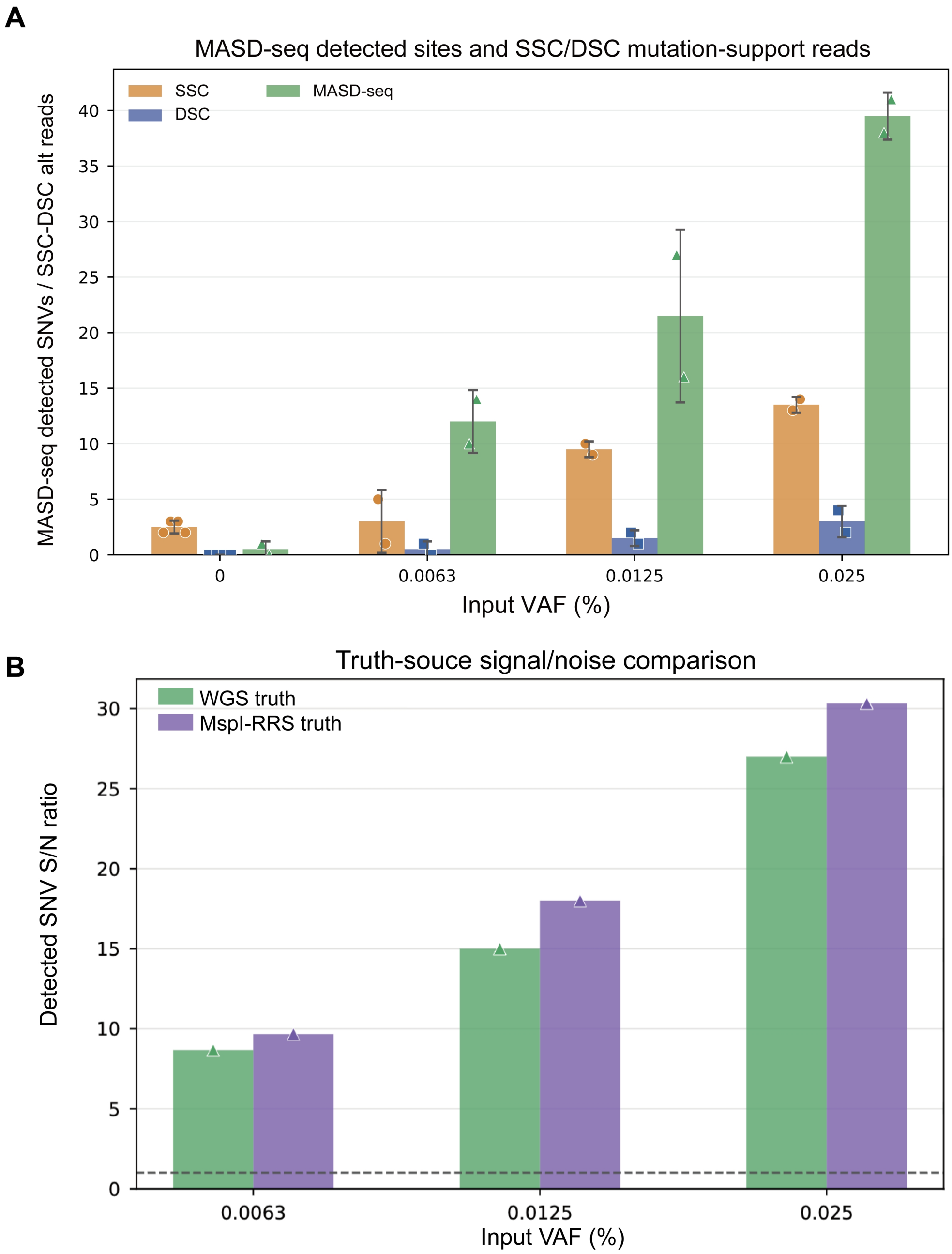

Appendix Figure S7. Comparison of mutation detection between methods. A. Detected mutation signals for the three approaches, generated by mixing tumor DNA from Patient 1 into WBC DNA at expected mutant allele fractions of 0.025%, 0.0125%, 0.0063%, and 0%. MASD-seq signals represent detected mutant sites, whereas SSC and DSC signals represent mutation-supporting reads (alt reads). MASD-seq, SSC and DSC detected an average of 161,303, 44,691, and 10,562 total read depth, respectively. **B.** The signal-to-noise ratios for SNV detection from WGS-derived and MspI-RRS-derived mutations are shown for each spike-in dilution fraction.

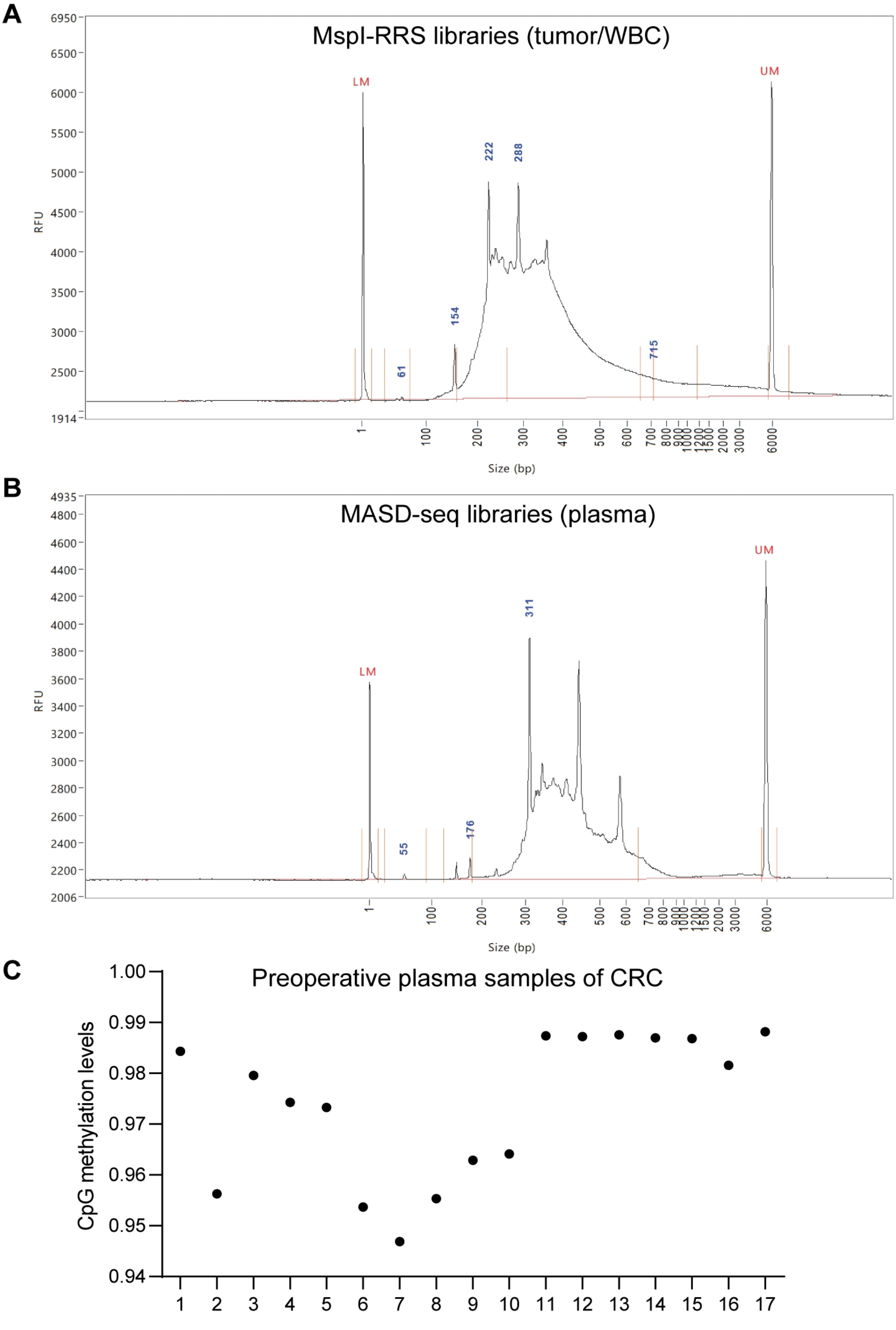

Appendix Figure S8. Fragment analysis and CpG methylation levels. A. Fragment distribution of library prepared with MspI-RRS from tumor tissue or paired buffy coat DNA. **B.** Fragment distribution of library prepared with MASD-seq from plasma cfDNA. **C.** CpG methylation levels in preoperative plasma samples from 17 CRC patients.

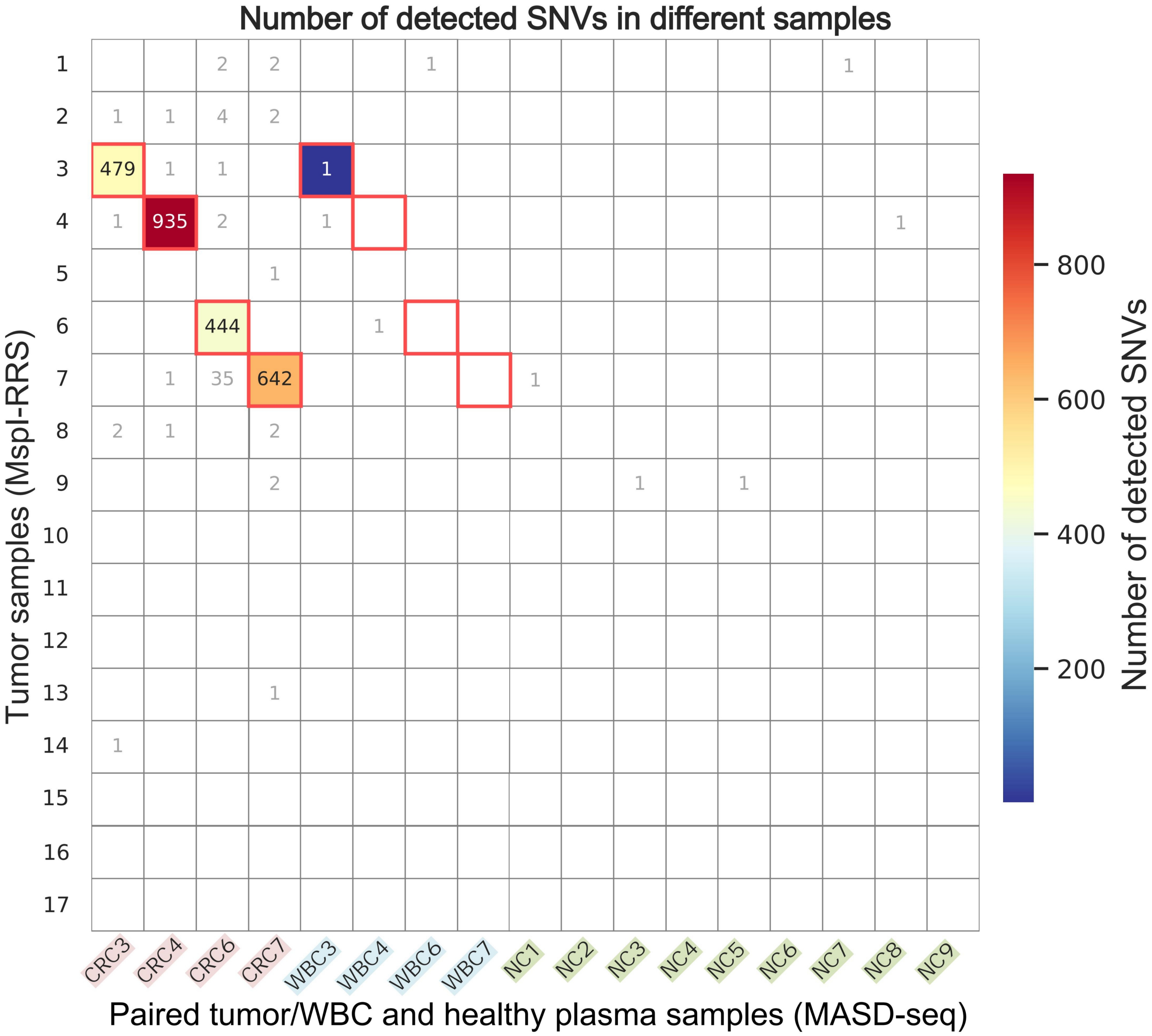

Appendix Figure S9. Comparative mutation detection across different sample types. MASD-seq was used to analyze SNVs in paired tumor samples (CRC), WBC samples, and healthy plasma samples (NC). Results were compared against MspI-RRS-detected mutations from 17 tumor samples. The X-axis displays different sample identifiers, while the Y-axis displays tumor tissue identifiers.

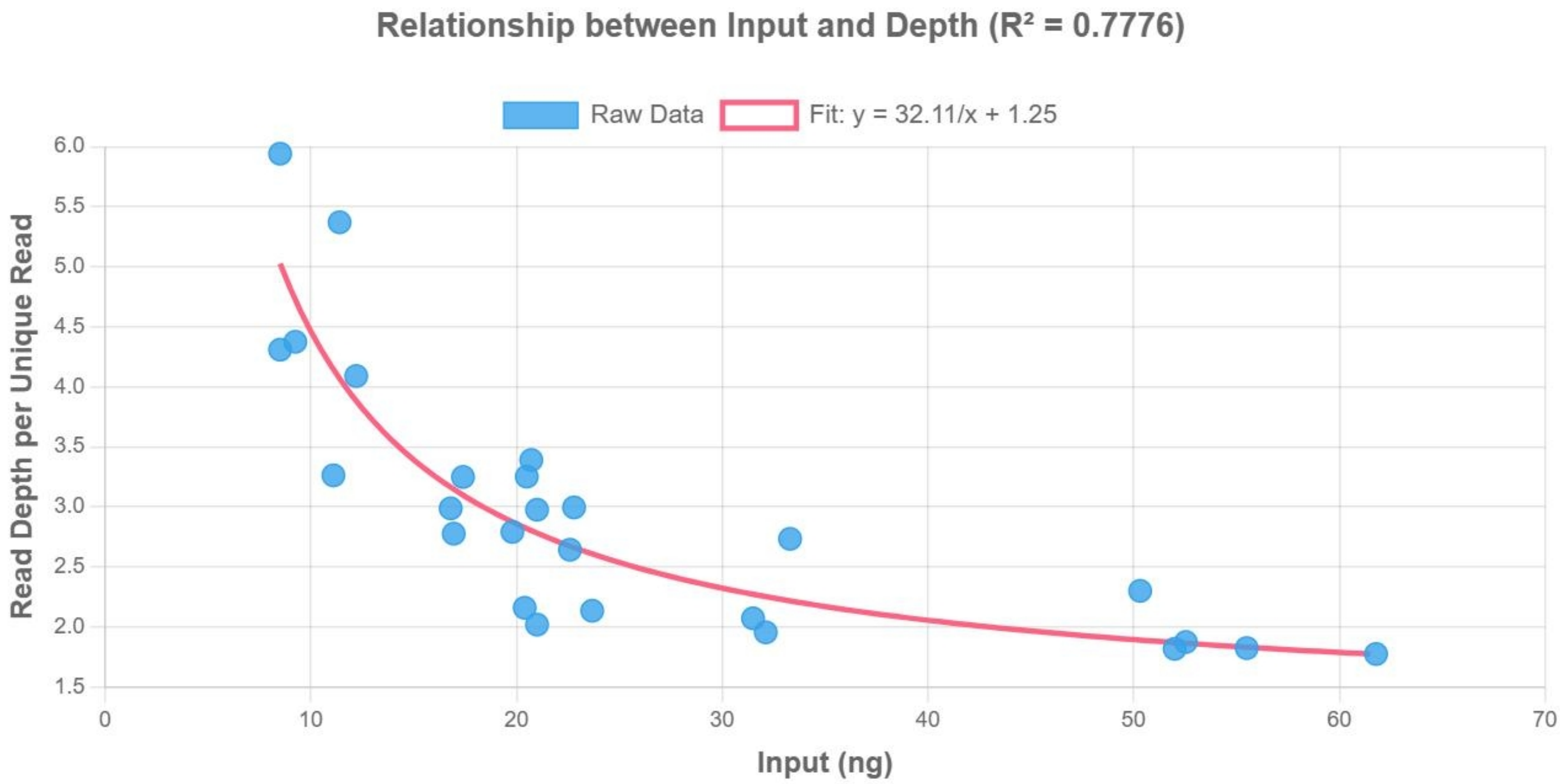

Appendix Figure S10. Relationship between relative read depth and cfDNA input amount. Relative read depth, defined as the depth at which 90% of reads are barcoded, is shown versus cfDNA input amount in 28 plasma samples from CRC patients and healthy individuals.

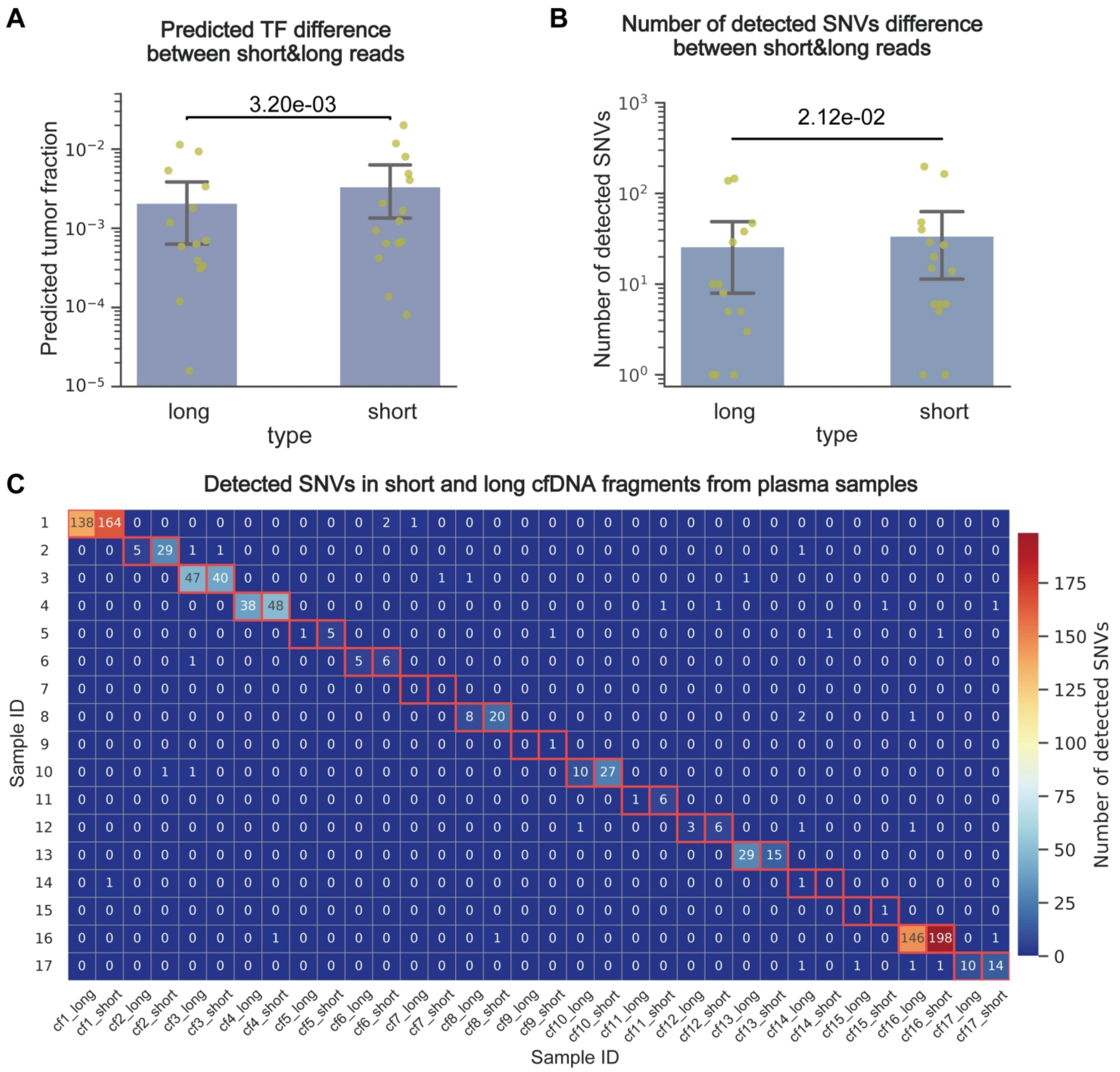

Appendix Figure S11. Analysis of predicted tumor fraction and SNVs in short and long cfDNA fragments. A. Predicted tumor fraction in short (< 100 bp) and long (> 135 bp) cfDNA fragments. **B.** Number of detected SNVs in short and long cfDNA fragments. **C.** Heatmap displaying detected SNV counts between paired short and long fragments and across different samples.

Appendix Supplementary Protocol: Detailed MASD-seq and MspI-RRS Library Construction

Overview

This supplementary protocol provides step-by-step instructions for the constructing MASD-seq libraries from cfDNA and MspI-RRS libraries from buffy coat and tumor tissue samples.

Part I: MASD-seq Library Construction (Plasma cfDNA)

Sample Collection and Plasma Processing

Materials:

- K2-EDTA blood collection tubes (BD, 367525)
- Nuclease-free water
- Qubit 4 Fluorometer (Thermo Fisher)

Procedure:

1. Collect blood samples in **K2-EDTA tubes (BD, 367525)**.
2. **Plasma separation** (perform within **6 hours** of blood collection):
   - **First centrifugation**: Centrifuge at **1,200 × g** for 10 minutes at 4°C to separate plasma from blood cells. Use slow acceleration/deceleration settings.
   - Transfer plasma carefully to a new tube without disturbing the cell pellet.
   - **Second centrifugation**: Centrifuge at **12,000 × g** for 10 minutes at 4°C to remove residual cells and platelets.
   - Transfer the clarified plasma to a new tube.
3. **cfDNA extraction** using VAHTS Free-Circulating DNA Maxi Kit (Vazyme, N903):
   - Follow the manufacturer's protocol for the sample volume processed.
   - Quantify extracted cfDNA using **Qubit 4 Fluorometer (Thermo Fisher)** with dsDNA HS Assay.
   - Store cfDNA at **−80°C** until library preparation.

1. M.SssI Methylation and MspI Digestion

Objective: Methylate all CpG sites by M.SssI to enable correction of CpG>TpG mutations after EM-seq, and simultaneously digest genomic DNA (gDNA) with MspI, which recognizes CCGG sites, to enrich CpG>TpG mutations.

Materials:

- cfDNA (Note: For optimal results, a minimum of 20 ng of DNA per reaction is required to achieve 50–100 Gb of sequencing data.)
- CpG Methyltransferase M.SssI (NEB, M0226M, 20 U/μL)
- MspI (NEB, R0106M, 100 U/μL)
- rCutsmart buffer (10×)

- S-Adenosyl methionine (SAM, 8 mM stock)
- Nuclease-free water

Procedure:

1. On ice, prepare a **50 µL** reaction mixture:

**Component**	**Volume (µL)**
cfDNA	42
rCutsmart buffer (10×)	5
SAM (8 mM)	1
MspI (100 U/µL)	1
M.SssI (20 U/µL)	1
**Total**	**50**

2. Mix thoroughly by pipetting; briefly centrifuge at 500 × *g* for 5 seconds.
3. Incubate in a PCR machine at **37°C for 1 hour**.
4. **DNA Purification**:
   - Add **150 µL** AMPure XP beads (3× volume).
   - Mix by pipetting 10 times.
   - Incubate at room temperature for **5 minutes**.
   - Place on magnetic rack for **1–2 minutes** until clear.
   - Remove supernatant; perform two 200 µL 70% ethanol washes (30 seconds each on magnetic rack).
   - Air-dry for **5–10 minutes**.
   - Elute in **41 µL** nuclease-free water; incubate at room temperature for 2 minutes, then place on rack and transfer eluate.
5. Keep at **4°C** or proceed immediately to Section 2.

2. Hairpin and Y-adapter Ligation

Objective: Ligate custom-designed biotin-labeled hairpin adapters and cytosine-methylated Y-adapters to cfDNA ends, thereby physically linking the Watson and Crick strands to enable single-duplex error correction.

Materials:

- Purified cfDNA (from Section 1, 41 µL)
- Hairpin adapter (annealed):
  - 5'-p-CGGCCGCAGTCDDDD/Int Biotin dT/DDDDGACTGCGGC-3'
  - Contains 5' phosphorylation, internal biotin dT modification, and randomized UMI segments (D = A/T/G combinations)
- Y-adapter (annealed duplexes):

- Primer A: 5'-ACACTCTTTCCCTACACGACGCTCTTCCGATCT-s-G-3'
- Primer B: 5'-p-CGCAGATCGGAAGAGCACACGTCTGAACTCCAGTC-3'
- **Critical**: All cytosine bases in the adapter sequences must be **methylation-modified** to ensure protection during downstream enzymatic methylation conversion.

- T4 DNA Ligase (NEB, M0202, 2000 U/μL)
- rCutsmart buffer (10×)
- ATP (100 mM stock)
- Nuclease-free water

Procedure:

1. On ice, prepare a **50 μL** ligation mixture:

**Component**	**Volume (μL)**
Purified cfDNA	40.5
rCutsmart buffer (10×)	5
ATP (100 mM)	0.5
Hairpin adapter (8 ng/μL stock)	1
Y-adapter (4 ng/μL stock)	1
T4 DNA Ligase (2000 U/μL)	2
**Total**	**50**

2. Mix thoroughly by pipetting; briefly centrifuge at 500 × *g* for 5 seconds.
3. Incubate at **20°C for 30 minutes**.
4. Heat inactivate at **65°C for 10 minutes.**
5. Cool to room temperature or place at **4°C**.
6. Keep at **4°C** or proceed immediately to Section 3.

3. NotI Digestion to Remove Adapter-Dimer Products

Objective: Remove non-specific ligation products for which direct ligation of the hairpin and the Y-adapter reconstitutes NotI recognition sites (5'-GCGGCCGC-3').

Materials:

- Ligation product (from Section 2, 50 μL)
- NotI (NEB, R3189, 20 U/μL)
- Nuclease-free water

Procedure:

1. After ligation, briefly centrifuge the reaction at 500 × *g* for 5 seconds.
2. Add **1 μL** NotI (20 units) directly to the **50 μL** ligation mixture.
3. Incubate at **37°C for 15 minutes**.

4. **DNA Purification**:
    - Add **150 µL** AMPure XP beads (3× volume).
    - Follow standard bead purification: mix, incubate 5 min at RT, rack 1–2 min, remove supernatant, two 200 µL 70% ethanol washes, air-dry 5–10 min.
    - Elute in **40 µL** nuclease-free water.
5. Keep at **4°C** or proceed immediately to Section 4.

4. Streptavidin Magnetic Bead Selection for Correctly Ligated Products

Objective: Selectively capture and purify biotin-labeled hairpin-adapter-containing DNA molecules that represent correctly ligated products (one hairpin + one Y-adapter per DNA molecule).

Materials:

- Purified DNA (from Section 3, 40 µL)
- Dynabeads MyOne Streptavidin C1 (ThermoFisher, 65002)
- Wash buffer (10 mM Tris-HCl pH 7.5, 1 mM EDTA, 2 M NaCl, 0.05% Tween 20 for 2× buffer; 1× prepared by dilution)
- Elution buffer: 95% formamide + 10 mM EDTA (pH 8.2)
- Nuclease-free water

Procedure:

1. **Prepare magnetic beads** according to the manufacturer's instructions:
    - Vortex Dynabeads thoroughly for 30+ seconds to resuspend.
    - Determine beads volume needed (typically 10 µL beads per DNA sample).
2. **Wash and prepare beads**:
    - Transfer beads to a new tube.
    - Add wash buffer and resuspend; place on magnetic rack for 1 minute; remove supernatant.
    - Repeat wash 3 times total.
    - Resuspend washed beads in 40 µL 2× buffer according to manufacturer's volume recommendations.
3. **Bind DNA to beads**:
    - Mix 40 µL biotin-labeled DNA with the resuspended beads.
    - Incubate with gentle rotation at room temperature for **15 minutes**.
4. **Wash beads**:
    - Place tube on magnetic rack for 2–3 minutes.
    - Remove and discard supernatant.
    - Add 200 µL 1× wash buffer; resuspend and mix.
    - Place on rack for 1 minute; remove supernatant.
    - Repeat wash 3 times total.
5. **Elute DNA from beads**:

- After final wash, add **20 µL** of elution buffer (95% formamide + 10 mM EDTA, pH 8.2).
- Incubate at **65°C for 5 minutes**.
- Briefly centrifuge.
- Place on magnetic rack for 1 minute.
- Transfer eluate (supernatant) to a new tube.

6. **Final purification**:
    - Add **60 µL** AMPure XP beads (3× volume).
    - Follow standard bead purification protocol.
    - Elute in **28 µL** nuclease-free water.
7. Keep at **4°C** or proceed immediately to Section 5.

5. Enzymatic Methylation Conversion: Oxidation and Deamination

Objective: Protect artificial 5-methylcytosine (from CpG methylation) and genomic 5mC via oxidation-based protection, while converting unprotected cytosines to uracil via APOBEC3A-mediated deamination. **Critically**: the initial Denaturation step of the standard kit is omitted; instead, denaturation is achieved using UvrD Helicase during the Deamination step.

Materials:

- Purified DNA (from Section 4, 28 µL)
- NEBNext Enzymatic Methyl-seq Conversion Module (NEB, E7125)
- UvrD Helicase (NEB, M1202S)
- ATP (100 mM stock)
- MgCl2 (100 mM stock)
- Nuclease-free water

Procedure A: Oxidation/Protection Step

1. On ice, prepare a **45 µL** oxidation reaction mixture:

**Component**	**Volume (µL)**
Purified cfDNA	27
Oxidation Reaction Buffer	5
Oxidation Reaction S	1
Oxidation Reagent	1
Oxidation Enzyme 1	10
Oxidation Enzyme 2	1
**Total**	**45**

2. In a separate 1.5 mL tube on ice, prepare a **1:1000 dilution** of Active Reagent (Fe2+):
    - E.g., combine 1 µL Active Reagent + 999 µL nuclease-free water.

- Mix gently by inversion (do not vortex).

3. Add **5 µL** of freshly diluted Active Reagent to the 45 µL oxidation mixture.
4. Mix thoroughly by pipetting; briefly centrifuge at 500 × *g*.
5. Incubate in a PCR machine at **37°C for 1 hour** (with heated lid at **50°C**).
6. Centrifuge briefly; transfer to ice.
7. Add **1 µL** Stop Reagent.
8. Incubate at **37°C for 30 minutes**.
9. **DNA Purification**:
   - Add **150 µL** AMPure XP beads (3× volume).
   - Follow standard bead purification protocol.
   - Elute in **54 µL** nuclease-free water.
10. Keep at **4°C** or proceed immediately to Procedure B.

Procedure B: Cytosines Deamination Step

1. On ice, prepare a **70 µL** deamination reaction mixture:

Component	Volume (µL)
Purified cfDNA (from Procedure A)	53.55
DEA Reaction Buffer (1×)	7
BSA	0.7
ATP (100 mM)	1.75
MgCl2 (100 mM)	3.5
DEA Enzyme Mix (APOBEC3A)	1
**UvrD Helicase**	**2.5**
**Total**	**70**

Critical Note: UvrD Helicase serves as the denaturation agent in this optimized protocol, replacing the standard kit's separate Denaturation step. This allows simultaneous strand separation and cytosine deamination.

2. Mix thoroughly by pipetting; briefly centrifuge at 500 × *g*.
3. Incubate in a PCR machine at **37°C for 1.5 hours** (with heated lid at **50°C**).
4. Centrifuge briefly.
5. **DNA Purification**:
   - Add **210 µL** AMPure XP beads (3× volume).
   - Follow standard bead purification protocol.
   - Elute in **23.5 µL** nuclease-free water.
6. Keep at **4°C** or proceed immediately to Section 6.

6. PCR Amplification to Generate Complete MASD-seq Library

Objective: Amplify converted cfDNA using unique dual index (UDI) primers to generate the final sequencing-ready library, minimizing index hopping during multiplexing.

Materials:

- Purified DNA (from Section 5, 23.5 µL)
- KAPA HiFi HotStart Uracil+ ReadyMix (Roche, KK2802)
- UDI adapter primers for Illumina
  - Forward: 5'-AATGATACGGCGACCACCGAGATCTACAC[i5]ACACTCTTTCCCTACACGACGCTCTTCCGATCT-3' (SEQ ID NO: 5)
  - Reverse: 5'-CAAGCAGAAGACGGCATACGAGAT[i7]GTGACTGGAGTTCAGACGTGTGCTCTTCCGATC-3' (SEQ ID NO: 6)
  - [i5] and [i7] denote unique 8 bp barcodes; each i5/i7 combination is unique to prevent index hopping
  - Supplied as mixed primer pair at appropriate concentration (typically 10 µM total)

Procedure:

1. On ice, prepare a **50 µL** PCR reaction mixture:

**Component**	**Volume (µL)**
Purified DNA	22.5
UDI adapter primers for Illumina	2.5
KAPA HiFi HotStart Uracil+ ReadyMix	25
**Total**	**50**

2. Mix thoroughly by pipetting; briefly centrifuge at 500 × *g*.
3. Transfer to a PCR machine and run:
   - **98°C for 1 minute** (initial denaturation)
   - **14 cycles** (optimized for 20 ng cfDNA input):
     - 98°C for **20 seconds** (denaturation)
     - 65°C for **30 seconds** (annealing)
     - 72°C for **30 seconds** (extension)
   - **72°C for 1 minute** (final extension)
   - **4°C hold**
4. Centrifuge briefly.
5. **DNA Purification**:
   - Add **60 µL** AMPure XP beads (1.2× volume).
   - Follow standard bead purification protocol.

- Elute in **25 µL** nuclease-free water.

6. Keep at **4°C** until quality control or sequencing.

7. Library Quality Control and Sequencing (MASD-seq)

Procedure:

1. **Quantification**:
   - Measure library concentration using Qubit 4 Fluorometer (dsDNA HS Assay).
2. **Fragment size distribution**:
   - Assess using fragment analyzer.
   - Expected size:~ 300–700 bp.
3. **Sequencing**:
   - Platform: **Illumina NovaSeq X Plus**
   - Mode: **150 bp paired-end reads**
   - Data yield per cfDNA sample: **~100 Gb**

Part II: MspI-RRS Library Construction (Buffy Coat and Tumor Tissue)

Sample Preparation

Materials:

- Genomic DNA from buffy coat (white blood cells) or tumor tissue
- DNeasy Blood & Tissue Kit (QIAGEN, 69506) for DNA extraction
- Qubit 4 Fluorometer

Procedure:

1. Extract genomic DNA from buffy coat or tumor tissue using the DNeasy Blood & Tissue Kit according to the manufacturer's protocol.
2. Quantify DNA using Qubit 4 Fluorometer (dsDNA HS Assay).
3. Store at **−20°C** until MspI-RRS library preparation.

1. 5' End Dephosphorylation (Quick CIP)

Objective: Remove 5' phosphate groups from genomic DNA to prevent non-specific ligation of DNA ends to each other (rather than to adapters).

Materials:

- Genomic DNA (approximate 50 ng input; the reaction volume can accommodate up to ~150 ng)
- Quick CIP (NEB, M0525)
- rCutsmart buffer (10×)
- Nuclease-free water

Procedure:

1. On ice, prepare a **30 µL** reaction mixture:

**Component**	**Volume (µL)**
DNA (~50 ng)	25
rCutsmart buffer (10×)	3
Quick CIP	2
**Total**	**30**

2. Mix thoroughly by pipetting; briefly centrifuge at 500 × *g*.
3. In a PCR machine with heated lid set to **105°C**, incubate at:
   - **37°C for 30 minutes.**
4. Heat inactivate at **80°C for 5 minutes**.
5. Briefly centrifuge; hold at **4°C** or proceed immediately to Section 2.

2. 3' End Blocking with ddGTP (Terminal Transferase)

Objective: Block 3' ends with ddGTP to prevent addition of adenine (A-tailing) to non-target termini and subsequent non-specific ligation.

Materials:

- 5' dephosphorylation reaction product (from Section 1, 25 µL)
- Terminal Transferase (NEB, M0315, ~10 U/µL typical)
- ddGTP (Roche, 3732738001; prepared at **1 mM** final concentration)
- rCutsmart buffer (10×)
- CoCl2 (2.5 mM stock; CoCl2 is a cofactor for Terminal Transferase)
- Nuclease-free water

Procedure:

1. After 5' dephosphorylation, briefly centrifuge.
2. On ice, add the following components to the 30 µL product:

**Component**	**Volume (µL)**
5' dephosphorylation product	30
rCutsmart buffer (10×)	0.5
Terminal Transferase	1
ddGTP (1 mM stock)	0.5
CoCl2 (2.5 mM)	3

3. Mix thoroughly; briefly centrifuge.
4. In a PCR machine with heated lid at **105°C**, incubate at:
    - **37°C for 2 hours** (Terminal Transferase adds ddGTP to 3' ends)
5. Inactivate at **75°C for 20 minutes**.
6. Briefly centrifuge; keep at **4°C** or proceed immediately to Section 3.

3. MspI Digestion

Objective: Digest genomic DNA at CCGG (MspI) recognition sites to enrich for tumor CpG>TpG mutations.

Materials:

- 3' end-blocking reaction product (from Section 2)
- MspI (NEB, R0106M, 100 U/μL)
- rCutsmart buffer (10×)
- Nuclease-free water

Procedure:

1. After 3' end blocking, briefly centrifuge.
2. **DNA Purification** :
    - Add **2× volume** of AMPure XP beads.
    - Follow standard bead purification protocol (mix, 5 min RT, 1–2 min rack, remove supernatant, two 70% ethanol washes, air-dry 5–10 min).
    - Elute in **44 μL** nuclease-free water.
3. **MspI Digestion**: On ice, add to the 44 μL purified DNA:

**Component**	**Volume (μL)**
Purified DNA	44
rCutsmart buffer (10×)	5
MspI (100 U/μL)	1
**Total**	**50**

4. Mix thoroughly; briefly centrifuge.
5. Incubate at **37°C for 15 minutes**.
6. Briefly centrifuge; hold at **4°C** or proceed immediately to Section 4.

4. Dual Size Selection with AMPure XP Beads

Objective: Optimize fragment size distribution by selective precipitation; this step enriches for DNA fragments in the optimal size range for subsequent adapter ligation and sequencing.

Materials:

- MspI digestion product (from Section 3)
- AMPure XP beads

Procedure:

1. Add **0.7× volume** of AMPure XP beads to the MspI digestion product:
    - Mix by pipetting 10 times; incubate at room temperature for **5 minutes**.
    - Place on magnetic rack for **1–2 minutes** until clear.
    - **Collect the supernatant** into a new tube; discard the pellet.
2. To the collected supernatant, add **0.2× volume** of fresh AMPure XP beads:
    - Mix by pipetting; incubate at room temperature for **5 minutes**.
    - Place on magnetic rack for **1–2 minutes** until clear.
    - Remove and discard supernatant.
    - Wash beads with two 200 µL 70% ethanol washes (30 seconds each on rack).
    - Air-dry for **5–10 minutes**.
3. **Elute in 23 µL** nuclease-free water; incubate 2 minutes, transfer eluate.
4. Keep at **4°C** or proceed immediately to Section 5.

5. End Repair and A-overhang Addition (Klenow)

Objective: Repair MspI-generated cohesive ends and add adenine (A) overhangs to 3' ends to facilitate T-A base pairing with adapters.

Materials:

- Purified DNA (from Section 4, 23 µL)
- Klenow Fragment (3'→5' exo−) (NEB, M0212)
- dACGTP mixture: **10 mM dATP, 1 mM dCTP, 1 mM dGTP** (prepare or obtain as ready-made mixture)
- NEBuffer 2 (10×)
- Nuclease-free water

Procedure:

1. On ice, prepare a **30 µL** reaction mixture:

**Component**	**Volume (µL)**
Purified DNA	23
NEBuffer 2 (10×)	3
dACGTP mixture	3
Klenow Fragment (3'→5' exo−)	1
**Total**	**30**

2. Mix thoroughly; briefly centrifuge at 500 × *g*.
3. In a PCR machine with heated lid at **105°C**, incubate at:
    - **30°C for 20 minutes** (initial extension; Klenow fills in overhangs)
    - **37°C for 1 hour** (continued extension to add A-tails)
    - **75°C for 20 minutes** (inactivation of Klenow)
4. Briefly centrifuge; hold at **4°C** or proceed to Section 6.

6. VAHTS Dual UMI Adapter Ligation

Objective: Ligate VAHTS Dual UMI Adapters (containing unique molecular identifiers) to A-tailed DNA fragments to enable molecular tagging and reduce PCR bias.

Materials:

- End-repair/A-tailing reaction product (from Section 5)
- VAHTS Dual UMI UDI Adapters for Illumina (Vazyme, N351)
    - Example adapter sequences:
        - Forward: 5'-ACACTCTTTCCCTACACGACGCTCTTCCGATCTNNNNN(N)-s-T-3'
        - Reverse: 3'-CTGACCTCAAGTCTGCACACGAGAAGGCTAGANNNNN(N)-p-5'
        - Where NNNNN(N) = UMI region; -s- = phosphorothioate modification
- T4 DNA Ligase (NEB, M0202, 2000 U/µL)
- ATP (10 mM stock)
- Nuclease-free water

Procedure:

1. **Add Adapters and Ligase:**

**Component**	**Volume (µL)**
End-repair/A-tailing product	30
ATP (10 mM)	3.5
VAHTS Dual UMI Adapters	0.5
T4 Ligase (2000 U/µL)	1
**Total**	**35**

2. Mix thoroughly; briefly centrifuge.
3. Incubate at **20°C for 15 minutes**.
4. **DNA Purification**:
    - Add **1× volume** of AMPure XP beads.
    - Follow standard bead purification protocol.
    - Elute in **23.5 µL** nuclease-free water.
5. Keep at **4°C** or proceed immediately to Section 7.

7. PCR Amplification to Generate Complete MspI-RRS Library

Objective: Amplify adapter-ligated DNA using UDI primers to generate the final sequencing-ready library.

Materials:

- Purified DNA (from Section 6, 23.5 µL)
- KAPA HiFi HotStart ReadyMix (Roche, KK2602)
- UDI adapter primers for Illumina (same as MASD-seq):
  - Forward: 5'-AATGATACGGCGACCACCGAGATCTACAC[i5]ACACTCTTTCCCTACACGACGCTCTTCCGATCT-3'
  - Reverse: 5'-CAAGCAGAAGACGGCATACGAGAT[i7]GTGACTGGAGTTCAGACGTGTGCTCTTCCGATC-3'

Procedure:

1. On ice, prepare a **50 µL** PCR reaction:

**Component**	**Volume (µL)**
Purified DNA	22.5
UDI adapter primers	2.5
KAPA HiFi HotStart ReadyMix	25
**Total**	**50**

2. Mix thoroughly; briefly centrifuge.
3. Transfer to a PCR machine and run:
   - **98°C for 45 seconds** (initial denaturation)
   - **13 cycles** (optimized for 100 ng genomic DNA input):
     - 98°C for **15 seconds** (denaturation)
     - 60°C for **30 seconds** (annealing)
     - 72°C for **30 seconds** (extension)
   - **72°C for 1 minute** (final extension)
   - **4°C hold**
4. Briefly centrifuge.
5. **DNA Purification**:
   - Add **2× volume** of AMPure XP beads.
   - Follow standard bead purification protocol.
   - Elute in 25 **µL** nuclease-free water.
6. Keep at **4°C** until quality control or sequencing.

8. Library Quality Control and Sequencing (MspI-RRS)

Procedure:

1. **Quantification**:
   - Measure library concentration using Qubit 4 Fluorometer (dsDNA HS Assay).
2. **Fragment size distribution**:
   - Assess using fragment analyzer.
   - Expected size: ~100–400 bp.
3. **Multiplexing**
   - Pool libraries in equimolar amounts for balanced representation.
4. **Sequencing**:
   - Platform: **Illumina NovaSeq X Plus**
   - Mode: **150 bp paired-end reads**
   - Data yield per tissue sample: **~20 Gb**

References for Reagents

**Reagent / Equipment**	**Supplier**	**Catalog #**
DNeasy Blood & Tissue Kit	QIAGEN	69506
VAHTS Free-Circulating DNA Maxi Kit	Vazyme	N903
Quick CIP	NEB	M0525
MspI	NEB	R0106M
CpG Methyltransferase M.SssI	NEB	M0226M
T4 DNA Ligase	NEB	M0202
NotI / NotI-HF	NEB	R3189 / R3189S
Dynabeads MyOne Streptavidin C1	ThermoFisher	65002
NEBNext Enzymatic Methyl-seq Conversion Module	NEB	E7125
UvrD Helicase (20 µg/ml)	NEB	M1202S
Klenow Fragment (3'→5' exo−)	NEB	M0212
Terminal Transferase	NEB	M0315
KAPA HiFi HotStart Uracil+ ReadyMix	Roche	KK2802
KAPA HiFi HotStart ReadyMix	Roche	KK2602

Reagent / Equipment	Supplier	Catalog #
VAHTS Dual UMI Adapters	Vazyme	N351
AMPure XP Beads	Beckman Coulter	A63881
Qubit 4 Fluorometer	ThermoFisher	Q33226
K2-EDTA blood collection tubes	BD	367525
ATP (100 mM)	ThermoFisher	R0441
