# Supplementary material for: Methyltransferase-assisted single duplex sequencing for detecting circulating tumor DNA
Source: EMBO Mol Med. 2026 Aug 19;18(10):3961–78. doi: 10.1038/s44321-026-00509-2 (PMC13658776; doi:10.1038/s44321-026-00509-2)
Supplement: Supplementary file 2 [file 44321_2026_509_MOESM3_ESM.pdf]

EMBO Molecular Medicine

Methyltransferase-Assisted Single Duplex Sequencing for Detecting Circulating Tumor DNA

Zhaoyu Jiang, Yuxuan Wu, Yuqing Guo, Renjie Luo, Wei Wang, Fuchou Tang, Wei Fu, Xin Zhou, and Lu Wen

Corresponding authors: Lu Wen (wenlu@pku.edu.cn) , Xin Zhou (zhouxinasd@sina.cn), Wei Fu (fuwei@bjmu.edu.cn)

Review Timeline:

Submission Date:	22nd Dec 25
Editorial Decision:	9th Feb 26
Revision Received:	2nd Jul 26
Editorial Decision:	20th Jul 26
Revision Received:	27th Jul 26
Accepted:	4th Aug 26

Editor: Jingyi Hou

Transaction Report:

(Note: With the exception of the correction of typographical or spelling errors that could be a source of ambiguity, letters and reports are not edited. Depending on transfer agreements, referee reports obtained elsewhere may or may not be included in this compilation. Referee reports are anonymous unless the Referee chooses to sign their reports.)

9th Feb 2026

Dear Dr. Wen,

Thank you for submitting your work to EMBO Molecular Medicine. First, I would like to apologize for the delay in the review process, which was due to the holiday season. We have now received evaluations from the three reviewers who agreed to assess your manuscript.

As you will see in the reports below, the reviewers recognize the method as novel and potentially interesting; however, they raise concerns regarding validation, benchmarking, overstatements, and insufficient technical details, all of which should be addressed in a revised manuscript.

We would welcome the submission of a revised version in three months for further consideration. As you may already know, our editorial policy allows in principle a single round of major revision, and it is therefore essential to provide responses to the referees' comments that are as complete as possible.

EMBO Molecular Medicine has a "scooping protection" policy, whereby similar findings that are published by others during review or revision are not a criterion for rejection. Should you decide to submit a revised version, I do ask that you get in touch after three months if you have not completed it, to update us on the status.

Please also contact us as soon as possible if similar work is published elsewhere. If other work is published, we may not be able to extend the revision period beyond three months.

Please read below for important editorial formatting and consult our author's guidelines for proper formatting of your revised article for EMBO Molecular Medicine.

I look forward to receiving your revised manuscript.

Yours sincerely,
Jingyi

Jingyi Hou
Senior Editor
EMBO Molecular Medicine

*****
When preparing your revised manuscript, please refer to our guidelines: https://link.springer.com/journal/44321/submission-guidelines#cms-Revised-submissions. We perform an initial quality control of all revised manuscripts before re-review; failure to include requested items will delay the evaluation of your revision.

We require:

1) A .docx formatted version of the manuscript text (including legends for main figures, EV figures and tables). Please make sure that the changes are highlighted to be clearly visible.

2) Individual production quality figure files as .eps, .tif, .jpg (one file per figure). For guidance, download the 'Figure Guide PDF': https://media.springernature.com/original/springer-cms/rest/v1/content/27825798/data/v1.

3) A .docx formatted letter INCLUDING the reviewers' reports and your detailed point-by-point responses to their comments. As part of the EMBO Press transparent editorial process, the point-by-point response is part of the Review Process File (RPF), which will be published alongside your paper.

4) A complete author checklist, which you can download from our author guidelines. Please insert information in the checklist that is also reflected in the manuscript. The completed author checklist will also be part of the RPF.

5) Please note that all corresponding authors are required to supply an ORCID ID for their name upon submission of a revised manuscript.

6) It is mandatory to include a 'Data Availability' section after the Materials and Methods. Before submitting your revision, primary datasets produced in this study need to be deposited in an appropriate public database, and the accession numbers and database listed under 'Data Availability'. Please remember to provide a reviewer password if the datasets are not yet public.

In case you have no data that requires deposition in a public database, please state so in this section. Note that the Data Availability Section is restricted to new primary data that are part of this study.

7) For data quantification: please specify the name of the statistical test used to generate error bars and P values, the number (n) of independent experiments (specify technical or biological replicates) underlying each data point and the test used to calculate p-values in each figure legend. The figure legends should contain a basic description of n, P and the test applied. Graphs must include a description of the bars and the error bars (s.d., s.e.m.).

8) At EMBO Press we ask authors to provide source data for the main manuscript figures. You will receive a separate email with instructions for providing source data with your revised manuscript, including how to upload and organize the files.

9) Our journal encourages inclusion of *data citations in the reference list* to directly cite datasets that were re-used and obtained from public databases. Data citations in the article text are distinct from normal bibliographical citations and should directly link to the database records from which the data can be accessed. In the main text, data citations are formatted as follows: "Data ref: Smith et al, 2001" or "Data ref: NCBI Sequence Read Archive PRJNA342805, 2017". In the Reference list, data citations must be labeled with "[DATASET]". A data reference must provide the database name, accession number/identifiers and a resolvable link to the landing page from which the data can be accessed at the end of the reference.

10) We replaced Supplementary Information with Expanded View (EV) Figures and Tables that are collapsible/expandable online. A maximum of 5 EV Figures can be typeset. EV Figures should be cited as 'Figure EV1, Figure EV2" etc... in the text and their respective legends should be included in the main text after the legends of regular figures.

- For the figures that you do NOT wish to display as Expanded View figures, they should be bundled together with their legends in a single PDF file called *Appendix*, which should start with a short Table of Content. Appendix figures should be referred to in the main text as: "Appendix Figure S1, Appendix Figure S2" etc.

- Additional Tables/Datasets should be labeled and referred to as Table EV1, Dataset EV1, etc. Legends have to be provided in a separate tab in case of .xls files. Alternatively, the legend can be supplied as a separate text file (README) and zipped together with the Table/Dataset file.

11) The paper explained: EMBO Molecular Medicine articles are accompanied by a summary of the articles to emphasize the major findings in the paper and their medical implications for the non-specialist reader. Please provide a draft summary of your article highlighting

- the medical issue you are addressing,

- the results obtained and

- their clinical impact.

This may be edited to ensure that readers understand the significance and context of the research. Please refer to any of our published articles for an example.

12) Author contributions: You will be asked to provide CRediT (Contributor Role Taxonomy) terms in the submission system. These replace a narrative author contribution section in the manuscript.

13) A Conflict of Interest statement should be provided in the main text.

14) Please provide a 'Synopsis' to further enhance discoverability. Synopses are displayed on the journal webpage and are freely accessible to all readers. They include a short stand first (maximum of 300 characters, including space) as well as 2-5 one-sentences bullet points that summarizes the paper. Please write the bullet points to summarize the key NEW findings. They should be designed to be complementary to the abstract - i.e. not repeat the same text. We encourage inclusion of key acronyms and quantitative information (maximum of 30 words / bullet point). Please use the passive voice. Please attach these in a separate file or send them by email, we will incorporate them accordingly.

Please also provide a visual abstract to illustrate your article as a PNG file 550 px wide x 300-600 px high.

15) Please download and fill our Reagents and Tools Table template (.docx), which you can find in our author guidelines:

https://link.springer.com/journal/44320/submission-guidelines#structuredmethods.

EMBO Molecular Medicine has a "scooping protection" policy, whereby similar findings that are published by others during review or revision are not a criterion for rejection. Should you decide to submit a revised version, I do ask that you get in touch after three months if you have not completed it, to update us on the status.

***** Reviewer's comments *****

Referee #1 (Remarks for Author):

This manuscript describes the development and validation of Methyltransferase-Assisted Single Duplex sequencing (MASD-seq) and MspI-Reduced Representation Sequencing (MspI-RRS) assay for high-accuracy ctDNA detection and cost-efficient tumor mutation profiling. By integrating CpG methyltransferase pre-methylation, MASD-seq enable detection of CpG>TpG and transversion mutations in DNA duplexes and employs enzymatic methyl-sequencing to disrupt strand pairing. The Watson and Crick strands are physically linked to enable error correction using double-stranded information within a single read pair, resulting reduced CpG to TpG error rate compared to existing tools. In addition, MspI-RRS enables reduced genome complexity to enrich for CpG sites frequently mutated in tumor samples, which helps in saving sequencing cost. Overall, the conclusions are supported by the data. I'd consider it a good candidate for EMBO molecular medicine. I have several comments for further improvement of the manuscript.

1. EM-seq is known to be of relatively lower conversion rate compared to bisulfite conversion. In addition, it might cause some variation due to different batches of enzyme. Will it be better to present one set of real-world bisulfite sequencing data for MASD-seq to tell the readers the advantage and disadvantage to use EM-seq in the current protocol?
2. I'd suggest combine Fig. S1 to Fig. 1, which can make the method more easily understandable to the readers.
3. "TF" (Tumor Fraction) need to be defined clearly at its first mention in the main text, although it is defined in the figure legend.

Referee #2 (Remarks for Author):

OVERALL:

This manuscript describes a novel method (MASD-seq) for ultra-sensitive detection of circulating tumor DNA (ctDNA) using enzymatic methylation, duplex sequencing (particularly double CpG correction algorithm), and MspI RRS principles. The method is technically innovative, with new designs based on published method frame and is applied to colorectal cancer (CRC) with promising performance. However, there are several conceptual, technical, and presentation issues that must be addressed before the paper is suitable for publication in a high-impact journal such as EMBO Molecular Medicine.

1, MAJOR CONCERNS:

The rationale for the TET2-APOBEC3A method is unclear and requires clarification.

Besides all other technological elements for the MASD-seq, the manuscript describes protecting unmethylated cytosines using (M.sssI followed by) sequential TET2 and APOBEC3A treatment. The fundamental logic and necessity of this specific enzymatic cascade, as opposed to simpler options, are not sufficiently justified. Please address the following specific points to clarify the methodological design:

1) Incomplete TET2 oxidation could compromise protection.
You state that 5-methylcytosine (5mC) is oxidized by TET2 "to 5hmC, 5fC, or 5caC" (lines 120-122). The use of "or" implies this oxidation may not always proceed fully to 5caC. If the reaction performs at an imperfect efficiency or stops at an intermediate state (e.g., 5hmC), the original 5mC site would remain vulnerable to deamination by APOBEC3A, creating false signals. Please clarify the completeness and stoichiometry of the TET2 conversion in your system and explain how you ensure consistent and complete protection.

2)The need for perfect M.SssI methylation is not addressed.
Figure S1 indicates that M.SssI treatment does not achieve 100% methylation efficiency, leaving some Cytosines unprotected. Given that MASD-seq uses bulk DNA with sufficient input, why was the M.SssI reaction not optimized to near-complete efficiency? Perfect methylation by M.SssI would prevent subsequent misclassification of these sites as "damaged," simplifying the assay's interpretation. Please comment on this potential optimization.

3) A direct comparison with bisulfite sequencing is lacking.
If M.SssI successfully methylates all cytosines, then conventional bisulfite sequencing could directly distinguish original mutant/deaminated and process introduced nucleotide (particularly T, U and new unmetylated C) from all original Cs (M.SssI-introduced all original unmethylated Cs to 5mC and protected). Then why not use the simple and golden-standard bisulfite

conversion? A premise seems to be that TET2-APOBEC3A is gentler than bisulfite treatment, preserving DNA integrity. However, current bisulfite protocols can also be gentle, and it consistently achieve very high conversion rates (consistently higher than TET2-APOBEC3A). Furthermore, bisulfite-converted DNA can be PCR-amplified (with U converted to T in PCR) and sequenced it without requiring additional enzymes like UvrD helicase. Please provide a clear rationale for why the added complexity of the TET2-APOBEC3A-UvrD cascade is superior to an optimized bisulfite approach following M.SssI treatment, and provide direct data comparing DNA fragmentation/quality between your method and bisulfite protocol.

4) The enzymatic cascade introduces its own limitations.
Your chosen method introduces multiple extra steps, enzymes, and potential sources of error, e.g., incomplete TET2-APOBEC3A deamination, with overall conversion rate in repeated reports at ~96% (95-97%) vs. bisulfite's consistent ~99.5%. This complexity risks accumulating artificial nucleotide changes and damage. Please discuss the trade-offs of your method's complexity versus its proposed benefits, and quantitatively address the cumulative efficiency and potential error rates of the complete TET2-APOBEC3A conversion step in your system.

MINOR CONCERNS:

1. The authors claim MASD-seq achieves "nearly 100%" duplex recovery efficiency. However it is a MspI-based sequences enrichment method, it can not cover 100% of all DNA sequences of the samples.

2. The claim that "correctable mutation burden" in MspI-digested regions exceeds whole-genome total mutation burden in MSI-H tumors (Fig. 3B) is mathematically true only because they exclude uncorrectable mutations. This is a potentially misleading metric for clinical utility, as it does not reflect the full mutation landscape.

3. MASD-seq can only correct CpG>TpG and transversion errors, but not CpH>TpH or T>C transitions. This means a significant fraction of true somatic mutations (e.g., C>T in non-CpG contexts) will be missed or misclassified as errors. The authors acknowledge this but do not adequately discuss the clinical implications for ctDNA detection, especially in tumors with low CpG>TpG burden.

4. The method's sensitivity depends heavily on CpG>TpG enrichment via MspI digestion. While this works well for MSI-H CRC, its utility in MSS tumors or cancers with low CpG>TpG burden (e.g., lung, breast) is questionable.

5. The report lacks standardized benchmarking for ctDNA detection. Specifically, it presents no experimental comparison with commercially available ctDNA assays or other established research-grade ultrasensitive methods. Furthermore, the in silico mixing validation used is insufficient, as it cannot substitute for real-world limit-of-detection studies performed with spike-in controls.

6. Regarding figure and supplementary material clarity. Some figures (e.g., Fig. S1, S3) are overly complex and not clearly explained in the main text.

7. Regarding methodological details insufficient for replication. The MASD-seq protocol involves multiple enzymatic steps (MspI, M.SssI, TET2, APOBEC3A, UvrD). Critical parameters (reaction efficiencies, enzyme lot variations, optimization steps) are not fully detailed.

8. Regarding statistical and analytical rigor. The use of Pearson correlation for mutation burden comparison (Fig. 3E) may not be appropriate for non-normally distributed count data. No multiple testing correction applied in mutation burden analyses.

9. The abstract claims "highly accurate sequencing method and holds promise for clinical applications", but the clinical data (clinical models) are preliminary.

10. Perform direct comparison with at least one established duplex sequencing method using the same samples. Benchmark against a clinically validated ctDNA assay to establish real-world sensitivity/specificity.

11. Clarify limitations regarding uncorrectable mutations and tumor types where MASD-seq may not be optimal.

12. Provide more detailed protocols in the Methods section to ensure reproducibility.
Tone down overstated claims in the abstract and discussion regarding clinical readiness.

REVIEW CONCLUSIONS:

MASD-seq is a clinical valuable method, with technically innovation, that addresses an important challenge in ctDNA detection. However, the current manuscript overstates its performance and clinical applicability while under-validating key claims. With rigorous benchmarking, larger clinical validation, and clearer acknowledgment of limitations, this work could become a valuable contribution to the field.

RECOMMENDATION:

Major revision required before consideration for publication.

Since the manuscript describes a methodological concept with a preliminary assessment, the authors may provide a comprehensive clarification in their response, but it may not be absolute necessary to accompany with extensive/large new data, to clarify the comments raised, which would alternatively allow for the timely communication of the core concept, while future work on optimization, rigorous benchmarking, and extended validation could be explored in a subsequent, more detailed study.

Referee #3 (Comments on Novelty/Model System for Author):

From a technical standpoint, this strategy is novel and validated using serval cohorts. However, the authors validate their method only on colorectal cancer samples. To assess broader clinical applicability, it is necessary to evaluate the method on samples from other cancer types and to determine whether it introduces bias in other mutational signatures, given that different types of substitutions are corrected differently by their method. More technical details are required to evaluate the validity of the method.

Referee #3 (Remarks for Author):

The authors describe MASD-seq, a sequencing strategy integrating duplex consensus correction and CpG-targeted reduced representation sequencing for sensitive somatic mutation detection. The study addresses an important methodological challenge and is supported by validation in cell lines, clinical colorectal cancer samples, and large external datasets.

1. Evidence supporting the biological enrichment of CpG>TpG mutations is primarily presented in the supplementary material (e.g. Supplementary Figure S6). Given the centrality of this point to the manuscript's main claims, clearer integration of these results into the main text or figures would strengthen the narrative and improve accessibility for readers.
2. The manuscript supports the cost-efficiency of MASD-seq and MspI-RRS through sequencing depth usage, duplication rates, and data volume comparisons relative to WGS. However, these points are largely presented late in the Discussion and are not clearly summarized in the Results. Explicitly presenting sequencing depth or data volume comparisons in the main results (e.g. via a summary table or schematic) would strengthen and clarify this claim.
3. The benefit of duplex consensus correction is convincingly demonstrated in cell line models; however, its contribution to improved accuracy in primary tumor tissue or cfDNA samples is less explicitly shown. Clearer contextualization of this limitation, or additional clarification of how duplex correction impacts clinical samples, would improve interpretation.
4. The selected cell lines (GM12878 and HEK293T) are appropriate for technical validation of error correction but have limited relevance to tumour biology. Explicitly distinguishing technical validation from biological interpretation would improve clarity."
5. To assess broader clinical applicability, it is necessary to evaluate the method on samples from other cancer types and to determine whether it introduces bias in other mutational signatures, given that different types of substitutions are corrected differently by the method.
6. There is no explanation about how the error rate is calculated, despite it being a fundamental concept underlying the proposed methodology. In the absence of this explanation, it is not possible to evaluate the validity of the method.

RESPONSE TO REVIEWER COMMENTS

Summary of Revisions:

We appreciate the valuable feedback from all three reviewers on our study. In light of your suggestions and comments, we have thoroughly revised and improved the manuscript in the following key aspects.

1. **We performed a head-to-head comparison of EM-seq and bisulfite (BS) sequencing for MASD-seq library construction**. We subjected 60 ng of GM12878 genomic DNA (gDNA) to MASD-seq library construction using either EM-seq or BS. The results showed that BS caused greater DNA degradation and a 6-fold higher duplication rate, although the two methods exhibited comparable accuracy for detecting CpG>TpG and transversion mutations, as well as comparable methylation rates and CH conversion rates.
2. **We conducted a spike-in experiment benchmarking against an established duplex sequencing assay**. To evaluate the performance of MASD-seq, we conducted spike-in experiments using tumor gDNA from Patient 1, diluted into 60 ng of normal white blood cell (WBC) gDNA at mutant allele fractions of 0.025%, 0.0125%, 0.0063%, and 0%. As a comparator, we employed a commercial hybrid-capture-based targeted sequencing assay capable of both single-strand consensus (SSC) and double-strand consensus (DSC) analysis. At the 0.0063% level, SSC failed to achieve clear detection due to excessive background noise, whereas both MASD-seq and DSC exhibited substantially lower backgrounds and successfully detected the spiked-in mutations. Notably, at matched sequencing depth, MASD-seq recovered approximately tenfold more true molecules than DSC. This translated into markedly higher signal-to-noise ratios for MASD-seq than for both SSC and DSC, demonstrating its superior performance for ultra-low-frequency mutation detection.
3. **We provided more comprehensive explanations, data interpretation, and technical details of MASD-seq and MspI-RRS**. We described how MASD-seq accounts for incomplete TET2 oxidation, specifically through a double-CpG correction algorithm that improves conversion accuracy. We clarified that, given the incomplete TET2 oxidation during EM-seq processing, a net methylation level of approximately 99% indicates that the effective M.SssI methylation rate is already near-complete. We also noted that the CH conversion rate is not a critical parameter for evaluating MASD-seq performance. Specifically, Figures S1 and S3 have been improved and moved to the main figures, with clarified explanations in the revised manuscript. For MspI-RRS, we provided sequencing-depth information to support its cost-efficiency. Importantly, a detailed protocol for both MASD-seq and MspI-RRS has been included in the Supplementary Materials.
4. **We assessed the broader clinical applicability of MASD-seq and MspI-RRS**. We performed mutation burden analysis on the IGCG database and the results showed that the mutation burden enrichment in the MspI-region varies across human tumor types, with that CRC being among the cancer types with the highest enrichment. Other cancer types, including pancreatic adenocarcinoma and prostate adenocarcinoma also exhibited relatively high enrichment ratios.

We also applied MspI-RRS to gastric cancer (GC) and hepatocellular carcinoma (HCC). These results show that GC is more amenable to MspI-RRS mutation enrichment than HCC.

5. **Other improvements**:

- We provided clearer explanations of the definition of tumor fraction (TF) and the calculation of TF and error rates.
- We addressed the limitations associated with uncorrectable mutations and tempered the language concerning clinical readiness.

We hope that the extensive revisions and additional data we have provided meet the standards for publication.

Response to Referee #1

Referee #1 (Remarks for Author):

This manuscript describes the development and validation of Methyltransferase-Assisted Single Duplex sequencing (MASD-seq) and MspI-Reduced Representation Sequencing (MspI-RRS) assay for high-accuracy ctDNA detection and cost-efficient tumor mutation profiling. By integrating CpG methyltransferase pre-methylation, MASD-seq enable detection of CpG>TpG and transversion mutations in DNA duplexes and employs enzymatic methyl-sequencing to disrupt strand pairing. The Watson and Crick strands are physically linked to enable error correction using double-stranded information within a single read pair, resulting reduced CpG to TpG error rate compared to existing tools. In addition, MspI-RRS enables reduced genome complexity to enrich for CpG sites frequently mutated in tumor samples, which helps in saving sequencing cost. Overall, the conclusions are supported by the data. I'd consider it a good candidate for EMBO molecular medicine. I have several comments for further improvement of the manuscript.

Response: We sincerely thank the reviewer for the positive and constructive comments.

R1C1. EM-seq is known to be of relatively lower conversion rate compared to bisulfite conversion. In addition, it might cause some variation due to different batches of enzyme. Will it be better to present one set of real-world bisulfite sequencing data for MASD-seq to tell the readers the advantage and disadvantage to use EM-seq in the current protocol?

Response: We thank the reviewer for this helpful suggestion. Following your suggestion, we performed a head-to-head comparison of EM-seq and bisulfite(BS) sequencing for MASD-seq library construction using GM12878 gDNA (Fig. R1).

The fragment analysis showed that PCR products of BS-based MASD-seq exhibited a shift toward shorter fragments compared with the EM-seq library, indicating DNA degradation (Fig. R1A). With approximately 20 Gb of raw sequencing data per library from 60 ng of starting gDNA, BS exhibited an approximately 6-fold higher PCR duplication rate than EM-seq (average of 10.3 reads per unique molecule identifier (UMI) for BS versus 1.7 for EM-seq; Fig. R1B). EM-seq also gave a higher resolve rate than BS (96.5% for EM-seq vs. 93.5% for BS), indicating its higher data quality (Fig. R1C).

Both methods yielded high methylation levels, with EM-seq showing higher values than BS (98.9% for EM-seq vs. 95.6% for BS). The Watson-only unmethylation rate was 1.89% for BS vs. 0.29% for EM-seq, and the Crick-only unmethylation rate was 1.89% for BS vs. 0.26% for EM-seq; the double-strand unmethylation rate was 0.56% for EM-seq and 0.65% for BS (Fig. R1D). Although EM-seq has been reported to have relatively lower conversion efficiency than BS, our results showed that CH conversion rates were similarly high for both methods (average 99.5% for EM-seq and 99.4% for BS; Fig. R1E).

The two methods showed comparable error rates for correctable error types, including A>C/T>G (6.8×10^{-8} for BS vs 6.2×10^{-8} for EM-seq), A>T/T>A (7.3×10^{-8} for BS vs 7.7×10^{-8} for EM-seq), C>A/G>T (2.6×10^{-7} for BS vs 3.3×10^{-7} for EM-seq), C>G/G>C (1.1×10^{-7} for BS vs 1.1×10^{-7} for EM-seq), as well as CpG>TpG (1.3×10^{-6} for BS vs 9.4×10^{-7} for EM-seq; Fig. R1F). Interestingly, BS treatment exhibited markedly higher A>G error rate comparing to EM-seq (1.7×10^{-3} for BS vs 3.5×10^{-5} for EM-seq; Fig. R1F).

Together, these results show that EM-seq and BS exhibited comparable accuracy for detecting CpG>TpG and transversion mutations, as well as comparable methylation rates and CH conversion rates. However, BS results in higher DNA degradation and a higher duplication rate. We have added these results in Fig. S4 and Table EV1 in the revised manuscript.

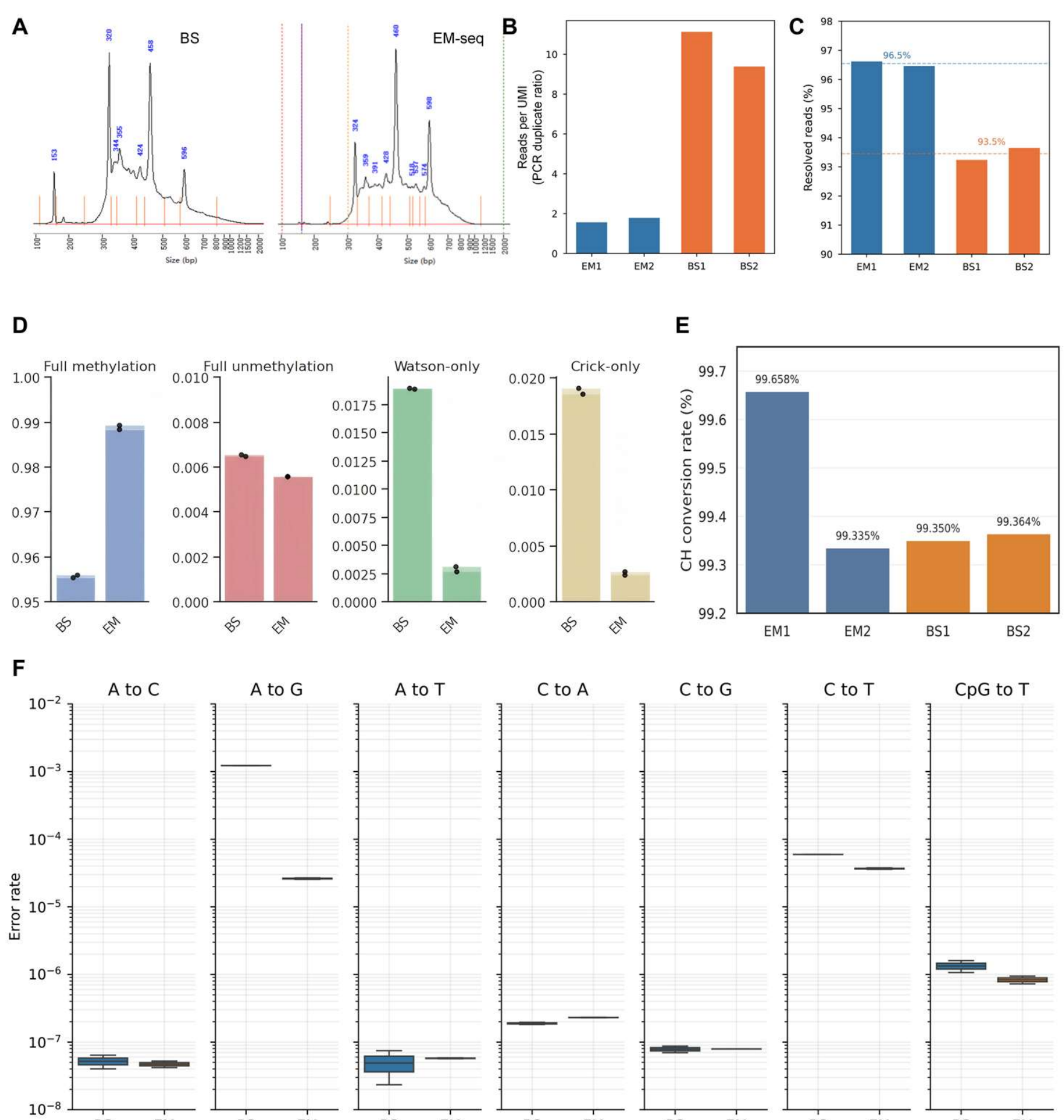

Figure R1. Comparison of EM-seq and BS-seq in MASD-seq library construction. MASD-seq libraries were prepared using either EM-seq or bisulfite (BS) conversion-based sequencing. **A.** Capillary electrophoresis fragment profiles. **B.** PCR duplication rate, shown as reads per unique molecule family. **C.** Hairpin-resolved molecule rate. **D.** Methylation rates across different strand contexts. **E.** CH conversion rate, used to assess conversion efficiency because CpG sites were artificially methylated. **F.** Merged error rates across mutation types and trinucleotide contexts. Bars indicate replicate measurements where applicable.

R1C2. I'd suggest combine Fig. S1 to Fig. 1, which can make the method more easily understandable to the readers.

Response: We thank the reviewer for this suggestion. We have incorporated the original Fig. S1 into Fig. 1 to make the MASD-seq workflow easier to understand (see Fig. R2 and the revised Fig. 1).

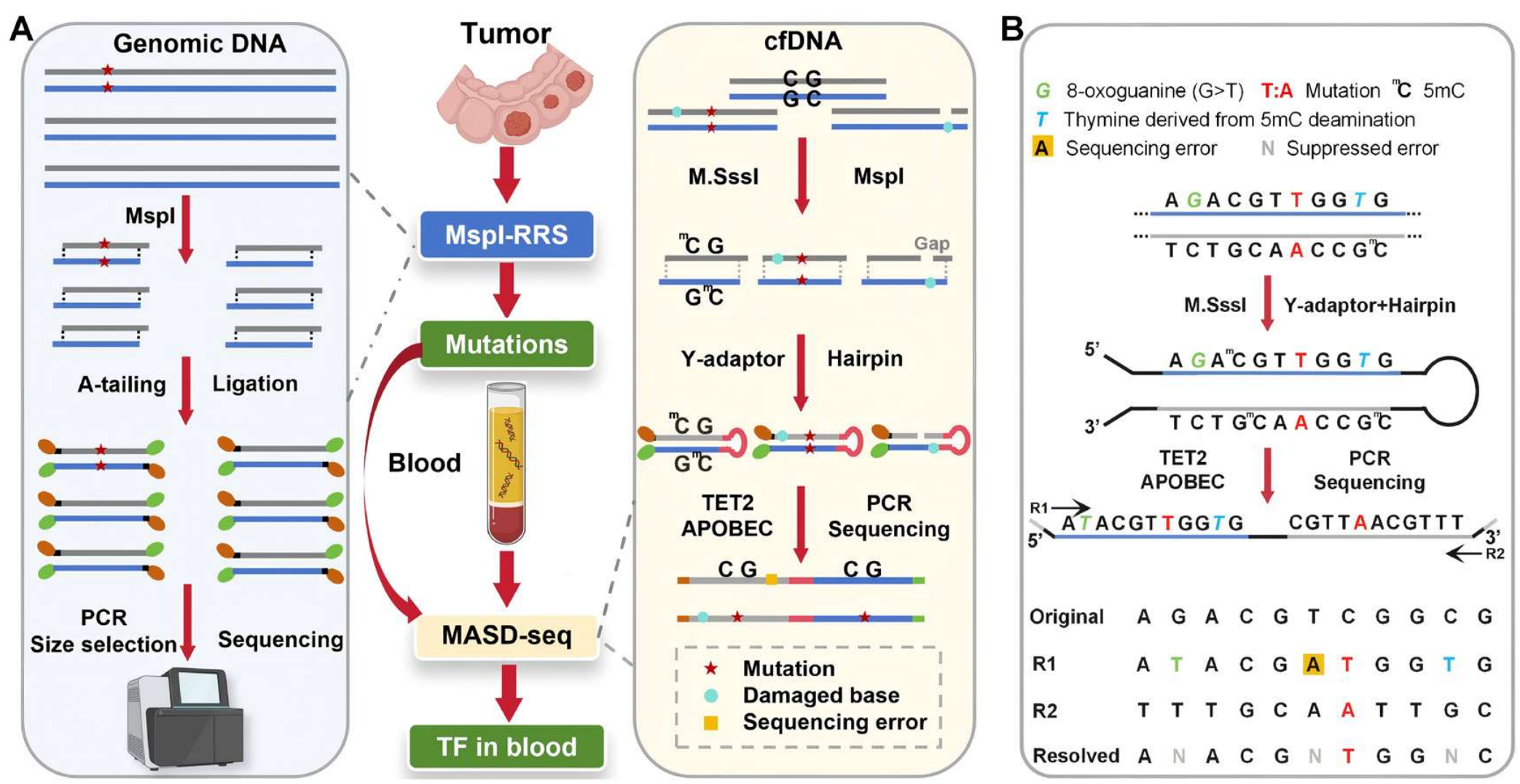

Figure R2. Workflow schematic for MASD-seq and MspI-RRS. A. For tissue samples, a RRS assay using the restriction enzyme MspI (recognizing C|CGG, MspI-RRS) is performed to identify tumor-specific mutations (left panel). The MspI digestion enriches for both the CpG>TpG mutation burden and the total mutation burden while reducing genome complexity. For plasma cfDNAs, MASD-seq that enables efficient single-read error correction through physically linking the original DNA double strands, is employed to quantify the tumor fraction (TF, which is the ratio of mutant alleles to the total number of alleles) in blood (right panel). **B.** The MASD-seq involves three main steps: (1) following MspI digestion, hairpin adapters are ligated to double-stranded DNA; (2) CpG methyltransferase (M.SssI) methylates all CpG sites; and (3) TET2 and APOBEC/UvrD helicase converts unmethylated cytosines to uracil to enable PCR amplification and sequencing. Physical linkage of Watson-Crick strands via hairpin adapters enables error correction in a single-duplex read. Since all cytosines in CpG dinucleotides remain methylated (and thus protected), the method enables correction of the CpG>TpG error, as well as all transversion errors (C>A, C>G, T>G, and T>A). The resolve rules dictated that non-permissible pairs, such as TpG-CpG, T-T and A-A, were masked (marked as N) at the affected position.

R1C3. "TF" (Tumor Fraction) need to be defined clearly at its first mention in the main text, although it is defined in the figure legend.

Response: We thank the reviewer for this comment. TF (Tumor Fraction) is defined as the proportion of mutant alleles to the total number of alleles. We have defined it at its first mention in the maintext in Line 141 and Line 350 in the revised manuscript. Also, we have made a clearer description on how it is calculated in the Method (Line 759) in the revised manuscript.

Response to Referee #2

Referee #2 (Remarks for Author):

This manuscript describes a novel method (MASD-seq) for ultra-sensitive detection of circulating tumor DNA (ctDNA) using enzymatic methylation, duplex sequencing (particularly double CpG correction algorithm), and MspI RRS principles. The method is technically innovative, with new designs based on published method frame and is applied to colorectal cancer (CRC) with promising performance. However, there are several conceptual, technical, and presentation issues that must be addressed before the paper is suitable for publication in a high-impact journal such as EMBO Molecular Medicine.

Response: We sincerely thank the reviewer for the positive and constructive comments.

MAJOR CONCERNS: The rationale for the TET2-APOBEC3A method is unclear and requires clarification. Besides all other technological elements for the MASD-seq, the manuscript describes protecting unmethylated cytosines using (M.sssI followed by) sequential TET2 and APOBEC3A treatment. The fundamental logic and necessity of this specific enzymatic cascade, as opposed to simpler options, are not sufficiently justified. Please address the following specific points to clarify the methodological design:

R2C1. Incomplete TET2 oxidation could compromise protection. You state that 5-methylcytosine (5mC) is oxidized by TET2 "to 5hmC, 5fC, or 5caC" (lines 120-122). The use of "or" implies this oxidation may not always proceed fully to 5caC. If the reaction performs at an imperfect efficiency or stops at an intermediate state (e.g., 5hmC), the original 5mC site would remain vulnerable to deamination by APOBEC3A, creating false signals. Please clarify the completeness and stoichiometry of the TET2 conversion in your system and explain how you ensure consistent and complete protection.

Response:

We thank the reviewer for raising this important point. We completely agree that incomplete TET2 oxidation will lead to false signals. TET2 has been reported to convert more than 99% of 5mC to its oxidized forms (5hmC, 5fC, or 5caC), and the combined enzymatic activity of TET2 and T4-BGT on NA12878 DNA showed that only 0.9% of residual 5mC remains available for deamination by APOBEC3A (Vaisvila et al., 2017, *Genome Research)*. Consistent with this study, our MASD-seq data from GM12878 and HEK293 showed an average CpG methylation protection level of 98.5%. Therefore, using the complementary G on the opposite strand for single-strand correction, and given that the G>A error rate is approximately 10^{-4}, the 1.5% residual false signal translates to a double-strand error rate of 1.5×10^{-6}. It was noteworthy that we have implemented a double-CpG correction algorithm to further improve correction accuracy. This algorithm leverages information from two complementary methylated CpG sites. A genuine CpG>TpG mutation will result in loss of the CpG dinucleotide on both strands, whereas hemi-methylation or DNA damage affects only one CpG site. Our results showed that approximately 0.2% of CpG sites were unmethylated on both strands in the GM12878 and HEK293 MASD-seq data, therefore reducing the error rate to 2×10^{-7}. This value is

consistent with the observed overall CpG>TpG error rate of 5.4×10^{-7} in GM12878 and HEK293 cell lines.

To present these findings more clearly, we have incorporated Fig. S3 into revised Fig. 2 (also see Fig. R3) and added further details in the main text (Line 176).

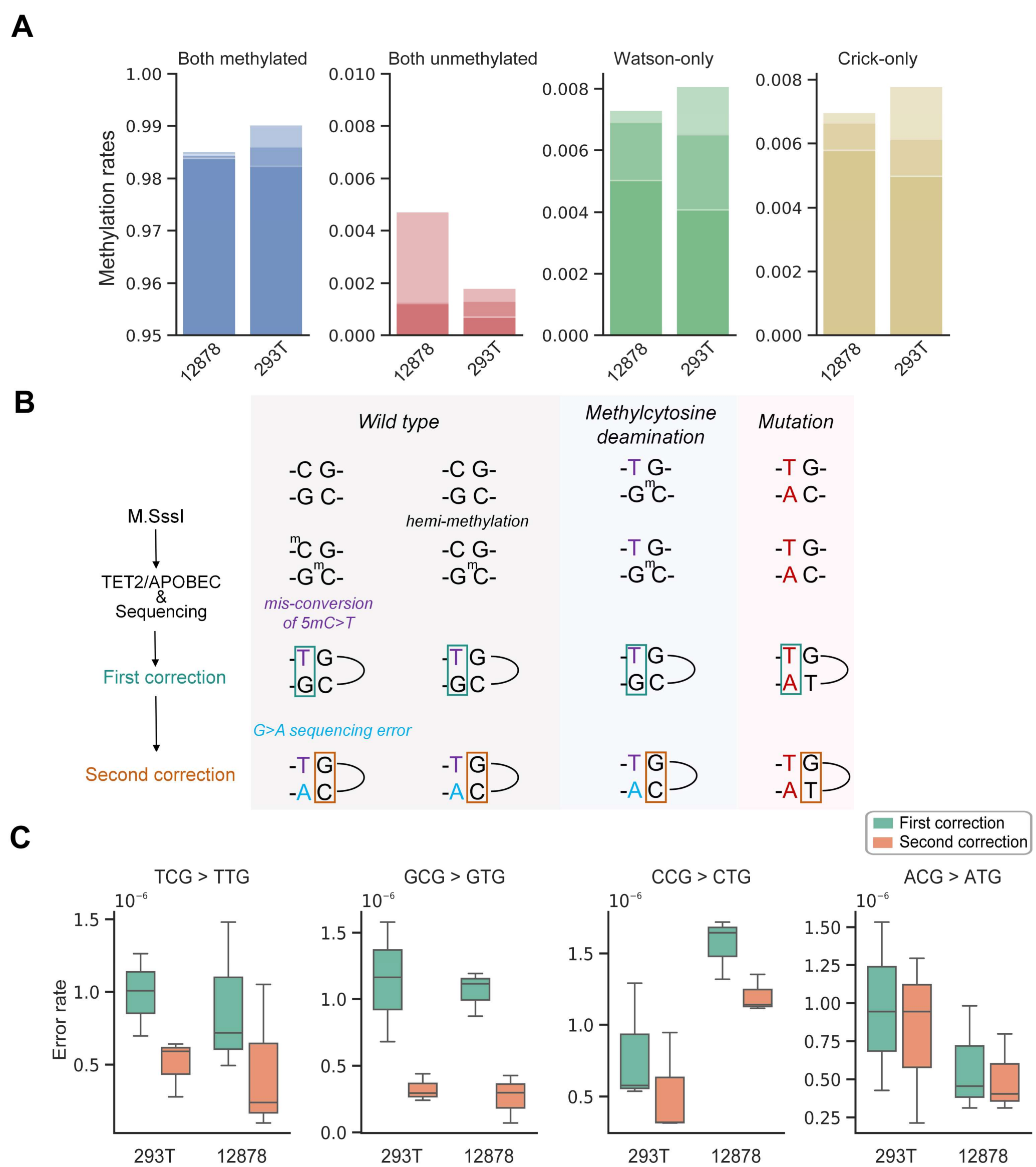

Figure R3. MASD-seq accuracy. A. DNA methylation status in GM12878 and HEK293T cell lines following methyltransferase treatment. Four patterns are shown: methylated on both strands, unmethylated on both strands, and methylated on single-strand. Different shades represent three independent replicates. **B.** Schematic of the double-CpG correction algorithm. Three scenarios are illustrated: wild-type CpG sites, methylcytosine deamination, and genuine mutation. In wild-type molecules, false-positive CpG>TpG signals may arise from incomplete protection or mis-conversion of 5mC to T, as well as from hemi-methylated CpG sites. After M.SssI treatment, the complementary cytosine at a hemi-methylated CpG can be methylated and protected from

TET2/APOBEC conversion. In the First correction step, these false-positive events are identified by the presence of a retained CpG dinucleotide on the complementary strand and are distinguished from true CpG>TpG mutations. In contrast, a genuine mutation permanently disrupts the CpG dinucleotide, preventing M.SssI-mediated methylation and resulting in loss of the CpG signal on both strands. The Second correction step further removes sequencing errors, such as G>A artifacts, by checking whether the complementary strand still contains a protected cytosine. If a retained C is detected on the complementary strand, the apparent mutation is classified as an error and filtered out. **C.** Detected CpG>TpG mutation frequencies in different contexts for both cell lines before (green) and after (orange) using the double correction algorithm.

R2C2. The need for perfect M.SssI methylation is not addressed. Figure S1 indicates that M.SssI treatment does not achieve 100% methylation efficiency, leaving some Cytosines unprotected. Given that MASD-seq uses bulk DNA with sufficient input, why was the M.SssI reaction not optimized to near-complete efficiency? Perfect methylation by M.SssI would prevent subsequent misclassification of these sites as "damaged," simplifying the assay's interpretation. Please comment on this potential optimization.

Response: We thank the reviewer for this constructive comment. We would like to make a clarification to clear up a small confusion from our previous description. First, the methylation rate achieved in 1 h/8 U condition was as high as 99.7%. Second, the unmethylation read should not be attributed to the M.SssI inefficiency alone, but should rather constitute the overall residual signal from the full MASD-seq workflow, particularly including the false 5mC>T signal generated by incomplete TET2 oxidation during EM-seq processing. As discussed in the previous comments, the incomplete TET2 oxidation rate is approximately 1%, indicating that the effective M.SssI conversion rate is already near-complete.

Consistent with this interpretation, we did not observe a trend of increasing methylation rates with longer reaction times or higher enzyme concentrations beyond the 1 h/8 U condition. Considering the benefit of shorter incubation times for workflow efficiency, we selected the 1 h/8 U condition for subsequent experiments.

Of note, under the current condition, the lack of 100% methylation does not compromise the validity of the method regarding somatic mutation detection. In response to the previous comment, we have explained that applying a double correction algorithm can significantly reduce the error rate. In cell line experiments, the expected error rate is 2×10^{-7} and the actual detected error rate is 5.4×10^{-7}, suggesting that CpG>TpG transitions already exist in cell lines. Further, we showed that the resulting accuracy is sufficient to detect age-associated increases in somatic mutation signatures in normal human peripheral blood cfDNA. This indicates that, in clinical blood samples, the background will be primarily derived from age-accumulated somatic mutations, rather than the intrinsic residual artifact of the MASD-seq workflow. We have incorporated the relevant result description (Line 164) and discussion (Line 502) into the manuscript.

R2C3. A direct comparison with bisulfite sequencing is lacking. If M.SssI successfully methylates all cytosines, then conventional bisulfite sequencing could directly distinguish original mutant/deaminated and process introduced nucleotide (particularly T, U and new unmetylated C) from all original Cs (M.SssI-introduced all original unmethylated Cs to 5mC and protected). Then why not use the simple and golden-standard bisulfite conversion? A premise seems to be that TET2-APOBEC3A is gentler than bisulfite treatment, preserving DNA integrity. However, current bisulfite protocols can also be gentle, and it consistently achieve very high conversion rates (consistently higher than TET2-APOBEC3A). Furthermore, bisulfite-converted DNA can be PCR-amplified (with U converted to T in PCR) and sequenced it without requiring additional enzymes like UvrD helicase. Please provide a clear rationale for why the added complexity of the TET2-APOBEC3A-UvrD cascade is superior to an optimized bisulfite approach following M.SssI treatment, and provide direct data comparing DNA fragmentation/quality between your method and bisulfite protocol.

Response: We thank the reviewer for this helpful suggestion. Following your suggestion, we performed a head-to-head comparison of EM-seq and bisulfite (BS) sequencing for MASD-seq library construction using GM12878 gDNA (Fig. R4).

The fragment analysis showed that PCR products of BS-based MASD-seq exhibited a shift toward shorter fragments compared with the EM-seq library, indicating DNA degradation (Fig. R4A). With approximately 20 Gb of raw sequencing data per library from 60 ng of starting gDNA, BS exhibited an approximately 6-fold higher PCR duplication rate than EM-seq (average of 10.3 reads per unique molecule identifier (UMI) for BS versus 1.7 for EM-seq; Fig. R4B). EM-seq also gave a higher resolve rate than BS (96.5% for EM-seq vs. 93.5% for BS), indicating its higher data quality (Fig. R4C).

Both methods yielded high methylation levels, with EM-seq showing higher values than BS (95.6% for BS vs. 98.9% for EM-seq). The Watson-only unmethylation rate was 1.89% for BS vs. 0.29% for EM-seq, and the Crick-only unmethylation rate was 1.89% for BS vs. 0.26% for EM-seq; the double-strand unmethylation rate was 0.56% for EM-seq and 0.65% for BS (Fig. R4D). The CH conversion rates were nearly identical for two methods (99.4% for BS vs 99.5% for EM-seq; Fig. R4E).

The two methods showed comparable error rates for correctable error types, including A>C/T>G (6.8×10^{-8} for BS vs 6.2×10^{-8} for EM-seq), A>T/T>A (7.3×10^{-8} for BS vs 7.7×10^{-8} for EM-seq), C>A/G>T (2.6×10^{-7} for BS vs 3.3×10^{-7} for EM-seq), C>G/G>C (1.1×10^{-7} for BS vs 1.1×10^{-7} for EM-seq), as well as CpG>TpG (1.3×10^{-6} for BS vs 9.4×10^{-7} for EM-seq; Fig. R4F). Interestingly, BS treatment exhibited markedly higher A>G error rate comparing to EM-seq (1.7×10^{-3} for BS vs 3.5×10^{-5} for EM-seq; Fig. R4F).

These results show that EM-seq and BS exhibited comparable accuracy for detecting CpG>TpG and transversion mutations, as well as comparable methylation rates and CH conversion rates. However, BS resulted in higher DNA degradation and a higher duplication rate. Although the CH conversion rates were nearly identical for two methods in our experiments, other studies have indeed reported lower conversion rates for EM-seq compared to BS. Nevertheless, as will be discussed in

response to the following comment, the CH conversion rate is not a critical parameter for MASD-seq performance. We have added these results in Fig. S4 and Table EV1 in the revised manuscript (Line 236).

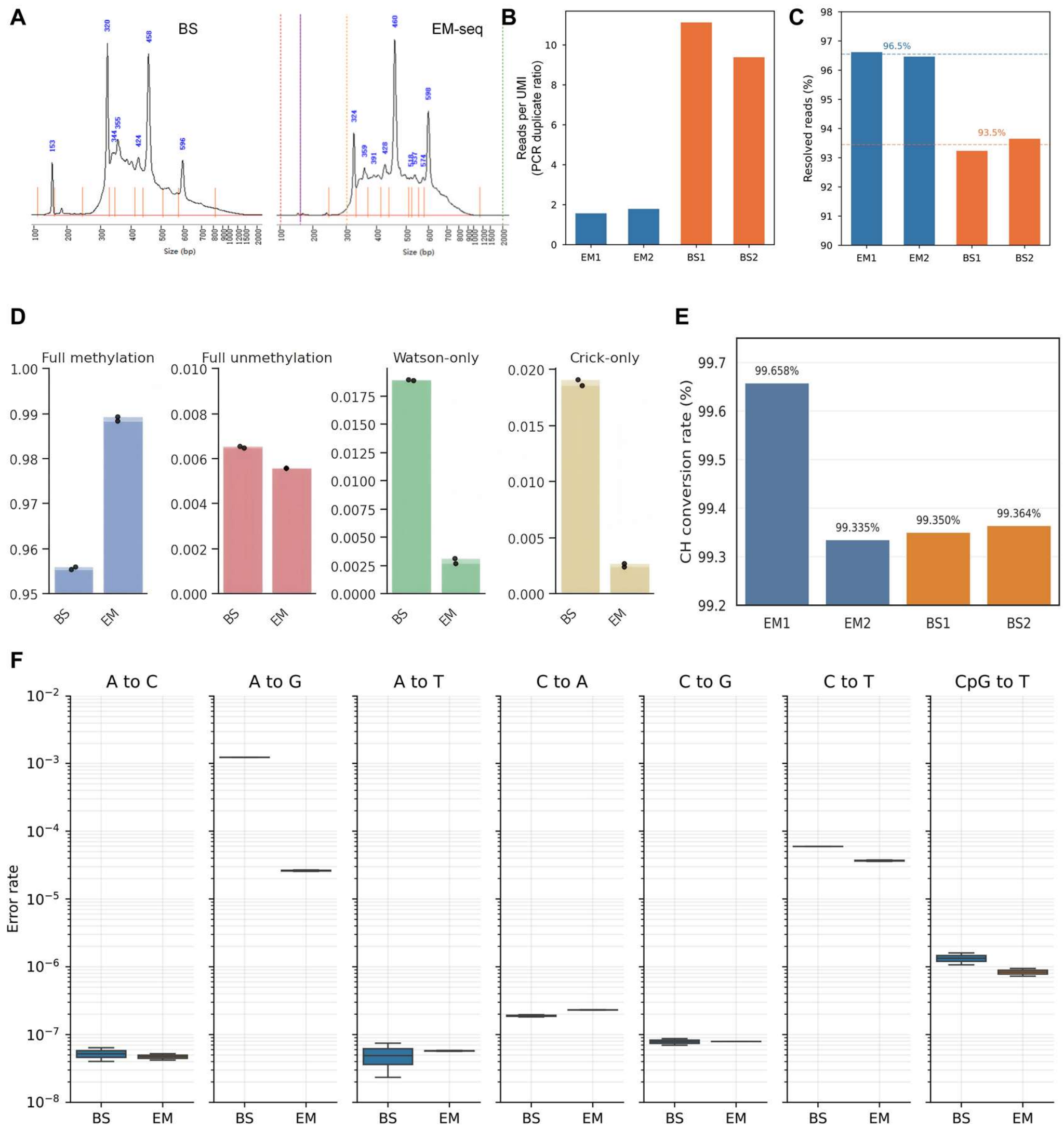

Figure R4. Comparison of EM-seq and BS-seq in MASD-seq library construction. MASD-seq libraries were prepared using either EM-seq or bisulfite conversion-based sequencing (BS-seq). **A.** Capillary electrophoresis fragment profiles. **B.** PCR duplication rate, shown as reads per unique molecule family. **C.** Hairpin-resolved molecule rate. **D.** Methylation rates across different strand contexts. **E.** CH conversion rate, used to assess conversion efficiency because CpG sites were artificially methylated. **F.** Merged error rates across mutation types and trinucleotide contexts. Bars indicate replicate measurements where applicable.

R2C4. The enzymatic cascade introduces its own limitations. Your chosen method introduces multiple extra steps, enzymes, and potential sources of error, e.g., incomplete TET2-APOBEC3A deamination, with overall conversion rate in repeated reports at ~96% (95-97%) vs. bisulfite's consistent ~99.5%. This complexity risks accumulating artificial nucleotide changes and damage. Please discuss the trade-offs of your method's complexity versus its proposed benefits, and quantitatively address the cumulative efficiency and potential error rates of the complete TET2-APOBEC3A conversion step in your system.

Response: We thank the reviewer for this feedback. Despite that the CH conversion rates were nearly identical for two methods in our experiments, other studies have indeed reported lower conversion rates for EM-seq compared to BS. We did not previously make it clear that this conversion rate is not a critical parameter for MASD-seq performance. In conventional DNA methylation analysis, lower CH conversion directly increases false positives because non-methylated CpGs or CpHs will be mis-called as methylated. However, MASD-seq is not designed to measure absolute methylation levels; instead, to achieve its goal of duplex error-correction, it converts non-methylated CpGs into methylated ones. For CpH sites, the conversion of non-methylated cytosines serves to open the double strand for sequencing; thus, the small residual non-conversion is not expected to affect double strand opening efficiency. We have added this clarification to the Result (Line 236), Fig.S4, and Table EV1 in the revised manuscript.

MINOR CONCERNS:

R2c1. The authors claim MASD-seq achieves "nearly 100%" duplex recovery efficiency. However it is a MspI-based sequences enrichment method, it can not cover 100% of all DNA sequences of the samples.

Response: We thank the reviewer for this feedback. We apologize for the misleading expression and have removed the phrase "nearly 100%". The sentence has been revised as follows: “By physically linking the original Watson and Crick strands, MASD-seq enables the identification of DCS in a single-read pair, which allows DCS recovery from as few as one read and improves the efficiency by more than one order of magnitude (Line 482).”

R2c2. The claim that "correctable mutation burden" in Mspl-digested regions exceeds whole-genome total mutation burden in MSI-H tumors (Fig. 3B) is mathematically true only because they exclude uncorrectable mutations. This is a potentially misleading metric for clinical utility, as it does not reflect the full mutation landscape.

Response: We thank the reviewer for this feedback. We have incorporated this point into Paragraph 5 of the Discussion in the revised manuscript (Line 522) as follows: “Consequently, the correctable mutation burden (CpG>TpG and transversions) in the MspI-digested region were comparable to the total mutation burden (all transitions and transversions) across the whole genome in MSI-H and MSS CRC tumors, although it does not reflect the full mutation landscape” .

Furthermore, we acknowledge the inherent limitation that MspI digestion cannot capture the complete mutational landscape. In clinical scenarios where comprehensive mutation profiling—

including the identification of driver gene mutations—is required, we propose that whole-genome sequencing (WGS) be used for tissue mutation analysis, followed by MASD-seq using MspI digestion for cfDNA analysis. This has been discussed in Paragraph 6 of the Discussion (Line 535).

R2c3&c4. MASD-seq can only correct CpG>TpG and transversion errors, but not CpH>TpH or T>C transitions. This means a significant fraction of true somatic mutations (e.g., C>T in non-CpG contexts) will be missed or misclassified as errors. The authors acknowledge this but do not adequately discuss the clinical implications for ctDNA detection, especially in tumors with low CpG>TpG burden.

The method's sensitivity depends heavily on CpG>TpG enrichment via Mspl digestion. While this works well for MSI-H CRC, its utility in MSS tumors or cancers with low CpG>TpG burden (e.g., lung, breast) is questionable.

Response: We thank the reviewer for this feedback. In our previous manuscript, we mainly focused on colorectal cancer (CRC), as it has been reported to be highly enriched for CpG>TpG mutations, and our primary research area is gastrointestinal cancers. In the revised manuscript, we performed more comprehensive analyses on cancer types in the IGCG database to examine their CpG>TpG enrichment. The results showed that the MspI region enriches CpG>TpG mutations by 1.9- to 4.76-fold across different cancer types (Fig. R5A). This region enriches the total mutation burden by 0.66- to 2.05-fold, with 16 out of 25 cancer types exhibiting a fold enrichment greater than 1 (Fig. R5B). Furthermore, when considering the correctable mutation burden, myelodysplastic syndromes (MDS), myeloproliferative neoplasms (MPN), pancreatic adenocarcinoma, and medulloblastoma exhibited an enrichment ratio higher than 0.9, with values of 1.32, 0.92, 0.97, and 0.92, respectively. Five cancer types including prostate adenocarcinoma, acute myeloid leukemia (AML), and breast invasive lobular carcinoma exhibited an enrichment ratio between 0.8 and 0.9 (Fig. R5C). In comparison, the CpG>TpG enrichment ratio, total burden enrichment, and correctable mutation burden ratio were 4, 1.6, and 1.14, respectively, for MSH-I CRC, and 3, 1.2, and 0.95 for MSS CRC. These findings confirm that the CpG>TpG mutation burden varies across human tumor types, with CRC being among the cancer types with the highest burden.

Based on these observations, we propose that MASD-seq is expected to achieve relatively high efficiency in cancer types with a correctable mutation ratio higher than 0.9, particularly pancreatic adenocarcinoma, which exhibits values of 3.3, 1.28, and 0.97 for the CpG>TpG enrichment, the total mutation burden enrichment, and the correctable mutation ratio, respectively. For cancer types with slightly lower enrichment metrics, such as prostate adenocarcinoma (CpG>TpG enrichment: 3.0; total mutation burden enrichment: 1.11; correctable mutation ratio: 0.83), MASD-seq would still yield acceptable efficiency. For cancers with a low CpG>TpG burden, such as lung cancer, CpG>TpG enrichment is not necessary. However, alternative EM-seq-based hairpin strategies may be suitable, as the mutational spectrum of lung cancer is dominated by C>A transversions, which is correctable by the approach.

We have added these results (Line 266) in Fig.4D and Supplementary Fig. S5B in the revised manuscript .

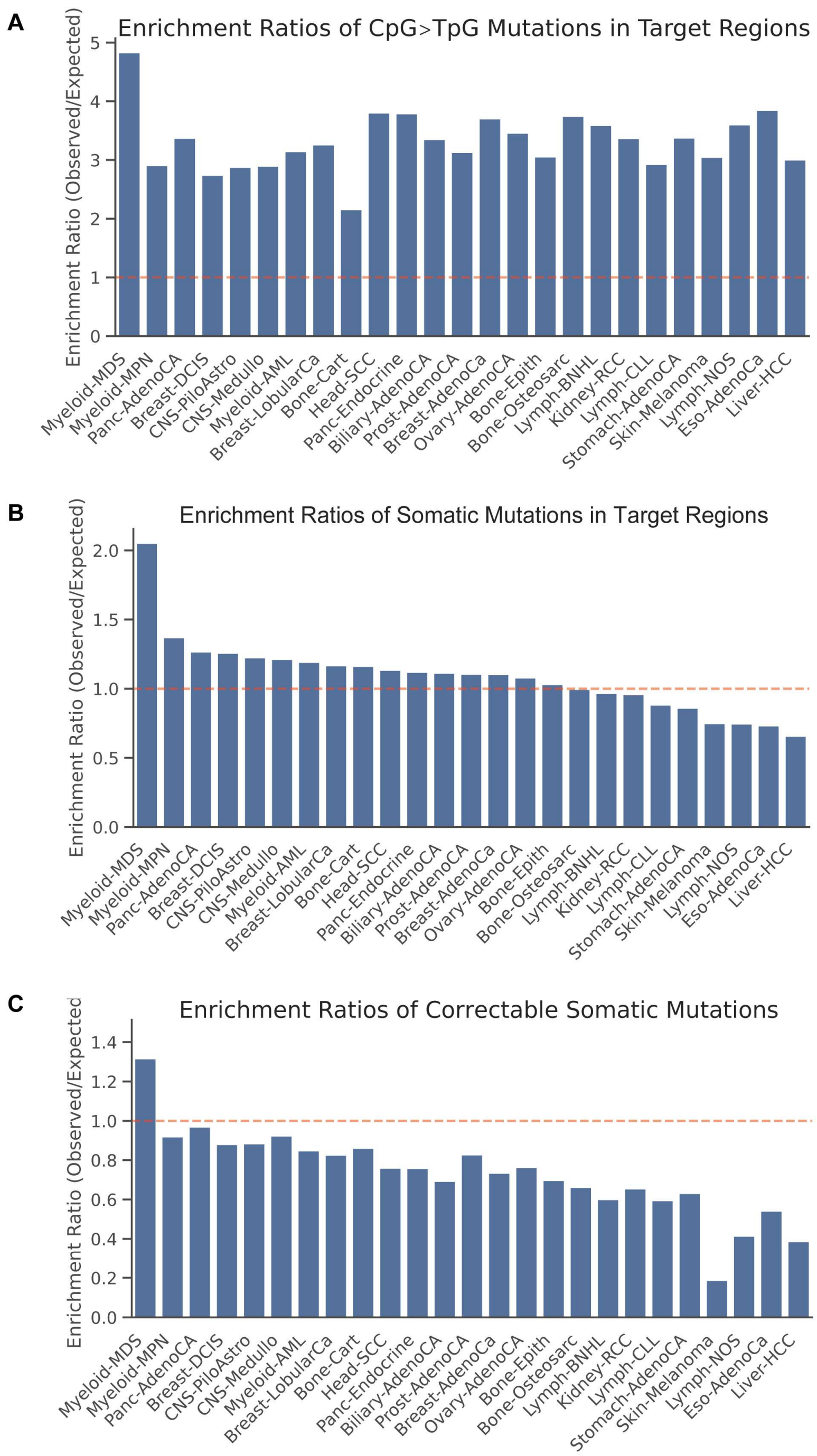

Figure R5. Enrichment ratios of different mutations in MspI-RRS target regions. Enrichment ratios were calculated as the normalized mutation burden in MspI-RRS regions divided by that in the whole genome. **A.** Enrichment ratios of CpG>TpG mutations across tumor types. **B.** Enrichment ratios of total somatic mutation burden across tumor types. **C.** Enrichment ratios of correctable somatic mutations across tumor types. A ratio of 1 is indicated by the dashed line. Data were derived from WGS analysis of the ICGC database.

R2c5&c10. The report lacks standardized benchmarking for ctDNA detection. Specifically, it presents no experimental comparison with commercially available ctDNA assays or other established research-grade ultrasensitive methods. Furthermore, the in silico mixing validation used is insufficient, as it cannot substitute for real-world limit-of-detection studies performed with spike-in controls.

Perform direct comparison with at least one established duplex sequencing method using the same samples. Benchmark against a clinically validated ctDNA assay to establish real-world sensitivity/specificity.

Response: We thank the reviewer for pointing this out. Following your suggestion, we performed a spike-in experiment to compare MASD-seq with a commercial hybrid-capture-based targeted sequencing assay. This assay employs the iGenetech TargetSeq® Solid Tumor Mid Panel, which covers 438.3 kb of solid-tumor-related genomic regions. The assay incorporates 64 synthetic sequences as unique molecular identifiers (UMIs) at the adaptors, enabling both single-strand consensus (SSC) and double-strand consensus (DSC) analysis for ultrasensitive duplex sequencing. DSCs are generated following SSC construction by pairing reverse-complement UMI1/UMI2 combinations from opposite strands.

Genomic DNA (gDNA) from the tumor tissue samples of Patient 1 was mixed into 60 ng of WBC gDNA from a normal individual at tumor mutant allele fractions of 0.025%, 0.0125%, 0.0063%, and 0%, with replicates for each group (Fig. R6A). For MASD-seq, each sample was sequenced to 100 Gb of raw data; for the hybrid-capture assay, each sample was sequenced to 30 Gb of raw data to achieve >20,000× depth for saturation. To enable a fair comparison, the MASD-seq data were downsampled to match the raw data input of the hybrid-capture assay. For both methods, tumor mutations were identified based on WGS data from the paired tumor and WBC samples of Patient 1, with 100 Gb of raw data generated per sample.

The results showed that SSC exhibited a background of 0.53×10^{-4} and was therefore unable to clearly distinguish the 0.0063% fraction. MASD-seq and DSC exhibited a background of 3.6×10^{-6} and 0, respectively and were able to detect the 0.0063% fraction. However, due to the relatively low recovery rate of DSC, DSC detected only an average of 3, 1.5, and 0.5 mutation alleles for the 0.025%, 0.0125% and 0.0063% fractions, respectively (Fig. R6B). In comparison, after downsampling MASD-seq data to the same amount of raw data as the hybrid-capture assay, MASD-seq detected an average of 39.5, 21.5 and 12 mutation alleles for the 0.025%, 0.0125% and 0.0063% fractions, respectively (Fig. R6B). MASD-seq exhibited markedly higher signal-to-noise ratios than DSC and SSC (Fig. R6C).

Together, these results demonstrate the superior performance of MASD-seq compared to both SSC and DSC approaches. We have added these results (Line 353) to Fig. 6B, Fig. S7A, Table EV4, and the Methods section (Line 813) in the revised manuscript.

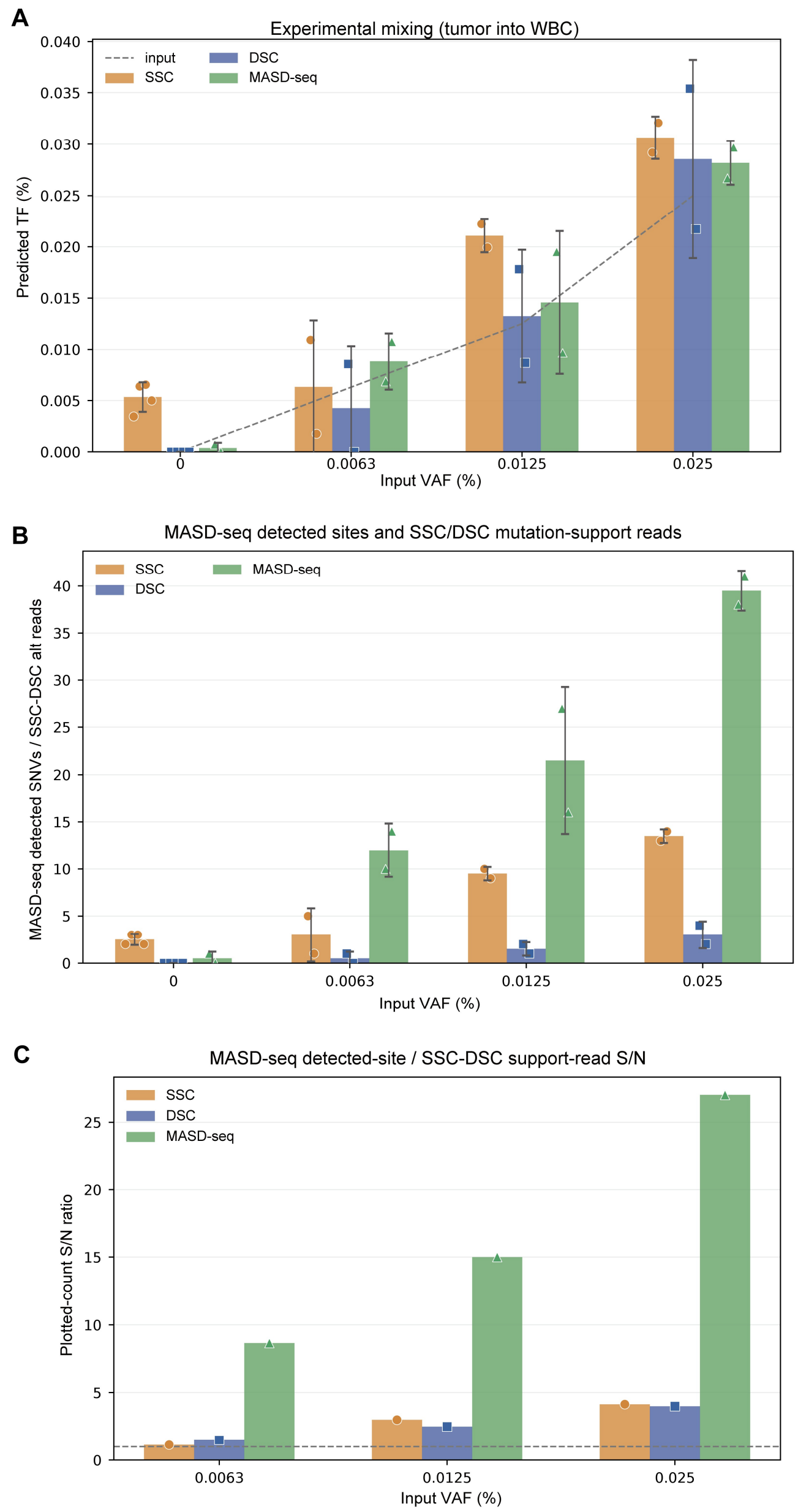

Figure R6. Performance comparison of MASD-seq,single-strand consensus (SSC) and double-strand consensus (DSC) in detecting low-abundance mutant alleles. Tumor gDNA from Patient 1 was mixed into WBC gDNA from a healthy individual at expected VAFs of 0.025%, 0.0125%, 0.0063%, and 0%. MASD-seq was performed in comparison with a commercial hybrid-capture-based targeted sequencing assay that supports both SSC and DSC analysis. **A.** Predicted TFs for the three approaches are shown, with the dashed line indicating the expected input VAF and bars showing mean values ± SD. **B.** Detected mutation signals for the three approaches are shown. MASD-seq signals represent detected mutant sites, whereas SSC and DSC signals represent mutation-supporting reads (alt reads). Bars show mean values ± SD**. C.** Signal-to-noise ratios of detected mutation signals are shown, calculated using the 0% fraction as background.

R2c6. Regarding figure and supplementary material clarity. Some figures (e.g., Fig. S1, S3) are overly complex and not clearly explained in the main text.

Response: We thank the reviewer for this feedback. We have clarified Figs. S1 and S3 and relocated them to the main figures (see Fig. R7 and Fig. R8). Corresponding explanations have also been expanded and clarified in the main text.

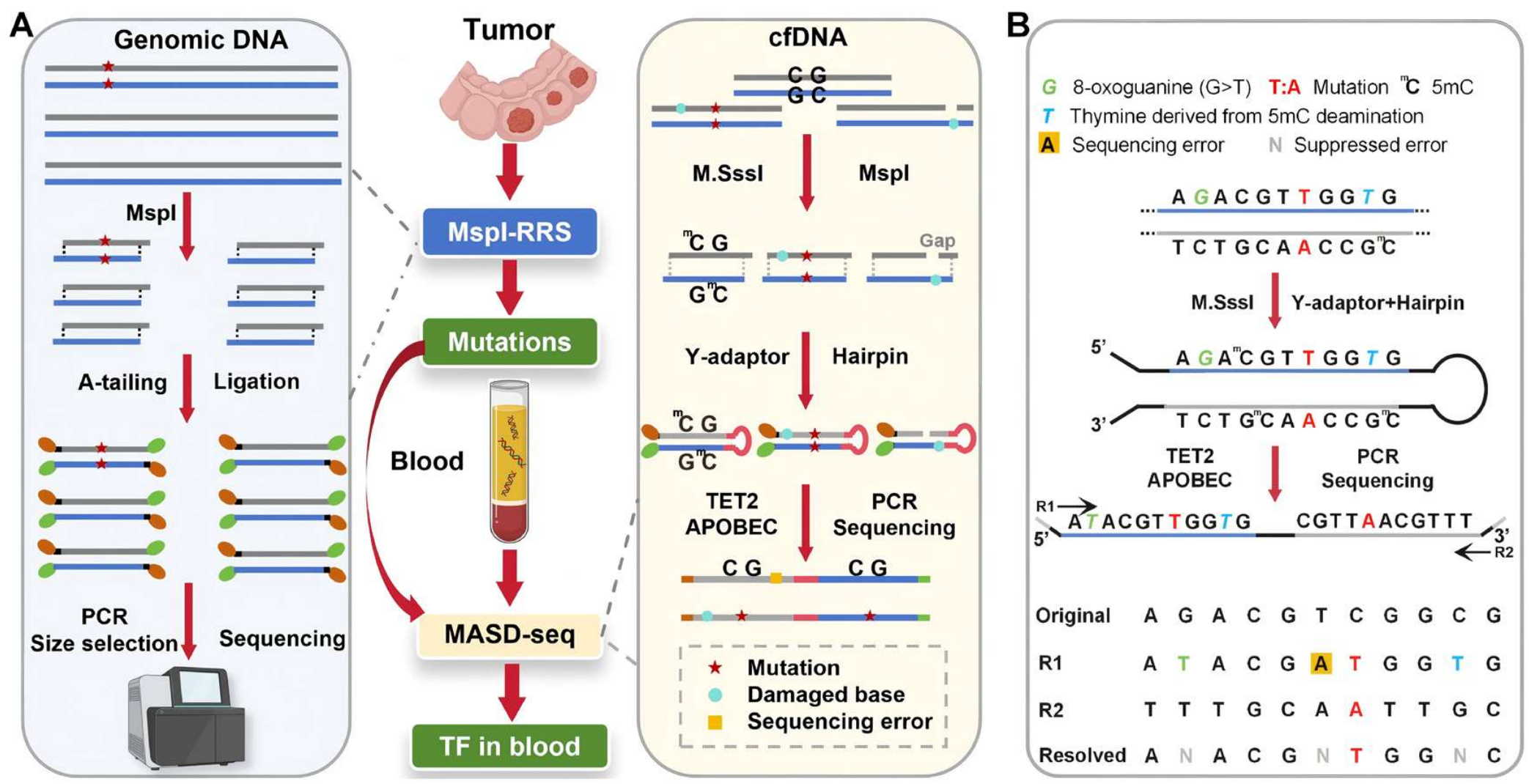

Figure R7 (Fig. 1 in the revised manuscript). Workflow schematic for MASD-seq and MspI-RRS. A. For tissue samples, a RRS assay using the restriction enzyme MspI (recognizing C|CGG, MspI-RRS) is performed to identify tumor-specific mutations (left panel). The MspI digestion enriches for both the CpG>TpG mutation burden and the total mutation burden while reducing genome complexity. For plasma cfDNAs, MASD-seq that enables efficient single-read error correction through physically linking the original DNA double strands, is employed to quantify the tumor fraction (TF, which is the ratio of mutant alleles to the total number of alleles) in blood (right panel). **B.** The MASD-seq involves three main steps: (1) following MspI digestion, hairpin adapters are ligated to double-stranded DNA; (2) CpG methyltransferase (M.SssI) methylates all CpG sites; and (3) TET2 and APOBEC/UvrD helicase converts unmethylated cytosines to uracil to enable PCR amplification and sequencing. Physical linkage of Watson-Crick strands via hairpin adapters enables error correction in a single-duplex read. Since all cytosines in CpG dinucleotides remain methylated (and thus protected), the method enables correction of the CpG>TpG error, as well as all transversion errors (C>A, C>G, T>G, and T>A). The resolve rules dictated that non-permissible pairs, such as TpG-CpG, T-T and A-A, were masked (marked as N) at the affected position.

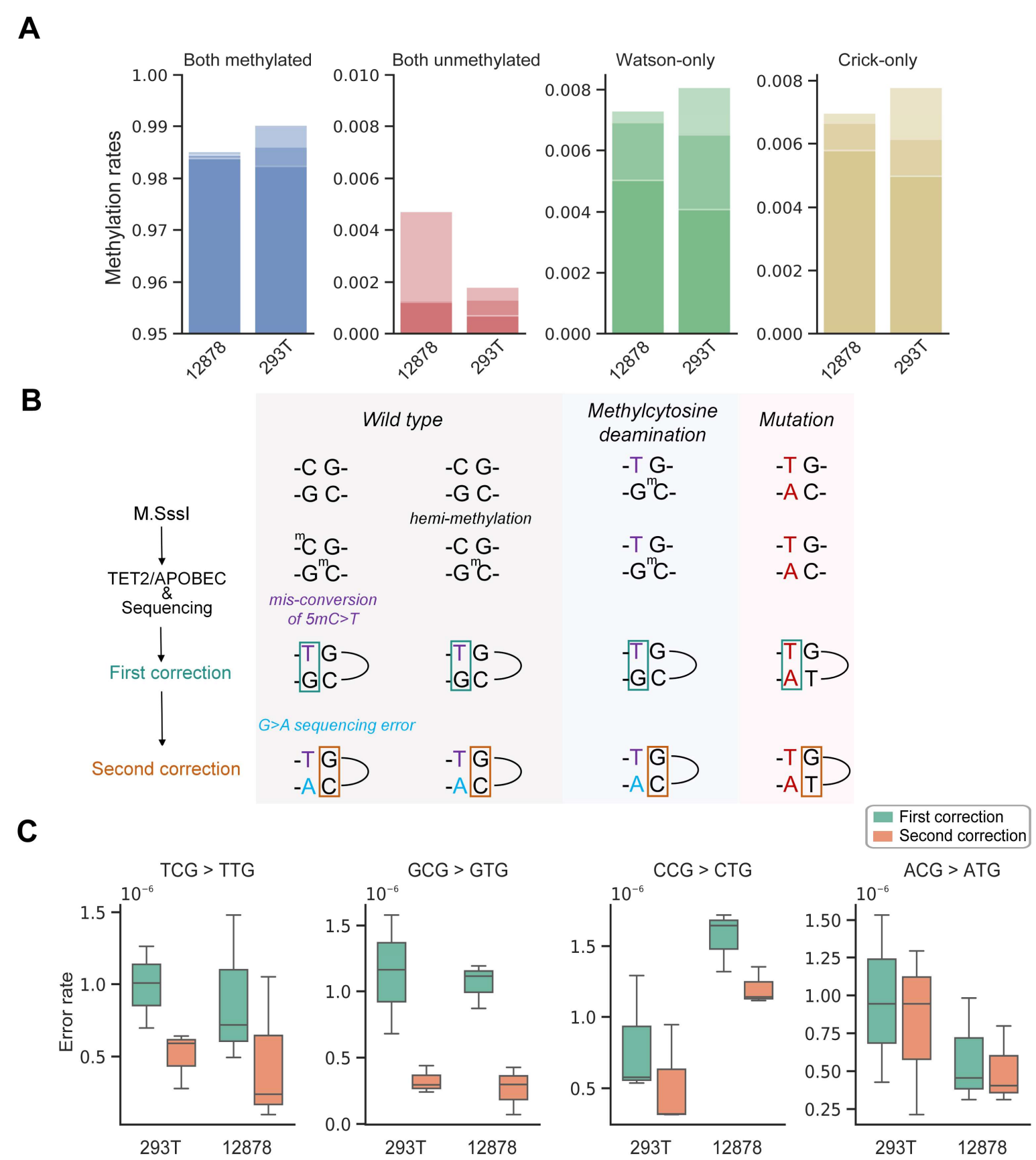

Figure R8 (Fig.2 in the revised manuscript). MASD-seq accuracy. A. DNA methylation status in GM12878 and HEK293T cell lines following methyltransferase treatment. Four patterns are shown: methylated on both strands, unmethylated on both strands, and methylated on single-strand. Different shades represent three independent replicates. **B.** Schematic of the double-CpG correction algorithm. Three scenarios are illustrated: wild-type CpG sites, methylcytosine deamination, and genuine mutation. In wild-type molecules, false-positive CpG>TpG signals may arise from incomplete protection or mis-conversion of 5mC to T, as well as from hemi-methylated CpG sites. After M.SssI treatment, the complementary cytosine at a hemi-methylated CpG can be methylated and protected from TET2/APOBEC conversion. In the First correction step, these false-positive events are identified by the presence of a retained CpG dinucleotide on the complementary strand and are distinguished from true CpG>TpG mutations. In contrast, a genuine mutation permanently disrupts the CpG dinucleotide, preventing M.SssI-mediated methylation and resulting in loss of the CpG signal on both strands. The Second correction step further removes sequencing errors, such as G>A artifacts, by checking whether the complementary strand still contains a protected cytosine. If a retained C is detected on the complementary strand, the apparent mutation is classified as an error and filtered out. **C.** Detected CpG>TpG mutation frequencies in different contexts for both cell lines before (green) and after (orange) using the double correction algorithm.

R2c7. Regarding methodological details insufficient for replication. The MASD-seq protocol involves multiple enzymatic steps (Mspl, M.SssI, TET2, APOBEC3A, UvrD). Critical parameters (reaction efficiencies, enzyme lot variations, optimization steps) are not fully detailed.

Response: We thank the reviewer for this feedback. To enhance reproducibility of the method, we have added a detailed, step-by-step protocol in the Appendix PDF.

A key new step of MASD-seq is the use of M.SssI to premethylate CpG sites. To optimize this step, we performed experiments to investigate the reaction time and enzyme concentration, and selected 1 h and 8 U as the optimized condition. For the TET2 and APOBEC3A enzymes, which are included in the commercially available EM-seq kit, we adopted the manufacturer's recommended conditions, assuming they have been optimized by the supplier. Similarly, we did not independently optimize the UvrD reaction conditions, but instead followed the protocols reported in the established five-letter sequencing methodology. We have provided key performance metrics, including the CG methylation rate and duplication rate in the Table EV1 of the revised manuscript.

R2c8. Regarding statistical and analytical rigor. The use of Pearson correlation for mutation burden comparison (Fig. 3E) may not be appropriate for non-normally distributed count data. No multiple testing correction applied in mutation burden analyses.

Response: We have made these revisions. In the revised manuscript, Spearman's rank correlation ($\rho = 0.79$) is now presented in the Fig. 5C (Line 306), and the results of multiple testing correction are shown in the Fig.5B.

R2c9. The abstract claims "highly accurate sequencing method and holds promise for clinical applications", but the clinical data (clinical models) are preliminary.

Response: We thank the reviewer for this feedback. We have revised the sentence as follows: " These findings demonstrate that MASD-seq is a highly accurate sequencing method, suggesting its potential for future clinical applications."

R2c10. **Response:** We have addressed this point in our response to R2c5&c10.

R2c11. Clarify limitations regarding uncorrectable mutations and tumor types where MASD-seq may not be optimal.

Response: We have clarified limitations regarding uncorrectable mutations and tumor types where MASD-seq may or may not be optimal in the Discussion (Line 563) as follows: "Our results showed that the CpG>TpG mutation burden and tumor mutation enrichment by MspI-RRS vary across cancer types. CRC ranks among the highest in both metrics. For other cancer types, we propose that MASD-seq is expected to achieve relatively high efficiency in types with a correctable mutation ratio higher than 0.9, such as pancreatic adenocarcinoma, which exhibits values of 3.3, 1.28, and 0.97 for the CpG>TpG enrichment, the total mutation burden enrichment, and the correctable mutation ratio, respectively. For cancer types with slightly lower enrichment metrics, such as prostate adenocarcinoma (CpG>TpG enrichment: 3.0; total mutation burden enrichment: 1.11; correctable mutation ratio: 0.83), MASD-seq would still be acceptable. Due to the limitation of uncorrectable mutations, the approach is less suitable for tumors with low CpG>TpG or high CH>TH/T>C burdens, such as liver cancer, kidney cancer, and melanoma. For lung cancer, although the CpG>TpG burden is low, an EM-seq-based hairpin strategy without MspI-digestion

may still be applicable, as the mutational spectrum of lung cancer is dominated by C>A transversions, which is correctable by the strategy."

R2c12. Provide more detailed protocols in the Methods section to ensure reproducibility. Tone down overstated claims in the abstract and discussion regarding clinical readiness.

Response: We thank the reviewer for this feedback. we have provided a detailed, step-by-step protocol in the Appendix PDF to ensure reproducibility in the revised manuscript. We have also toned down overstated claims in the abstract and discussion (Line 592) regarding clinical readiness. In the abstract, we have made the revision as "suggesting its potential for future clinical applications ".

Response to Referee #3

Referee #3 (Comments on Novelty/Model System for Author):

From a technical standpoint, this strategy is novel and validated using serval cohorts. However, the authors validate their method only on colorectal cancer samples. To assess broader clinical applicability, it is necessary to evaluate the method on samples from other cancer types and to determine whether it introduces bias in other mutational signatures, given that different types of substitutions are corrected differently by their method. More technical details are required to evaluate the validity of the method.

The authors describe MASD-seq, a sequencing strategy integrating duplex consensus correction and CpG-targeted reduced representation sequencing for sensitive somatic mutation detection. The study addresses an important methodological challenge and is supported by validation in cell lines, clinical colorectal cancer samples, and large external datasets.

Response: We sincerely thank the reviewer for the positive and constructive comments.

R3C1. Evidence supporting the biological enrichment of CpG>TpG mutations is primarily presented in the supplementary material (e.g. Supplementary Figure S6). Given the centrality of this point to the manuscript's main claims, clearer integration of these results into the main text or figures would strengthen the narrative and improve accessibility for readers.

Response: We thank the reviewer for this helpful comment. In our previous manuscript, we mainly focused on colorectal cancer, as it has been reported to be highly enriched for CpG>TpG mutations, and our primary research area is gastrointestinal cancers. We have performed additional analyses on other cancer types, including analysis of the IGCG database as described in the comments and experimental analysis of two other cancer types as detailed in the following comments.

Our analysis of the IGCG database showed that the MspI region enriches CpG>TpG mutations by 1.9- to 4.76-fold across different cancer types (Fig. R9A). Furthermore, this region enriches the total mutation burden by 0.66- to 2.05-fold, with 16 out of 25 cancer types exhibiting a fold enrichment greater than 1 (Fig. R9B). Finally, when considering the correctable mutation burden, myelodysplastic syndromes (MDS), myeloproliferative neoplasms (MPN), pancreatic adenocarcinoma, and medulloblastoma exhibited an enrichment ratio higher than 0.9, with values of 1.32, 0.92, 0.97, and 0.92, respectively (Fig. R9C). Five cancer types including prostate adenocarcinoma, acute myeloid leukemia (AML), and breast invasive lobular carcinoma exhibited an enrichment ratio between 0.8 and 0.9 (Fig. R9C). In comparison, the CpG>TpG enrichment ratio, total burden enrichment, and correctable mutation burden ratio were 4, 1.6, and 1.14, respectively, for MSH-I CRC, and 3, 1.2, and 0.95 for MSS CRC. These findings showed that the mutation burden enrichment in the MspI-region varies across human tumor types, with CRC being among the cancer types with the highest enrichment.

Based on these observations, we propose that MASD-seq is expected to achieve relatively high efficiency in cancer types with a correctable mutation ratio higher than 0.9, particularly pancreatic adenocarcinoma, which exhibits values of 3.3, 1.28, and 0.97 for the CpG>TpG enrichment, the total mutation burden enrichment, and the correctable mutation ratio, respectively. For cancer types with slightly lower enrichment metrics, such as prostate adenocarcinoma (CpG>TpG enrichment: 3.0; total mutation burden enrichment: 1.11; correctable mutation ratio: 0.83), MASD-seq would still yield acceptable efficiency. For cancers with a low CpG>TpG burden, such as lung cancer, CpG>TpG enrichment is not necessary. However, alternative EM-seq-based hairpin strategies may be suitable, as the mutational spectrum of lung cancer is dominated by C>A transversions, which is correctable by the approach. We have added these results in Fig. 4D and Fig. S5B in the revised manuscript (Line 266).

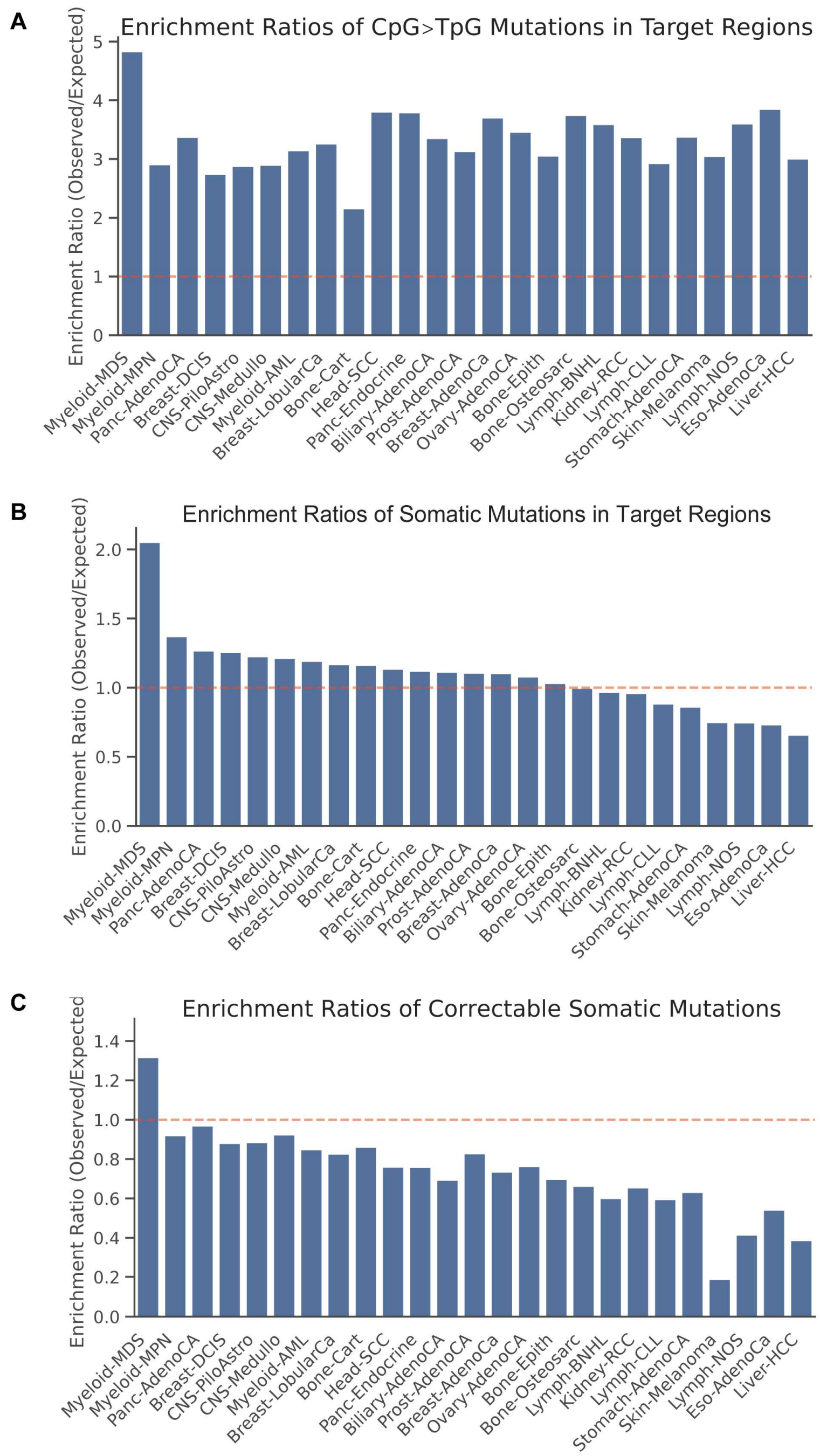

Figure R9. Enrichment ratios of different mutations in MspI-RRS target regions. Enrichment ratios were calculated as the normalized mutation burden in MspI-RRS regions divided by that in the whole genome. **A.** Enrichment ratios of CpG>TpG mutations across tumor types. **B.** Enrichment ratios of total somatic mutation burden across tumor types. **C.** Enrichment ratios of correctable somatic mutations across tumor types. A ratio of 1, indicated by the dashed line. Data were derived from WGS analysis of the ICGC database.

R3C2. The manuscript supports the cost-efficiency of MASD-seq and MspI-RRS through sequencing depth usage, duplication rates, and data volume comparisons relative to WGS. However, these points are largely presented late in the Discussion and are not clearly summarized in the Results. Explicitly presenting sequencing depth or data volume comparisons in the main results (e.g. via a summary table or schematic) would strengthen and clarify this claim.

Response: We thank the reviewer for pointing this out. In the revised manuscript, we have provided the raw sequencing data with detailed depths of MspI-RRS for 31 CRC tumor tissue in the main text (Line 292) and Table EV2. Specifically, the tumor tissue samples were sequenced to an average of 23 Gb of raw data, achieving a median depth of 51× for the MspI-RRS region.

In comparison, WGS generally requires 100 Gb for the tumor tissue to reach approximately 30× depth. In the spike-in experiment, we also performed WGS for Patient 1, with the tumor tissue samples being sequenced to 132 Gb of raw data and achieved a mean depth of 29. A comparison between mutations derived from WGS and MspI-RRS showed that, despite the substantially lower raw sequencing data of MspI-RRS compared with WGS for the tumor tissue (19.2 Gb for MspI-RRS vs. 132 Gb for WGS), the two methods yielded similar signal-to-noise ratios and detected comparable tumor mutations at each dilution fraction (Fig. R10, and also Fig. S7B in the revised manuscript). Together, these results support our conclusion that MspI-RRS offers more than a five-fold improvement in cost efficiency compared to WGS.

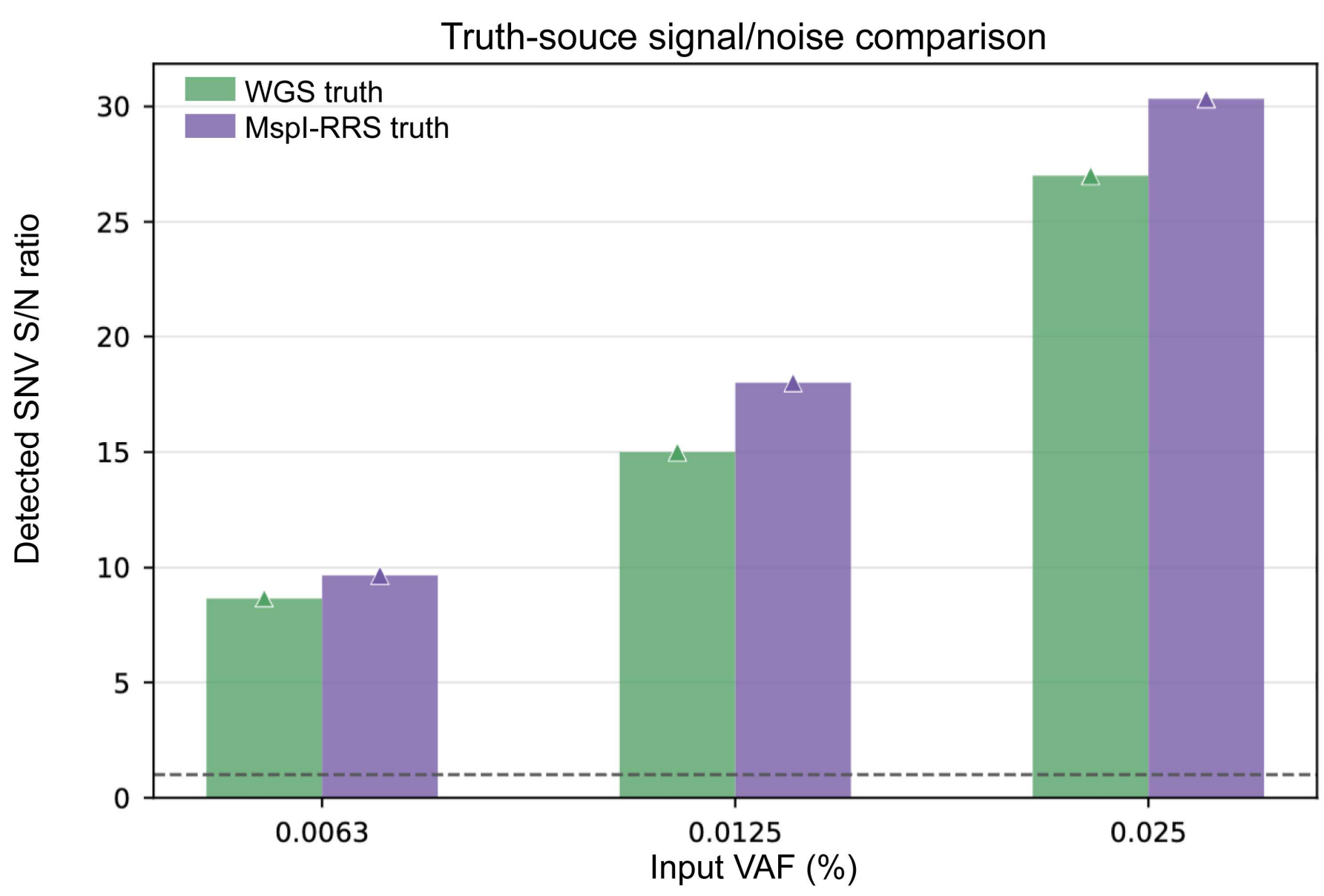

Figure R10. Comparison of SNV detection between WGS and MspI-RRS. The signal-to-noise ratios for SNV detection from WGS-derived and MspI-RRS-derived mutations are shown for each spike-in dilution fraction (0.0063%, 0.0125%, and 0.025%).

R3C3. The benefit of duplex consensus correction is convincingly demonstrated in cell line models; however, its contribution to improved accuracy in primary tumor tissue or cfDNA samples is less explicitly shown. Clearer contextualization of this limitation, or additional clarification of how duplex correction impacts clinical samples, would improve interpretation.

Response: We thank the reviewer for pointing this out. To address the question, we performed a spike-in experiment to compare MASD-seq with a commercial hybrid-capture-based targeted sequencing assay. This assay employs the iGenetech TargetSeq® Solid Tumor Mid Panel, which covers 438.3 kb of solid-tumor-related genomic regions. The assay incorporates 64 synthetic sequences as unique molecular identifiers (UMIs) at the adaptors, enabling both single-strand consensus (SSC) and double-strand consensus (DSC) analysis for ultrasensitive duplex sequencing. DSCs are generated following SSC construction by pairing reverse-complement UMI1/UMI2 combinations from opposite strands.

Genomic DNA (gDNA) from the tumor tissue samples of Patient 1 was mixed into 60 ng of WBC gDNA from a normal individual at tumor mutant allele fractions of 0.025%, 0.0125%, 0.0063%, and 0%, with replicates for each group. For MASD-seq, each sample was sequenced to 100 Gb of raw data; for the hybrid-capture assay, each sample was sequenced to 30 Gb of raw data to achieve >20,000× depth for saturation. To enable a fair comparison, the MASD-seq data were downsampled to match the raw data input of the hybrid-capture assay. For both methods, tumor mutations were identified based on WGS data from the paired tumor and WBC samples of Patient 1, with 100 Gb of raw data generated per sample.

The results showed that SSC exhibited a background of 0.53×10^{-4}, which precluded clear detection of the 0.0063% fraction. In contrast, MASD-seq and DSC showed substantially lower backgrounds of 3.6×10^{-6} and 0, respectively, and both successfully detected the 0.0063% fraction (Fig. R11A). However, despite its high sensitivity, DSC had a relatively low recovery rate, detecting an average of only 3, 1.5, and 0.5 mutant alleles for the 0.025%, 0.0125% and 0.0063% fractions, respectively. By comparison, with matched raw data, MASD-seq detected an average of 39.5, 21.5 and 12 mutant alleles for the 0.025%, 0.0125% and 0.0063% fractions, respectively (Fig. R11B). Consequently, MASD-seq achieved markedly higher signal-to-noise ratios than both DSC and SSC (Fig. R11C). Together, these results demonstrate the superior performance of MASD-seq compared to both SSC and DSC approaches. We have added these results to Fig. 6B, Fig. S7A, Table EV4 (Line 354), and the Methods section (Line 813) in the revised manuscript.

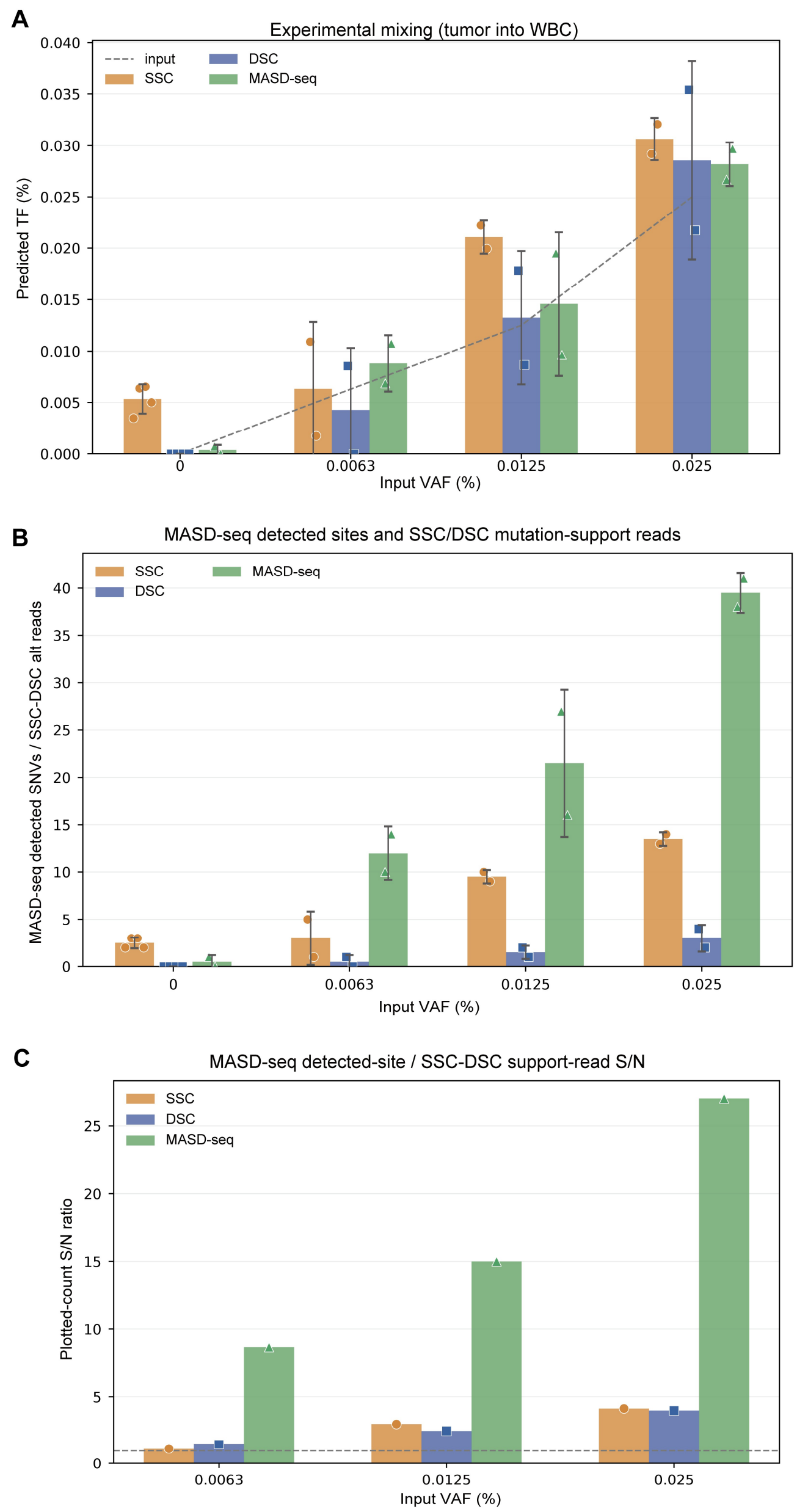

Figure R11. Performance comparison of MASD-seq,single-strand consensus (SSC) and double-strand consensus (DSC) in detecting low-abundance mutant alleles. Tumor gDNA from Patient 1 was mixed into WBC gDNA from a healthy individual at expected VAFs of 0.025%, 0.0125%, 0.0063%, and 0%. MASD-seq was performed in comparison with a commercial hybrid-capture-based targeted sequencing assay that supports both SSC and DSC analysis. **A.** Predicted TFs for the three approaches are shown, with the dashed line indicating the expected input VAF and bars showing mean values ± SD. **B.** Detected mutation signals for the three approaches are shown. MASD-seq signals represent detected mutant sites, whereas SSC and DSC signals represent mutation-supporting reads (alt reads). Bars show mean values ± SD**. C.** Signal-to-noise ratios of detected mutation signals are shown, calculated using the 0% fraction as background.

R3C4. The selected cell lines (GM12878 and HEK293T) are appropriate for technical validation of error correction but have limited relevance to tumour biology. Explicitly distinguishing technical validation from biological interpretation would improve clarity.

Response: We have acknowledged that technical validation of error correction in cell lines does not fully reflect the biological complexity of clinical tumor detection. Therefore, after validating MASD-seq in GM12878 and HEK293T cell lines, we applied the method to plasma cfDNA from healthy individuals and colorectal cancer (CRC) patients.

Analysis of healthy-individual cfDNA revealed a positive correlation between age and mutation burden for correctable mutation types, confirming the high accuracy of MASD-seq in human biological samples. These results also indicate that, for clinical ctDNA detection, the background noise is not dominated by sequencing errors, but rather by age-related somatic mutation burden. Specifically, the background rates were 9.4×10^{-7} for CpG>TpG mutations and $5.6 \times 10^{-8}$for transversion mutations. In the Discussion, we proposed that Given that CpG>TpG and transversion mutations account for approximately 60% and 40% of all correctable mutations in the MspI-RRS region in CRC, respectively, MASD-seq achieves an overall background error rate of approximately 6×10^{-7} for ctDNA detection.

We further evaluated MASD-seq performance in a real-world clinical tumor detection setting by analyzing plasma cfDNA from CRC patients before and after surgery. The pre-surgery samples yielded an area under the curve (AUC) of 0.97 for ctDNA detection, demonstrating high diagnostic accuracy. Moreover, comparison of paired pre- and post-surgery samples from the same patients further validated the authenticity of the detected tumor signals, as mutation burdens decreased significantly after tumor resection.

We have made some revisions to the manuscript to better distinguish technical validation from biological interpretation. In the Abstract, we have specified that the technical error rate was derived from cell lines; in the Discussion (Line 503), we have provided a clearer expression that "the background is therefore not dominated by sequencing errors, but rather by the age-related somatic mutation burden."

R3C5. To assess broader clinical applicability, it is necessary to evaluate the method on samples from other cancer types and to determine whether it introduces bias in other mutational signatures, given that different types of substitutions are corrected differently by the method.

Response: To examine whether the MspI-RRS approach enriches for tumor mutations in other cancer types, we applied MspI-RRS to gastric cancer (GC; n = 7) and hepatocellular carcinoma (HCC; n = 5) samples, in comparison with HindIII/XbaI-RRS. The results showed that, the MspI-targeted region exhibited consistent enrichment of CpG>TpG mutations compared with the HindIII/XbaI-targeted regions in both GC and HCC. The median mutation burden increased from 0.056 to 0.716 per Mb in GC and from 0.111 to 0.751 per Mb in HCC (Fig. R12). In GC, while the total mutation burden was higher in the MspI region than in the HindIII/XbaI region, the corrected mutation burden in the MspI region was lower than the total burden in the HindIII/XbaI region; however, neither difference was statistically significant. In HCC, the total mutation burden was

comparable between the two regions, whereas the corrected mutation burden in the MspI region was markedly lower than the total burden in the HindIII/XbaI region (Fig. R12). Together, these results showed that GC is more amenable to MspI-RRS enrichment than HCC. Additional GC data are warranted to assess this in light of intertumor heterogeneity and potential population-specific differences in mutational signatures. We have integrated the data into the Results (Line 316), Table EV3, and Fig. S6 in the revised manuscript.

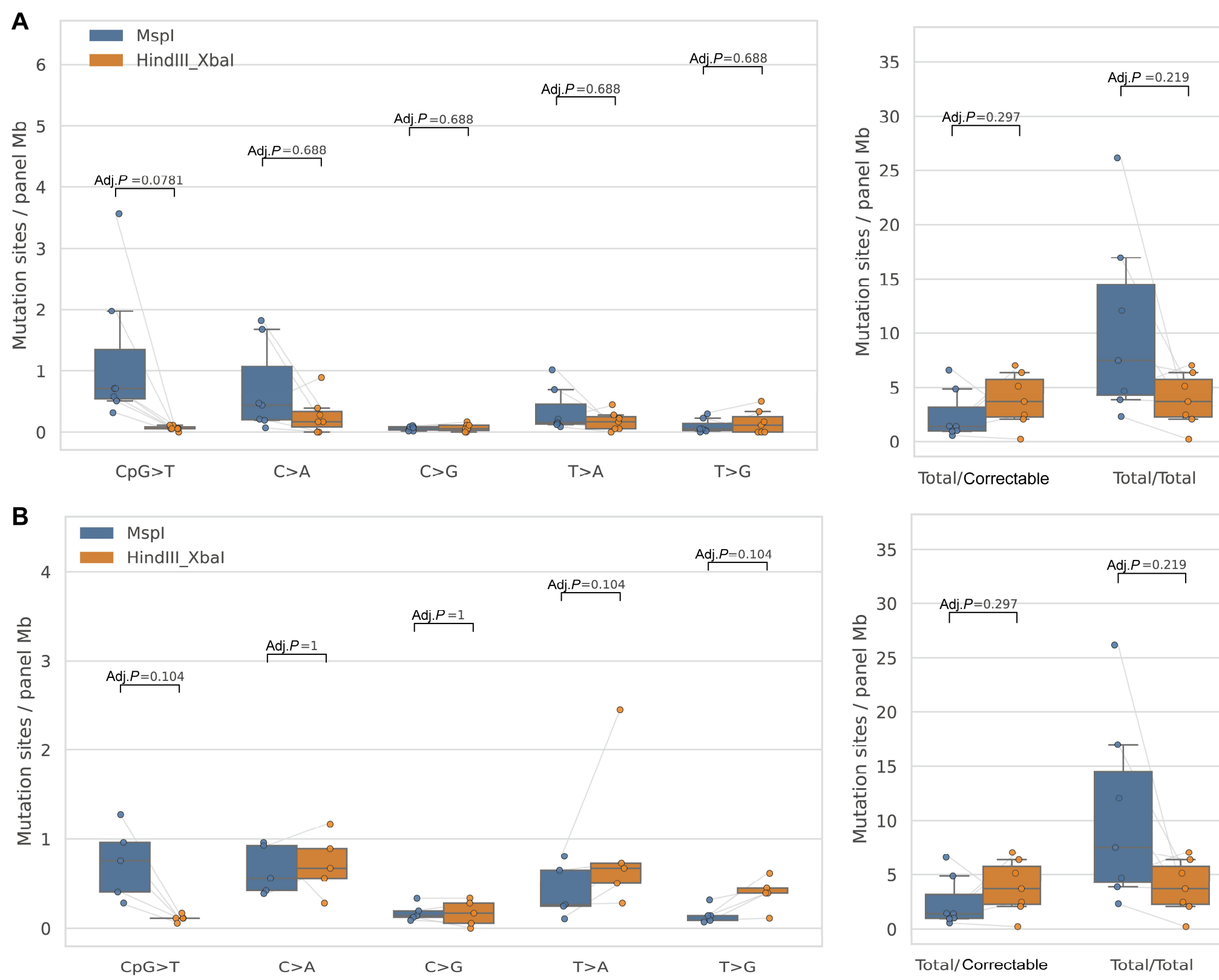

Figure R12. Tumor mutation burden in the MspI region versus HindIII/XbaI region in GC and HCC. A. Mutation burden comparison in GC. Left panel: distribution of different mutation classes. Right panel: comparison of corrected burden (MspI) versus total burden (HindIII/XbaI), and total mutation burdens between the two regions. **B.** Mutation burden comparison in HCC. Left panel: distribution of different mutation classes. Right panel: comparison of corrected burden (MspI) versus total burden (HindIII/XbaI), and total mutation burdens between the two regions. Bars indicate mean values, error bars indicate standard deviation, and dots represent individual samples.

R3C6. There is no explanation about how the error rate is calculated, despite it being a fundamental concept underlying the proposed methodology. In the absence of this explanation, it is not possible to evaluate the validity of the method.

Response: Thank you for this comment. We calculate context-specific error rates using a systematic framework that consists of three key components:

1. Germline-filtered high-confidence pileup generation
2. Trinucleotide context-specific aggregation
3. Mathematical calculation of error rates

First, pileup generation and filtering: We generate germline-filtered, high-confidence pileups from aligned sequencing reads using stringent quality filters including: base quality ≥20 (Phred scale), mapping quality ≥30, and read-level filters to remove PCR duplicates and low-complexity reads. Candidate variants that overlap known germline variants (from gnomAD, 1000 Genomes, or matched normal samples) are excluded to prevent inflation of sequencing errors. This germline filtering ensures that downstream calculations use only true sequencing errors.

Second, trinucleotide context aggregation: For each genomic position, we define its trinucleotide context using the reference genome. The context is represented by the sequence 5'–N1–[Central Base]–N2–3', where the central base corresponds to the pileup reference base. Because each position has 4 possibilities for the 5' base, 4 for the central base, and 4 for the 3' base, there are $4^3= 64$ possible trinucleotide contexts. For each context, we aggregate observations across all positions sharing the same trinucleotide context. For each central reference base and each possible alternate base, we sum the counts across all genomic positions with the identical context, yielding the total number of alternate observations (N_{alt}) and the effective number of observations (D_{eff}).

Third, context-specific error rate calculation: For each trinucleotide context and each alternate base, the error rate is calculated as:

$$E(\text{context, alt})=\frac{N_{alt}}{D_{eff}}$$

where:

- E(context, alt): error rate for a given trinucleotide context and alternate base
- N_{alt}: count of observations of the specified alternate base at sites with the given context (reference base excluded)
- D_{eff}: effective pileup depth at sites with the given context, defined as the sum of all observations (reference and alternate bases, A, C, G, T) at the central position

This formulation yields the probability that a given nucleotide position with a specific trinucleotide context contains an error. Critically, the denominator includes all base observations (both reference-matching and non-reference bases), ensuring that the error rate is conditioned on the true depth of observation; however, the numerator excludes the reference base itself, as the reference base by definition cannot contribute to sequencing errors at that position.

Optional adjustment for known biochemical conversions: For an adjusted analysis, we mask putative deamination-derived conversions using base modification information. Specifically, C-to-T substitutions in a CpG context (5'–C–pG–3') and their reciprocal G-to-A substitutions in a GpC context (5'–G–pC–3') are masked based on base-modification flags in the aligned BAM file. These masked bases are excluded from both the numerator and denominator of error rate calculations in the adjusted analysis.

A detailed description of these steps and the associated filtering criteria is provided in the Methods

section (Line 676) of the revised manuscript.

20th Jul 2026

Dear Dr. Wen,

Thank you for submitting the revised version of your manuscript. We have now received the reports from the three referees who re-evaluated your study. As you will see from the comments below, the referees are generally satisfied with the revisions you have made. However, Referee #3 has raised a few remaining concerns. We therefore ask that you provide a point-by-point response to these comments and, where appropriate, moderate your conclusions accordingly.

On a more editorial level:

1. Please remove all figures from manuscript file. The figure legends should stay in the manuscript file and should be placed at the end of manuscript.

2. Please remove the "Author contributions" section from the manuscript file.

3. Appendix: Please add page numbers to the table of contents and correct the nomenclature to Appendix Figure S1 etc. and Appendix Supplementary Protocol.

4. Appendix Figure S6: Please carefully check the combined mutation class plots on the right, as the panels in A and B appear to be identical. Please correct them if this is an error.

5. Please move "the paper explained" to the manuscript file.

6. Please incorporate the information currently provided in the Ethics Statement into the Methods section at the appropriate locations. The separate Ethics Statement section should then be removed.
For the human studies, please also include a statement that the experiments conformed to the principles set out in the WMA Declaration of Helsinki and the Department of Health and Human Services Belmont Report.

7. We have recently updated the size requirements for the synopsis image. To ensure optimal display on the website, please submit a revised version as a PNG file with dimensions of exactly 550 × 300 pixels (width × height). We apologize for the inconvenience.

8. Please rename Tables EV1-EV5 as Datasets EV1-EV5, as they contain complex data. For each dataset, please include a legend in a separate tab/worksheet.
Please rename Table EV6 as Table EV1 and add a short legend at the top of the table. Please upload each EV dataset and table as a separate file.

9. Please address the following issues in figure legends:
- Please indicate the statistical test used for data analysis in thelegends of figures 3B, 4C, 5B
-Please note that the box plots need to be defined in terms of minima, maxima, centre, bounds of box and whiskers, and percentile in the legends of figures 2C, 3A, 5B
-Please note that information related to n is missing in the legends of figures 2C,4B, C; 6B, D; 7C
-Please note that the error bars are not defined in the legends of figures 4A-C; 6D.

10. Sections need to be named and the order should be corrected: Title page - Abstract - Introduction - Results - Discussion - Methods - Data Availability - Acknowledgements - Disclosure and Competing Interests Statement - References - Figure Legends - Table(s) - Expanded View Figure Legends.

I look forward to reading a new revised version of your manuscript as soon as possible.

Sincerely,
Jingyi

Jingyi Hou
Senior Editor
EMBO Molecular Medicine

*** Instructions to submit your revised manuscript ***

*** PLEASE NOTE *** As part of the EMBO Publications transparent editorial process initiative (see our Editorial at https://www.embopress.org/doi/pdf/10.1002/emmm.201000094), EMBO Molecular Medicine will publish online a Review Process File to accompany accepted manuscripts.

In the event of acceptance, this file will be published in conjunction with your paper and will include the anonymous referee reports, your point-by-point response and all pertinent correspondence relating to the manuscript. If you do NOT want this file to be published, please inform the editorial office at contact@embomolmed.org.

***** Reviewer's comments *****

Referee #1 (Comments on Novelty/Model System for Author):

N.A.

Referee #1 (Remarks for Author):

My concerns have been clearly addressed. Looking forward to reading the published version.

Referee #2 (Comments on Novelty/Model System for Author):

This manuscript presents an innovative method that substantially improves the detection accuracy of a major type and region of mutations. It elaborates the conceptual framework and validates the experimental design, clearly demonstrating its advantages and its promising clinical translational potential, especially in cancer detection.
This approach is expected to further advance the development of precision cancer medicine.

Referee #2 (Remarks for Author):

This manuscript presents an innovative method that substantially improves the detection accuracy of a major type and region of mutations. It elaborates the conceptual framework and validates the experimental design, clearly demonstrating its advantages and its promising clinical translational potential, especially in cancer detection.

Referee #3 (Comments on Novelty/Model System for Author):

The selected model systems are appropriate for the objectives of this study. The use of GM12878 and HEK293T cell lines provides a suitable platform for technical validation of sequencing accuracy and error correction, while colorectal cancer tissues and plasma cfDNA samples allow proof-of-principle evaluation of the method in a clinically relevant setting. The inclusion of publicly available datasets to assess mutation enrichment across multiple tumour types further strengthens the manuscript.

Nevertheless, the experimental validation of the complete MASD-seq workflow remains largely confined to colorectal cancer. Although the authors now discuss the applicability of the approach to additional cancer types and provide computational analyses of mutation enrichment, experimental validation in other tumour types is still limited. Future studies evaluating MASD-seq in independent cohorts representing different mutational landscapes would strengthen the evidence for broader clinical applicability.

Referee #3 (Remarks for Author):

I appreciate the authors' thorough revision and the effort invested in addressing my comments. The manuscript has been strengthened by the inclusion of additional methodological details, the comparison between EM-seq and bisulfite sequencing, the expanded figures, and the new benchmarking experiment against a duplex sequencing assay. Overall, the manuscript is improved.
However, I believe that two important issues remain only partially addressed.
1. Broader applicability of the method remains incompletely demonstrated.

My primary concern in the original review was the generalizability of MASD-seq beyond colorectal cancer. The authors now include analyses across the ICGC dataset and additional MspI-RRS data from gastric cancer and hepatocellular carcinoma, which are valuable additions. However, these analyses mainly demonstrate differences in mutation enrichment rather than validating the performance of MASD-seq itself in additional tumour types.

Consequently, the experimental validation of the complete workflow-including ctDNA detection-remains largely confined to colorectal cancer. I therefore recommend that the authors further moderate statements regarding broad clinical applicability and explicitly state that the current experimental validation primarily supports colorectal cancer, while extension to other tumour types remains to be demonstrated prospectively.

2. The new benchmarking experiment is useful but does not fully address the clinical relevance of duplex correction.

The newly added spike-in comparison with a commercial duplex sequencing assay is an important addition and demonstrates the analytical advantages of MASD-seq under controlled conditions.

However, this experiment still represents an analytical spike-in model rather than validation in an independent clinical cohort. Therefore, while it convincingly demonstrates improved molecular recovery and analytical sensitivity, it does not fully establish the clinical impact of duplex correction for patient-derived cfDNA samples across different clinical scenarios.

RESPONSE TO REVIEWER COMMENTS

Referee #3 (Comments on Novelty/Model System for Author):

The selected model systems are appropriate for the objectives of this study. The use of GM12878 and HEK293T cell lines provides a suitable platform for technical validation of sequencing accuracy and error correction, while colorectal cancer tissues and plasma cfDNA samples allow proof-of-principle evaluation of the method in a clinically relevant setting. The inclusion of publicly available datasets to assess mutation enrichment across multiple tumour types further strengthens the manuscript.

Nevertheless, the experimental validation of the complete MASD-seq workflow remains largely confined to colorectal cancer. Although the authors now discuss the applicability of the approach to additional cancer types and provide computational analyses of mutation enrichment, experimental validation in other tumour types is still limited. Future studies evaluating MASD-seq in independent cohorts representing different mutational landscapes would strengthen the evidence for broader clinical applicability.

Response: We sincerely thank the reviewer for the positive and constructive comments.

Referee #3 (Remarks for Author):

I appreciate the authors' thorough revision and the effort invested in addressing my comments. The manuscript has been strengthened by the inclusion of additional methodological details, the comparison between EM-seq and bisulfite sequencing, the expanded figures, and the new benchmarking experiment against a duplex sequencing assay. Overall, the manuscript is improved. However, I believe that two important issues remain only partially addressed.

1. Broader applicability of the method remains incompletely demonstrated. My primary concern in the original review was the generalizability of MASD-seq beyond colorectal cancer. The authors now include analyses across the ICGC dataset and additional MspI-RRS data from gastric cancer and hepatocellular carcinoma, which are valuable additions. However, these analyses mainly demonstrate differences in mutation enrichment rather than validating the performance of MASD-seq itself in additional tumour types. Consequently, the experimental validation of the complete workflow-including ctDNA detection-remains largely confined to colorectal cancer. I therefore recommend that the authors further moderate statements regarding broad clinical applicability and explicitly state that the current experimental validation primarily supports colorectal cancer, while extension to other tumour types remains to be demonstrated prospectively.

Response: We thank the reviewer for this comment. We acknowledge that, due to time and resource constraints, our analyses of broader applicability of the method restricted to the in silico data the ICGC dataset and additional MspI-RRS data from gastric cancer and hepatocellular carcinoma. We recognize that these analyses do not constitute direct experimental validation of MASD-seq itself in other tumor types. We have clarified this limitation in Discussion (Line 501-503 as follows: "the current experimental validation supports the clinical applicability of MASD-seq only in colorectal cancer, while its applicability to other tumor types remains to be validated."

2. The new benchmarking experiment is useful but does not fully address the clinical relevance of duplex correction. The newly added spike-in comparison with a commercial duplex sequencing assay is an important addition and demonstrates the analytical advantages of MASD-seq under controlled conditions. However, this experiment still represents an analytical spike-in model rather than validation in an independent clinical cohort. Therefore, while it convincingly demonstrates improved molecular recovery and analytical sensitivity, it does not fully establish the clinical impact of duplex correction for patient-derived cfDNA samples across different clinical scenarios.

Response: We thank the reviewer for this comment. We completely agree that clinical relevance cannot be fully established by spike-in experiments alone. Our study was designed for concept proposal, methodological establishment, and analytical performance evaluation. The controlled spike-in approach allowed us to demonstrate the analytical advantages of MASD-seq without the confounding factors such as sample heterogeneity. While we recognize the need for clinical cohort validation, this required substantially more resources and was beyond the predefined scope of this project. We have clarified this limitation in Discussion (Line 498-501) as follows: "Although we demonstrate analytical advantages of MASD-seq using a controlled spike-in model with a commercial duplex sequencing assay, these findings are derived from reference materials rather than from clinical patient-derived cfDNA samples."

4th Aug 2026

Dear Dr. Wen,

We are pleased to inform you that your manuscript is accepted for publication and is now being sent to our publisher to be included in the next available issue of EMBO Molecular Medicine.

Your manuscript will be processed for publication by EMBO Press. It will be copy edited and you will receive page proofs prior to publication. Please note that you will be contacted by Springer Nature Author Services to complete licensing and payment information.

You may qualify for financial assistance for your publication charges - either via a Springer Nature fully open access agreement or an EMBO initiative. Check your eligibility: https://link.springer.com/journal/44321/how-to-publish-with-us

Should you be planning a Press Release on your article, please notify the production team via this form in order to coordinate publication and release dates: https://forms.cloud.microsoft/e/eH4A8JVP6g

If you have any questions, please do not hesitate to contact the Editorial Office. Thank you for your contribution to EMBO Molecular Medicine.

Sincerely,
Jingyi

Jingyi Hou
Senior Editor
EMBO Molecular Medicine

>>> Please note that it is EMBO Molecular Medicine policy for the transcript of the editorial process (containing referee reports and your response letter) to be published as an online supplement to each paper. If you do NOT want this, you will need to inform the Editorial Office via email immediately. More information is available here: https://link.springer.com/partners/embo-press/editorial-policies#Peer%20review

EMBO Press Author Checklist

Corresponding Author Name:Wei Fu, Xin Zhou, Lu Wen	
Journal Submitted to: EMBO Molecular Medicine	
Manuscript Number: EMM-2025-23200-V2	

USEFUL LINKS FOR COMPLETING THIS FORM
The EMBO Journal - Author Guidelines
EMBO Reports - Author Guidelines
Molecular Systems Biology - Author Guidelines
EMBO Molecular Medicine - Author Guidelines

Reporting Checklist for Life Science Articles (updated January
This checklist is adapted from Materials Design Analysis Reporting (MDAR) Checklist for Authors. MDAR establishes a minimum set of requirements in transparent reporting in the life sciences (see Statement of Task: 10.31222/osf.io/9sm4x). Please follow the journal's guidelines in preparing your
Please note that a copy of this checklist will be published alongside your article.

Abridged guidelines for figures
1. Data
The data shown in figures should satisfy the following conditions:
- the data were obtained and processed according to the field's best practice and are presented to reflect the results of the experiments in an accurate and unbiased manner.
- ideally, figure panels should include only measurements that are directly comparable to each other and obtained with the same assay.
- plots include clearly labeled error bars for independent experiments and sample sizes. Unless justified, error bars should not be shown for technical
- if n<5, the individual data points from each experiment should be plotted. Any statistical test employed should be justified.
- Source Data should be included to report the data underlying figures according to the guidelines set out in the authorship guidelines on Data

2. Captions
Each figure caption should contain the following information, for each panel where they are relevant:
- a specification of the experimental system investigated (eg cell line, species name).
- the assay(s) and method(s) used to carry out the reported observations and measurements.
- an explicit mention of the biological and chemical entity(ies) that are being measured.
- an explicit mention of the biological and chemical entity(ies) that are altered/varied/perturbed in a controlled manner.
- the exact sample size (n) for each experimental group/condition, given as a number, not a range;
- a description of the sample collection allowing the reader to understand whether the samples represent technical or biological replicates (including how many animals, litters, cultures, etc.).
- a statement of how many times the experiment shown was independently replicated in the laboratory.
- definitions of statistical methods and measures:
  - common tests, such as t-test (please specify whether paired vs. unpaired), simple χ2 tests, Wilcoxon and Mann-Whitney tests, can be unambiguously identified by name only, but more complex techniques should be described in the methods section;
  - are tests one-sided or two-sided?
  - are there adjustments for multiple comparisons?
  - exact statistical test results, e.g., P values = x but not P values < x;
  - definition of 'center values' as median or average;
  - definition of error bars as s.d. or s.e.m.

Please complete ALL of the questions below.
Select "Not Applicable" only when the requested information is not relevant for your study.

Materials

Newly Created Materials	Information included in the manuscript?	In which section is the information available? (Reagents and Tools Table, Materials and Methods, Figures, Data Availability Section)
New materials and reagents need to be available; do any restrictions apply?	Yes	Methods and Appendix PDF

Antibodies	Information included in the manuscript?	In which section is the information available? (Reagents and Tools Table, Materials and Methods, Figures, Data Availability Section)
For **antibodies** provide the following information:<br>- Commercial antibodies: RRID (if possible) or supplier name, catalogue number and or/clone number<br>- Non-commercial: RRID or citation	Not Applicable	

DNA and RNA sequences	Information included in the manuscript?	In which section is the information available? (Reagents and Tools Table, Materials and Methods, Figures, Data Availability Section)
**Short novel DNA or RNA including primers, probes**: provide the sequences.	Yes	Methods, Appendix PDF, Appendix PDF

Cell materials	Information included in the manuscript?	In which section is the information available? (Reagents and Tools Table, Materials and Methods, Figures, Data Availability Section)
**Cell lines:** Provide species information, strain. Provide accession number in repository **OR** supplier name, catalog number, clone number, and/**OR** RRID.	Not Applicable	
**Primary cultures:** Provide species, strain, sex of origin, genetic modification status.	Not Applicable	
Report if the cell lines were recently **authenticated** (e.g., by STR profiling) and tested for mycoplasma contamination.	Not Applicable	

Experimental animals	Information included in the manuscript?	In which section is the information available? (Reagents and Tools Table, Materials and Methods, Figures, Data Availability Section)
**Laboratory animals or Model organisms:** Provide species, strain, sex, age, genetic modification status. Provide accession number in repository **OR** supplier name, catalog number, clone number, **OR** RRID.	Not Applicable	
**Animal observed in or captured from the field:** Provide species, sex, and age where possible.	Not Applicable	
Please detail **housing and husbandry conditions**.	Not Applicable	

Plants and microbes	Information included in the manuscript?	In which section is the information available? (Reagents and Tools Table, Materials and Methods, Figures, Data Availability Section)
**Plants:** provide species and strain, ecotype and cultivar where relevant, unique accession number if available, and source (including location for collected wild specimens).	Not Applicable	
**Microbes:** provide species and strain, unique accession number if available, and source.	Not Applicable	

Human research participants	Information included in the manuscript?	In which section is the information available? (Reagents and Tools Table, Materials and Methods, Figures, Data Availability Section)
If collected and within the bounds of privacy constraints report on age, sex and gender or ethnicity for all study participants.	Yes	Methods

Core facilities	Information included in the manuscript?	In which section is the information available? (Reagents and Tools Table, Materials and Methods, Figures, Data Availability Section)
If your work benefited from core facilities, was their service mentioned in the acknowledgments section?	Not Applicable	

Design

Study protocol	Information included in the manuscript?	In which section is the information available? (Reagents and Tools Table, Materials and Methods, Figures, Data Availability Section)
If study protocol has been **pre-registered, provide DOI in the manuscript**. For clinical trials, provide the trial registration number **OR** cite DOI.	Yes	Methods and Appendix PDF
Report the **clinical trial registration number** (at ClinicalTrials.gov or equivalent), where applicable.	Not Applicable	

Laboratory protocol	Information included in the manuscript?	In which section is the information available? (Reagents and Tools Table, Materials and Methods, Figures, Data Availability Section)
Provide DOI OR other citation details if **external detailed step-by-step protocols** are available.	Not Applicable	

Experimental study design and statistics	Information included in the manuscript?	In which section is the information available? (Reagents and Tools Table, Materials and Methods, Figures, Data Availability Section)
Include a statement about **sample size** estimate even if no statistical methods were used.	Not Applicable	
Were any steps taken to minimize the effects of subjective bias when allocating animals/samples to treatment (e.g. **randomization procedure**)? If yes, have they been described?	Not Applicable	
Include a statement about **blinding** even if no blinding was done.	Not Applicable	
Describe **inclusion/exclusion criteria** if samples or animals were excluded from the analysis. Were the criteria pre-established?<br><br>If sample or data points were omitted from analysis, report if this was due to attrition or intentional exclusion and provide justification.	Not Applicable	
For every figure, are **statistical tests** justified as appropriate? Do the data meet the assumptions of the tests (e.g., normal distribution)? Describe any methods used to assess it. Is there an estimate of variation within each group of data? Is the variance similar between the groups that are being statistically compared?	Yes	Results and methods

Sample definition and in-laboratory replication	Information included in the manuscript?	In which section is the information available? (Reagents and Tools Table, Materials and Methods, Figures, Data Availability Section)
In the figure legends: state number of times the experiment was **replicated** in laboratory.	Yes	Results and methods
In the figure legends: define whether data describe **technical or biological replicates**.	Yes	Results and methods

Ethics

Ethics	Information included in the manuscript?	In which section is the information available? (Reagents and Tools Table, Materials and Methods, Figures, Data Availability Section)
Studies involving **human participants**: State details of **authority granting ethics approval** (IRB or equivalent committee(s), provide reference number for approval.	Yes	methods
Studies involving **human participants**: Include a statement confirming that **informed consent** was obtained from all subjects and that the experiments conformed to the principles set out in the WMA Declaration of Helsinki and the Department of Health and Human Services Belmont Report.	Yes	methods
Studies involving **human participants: For publication of patient photos**, include a statement confirming that consent to publish was obtained.	Not Applicable	
Studies involving experimental **animals**: State details of **authority granting ethics approval** (IRB or equivalent committee(s), provide reference number for approval. Include a statement of compliance with ethical regulations.	Not Applicable	
Studies involving **specimen and field samples:** State if relevant **permits** obtained, provide details of authority approving study; if none were required, explain why.	Not Applicable	

Dual Use Research of Concern (DURC)	Information included in the manuscript?	In which section is the information available? (Reagents and Tools Table, Materials and Methods, Figures, Data Availability Section)
Could your study fall under dual use research restrictions? Please check biosecurity documents and list of **select agents and toxins** (CDC): https://www.selectagents.gov/sat/list.htm	Not Applicable	
If you used a select agent, is the security level of the lab appropriate and reported in the manuscript?	Not Applicable	
If a study is subject to dual use research of concern regulations, is the name of the **authority granting approval and reference number** for the regulatory approval provided in the manuscript?	Not Applicable	

Reporting

The MDAR framework recommends adoption of discipline-specific guidelines, established and endorsed through community initiatives. Journals have their own policy about requiring specific guidelines and recommendations to complement MDAR.

Adherence to community standards	Information included in the manuscript?	In which section is the information available? (Reagents and Tools Table, Materials and Methods, Figures, Data Availability Section)
State if relevant guidelines or checklists (e.g., **ICMJE, MIBBI, ARRIVE, PRISMA**) have been followed or provided.	Not Applicable	
For **tumor marker prognostic studies,** we recommend that you follow the **REMARK** reporting guidelines (see link list at top right). See author guidelines, under 'Reporting Guidelines'. Please confirm you have followed these guidelines.	Not Applicable	
For **phase II and III randomized controlled trials**, please refer to the **CONSORT** flow diagram (see link list at top right) and submit the CONSORT checklist (see link list at top right) with your submission. See author guidelines, under 'Reporting Guidelines'. Please confirm you have submitted this list.	Not Applicable	

Data Availability

Data availability	Information included in the manuscript?	In which section is the information available? (Reagents and Tools Table, Materials and Methods, Figures, Data Availability Section)
Have **primary datasets** been deposited according to the journal's guidelines (see 'Data Deposition' section) and the respective accession numbers provided in the Data Availability Section?	Yes	methods
Were **human clinical and genomic datasets** deposited in a public access-controlled repository in accordance to ethical obligations to the patients and to the applicable consent agreement?	Yes	methods
Are **computational models** that are central and integral to a study available without restrictions in a machine-readable form? Were the relevant accession numbers or links provided?	Yes	methods
If publicly available data were reused, provide the respective **data citations in the reference list**.	Yes	reference
